# C–H, N–H, and O–H Bond Activations to Prepare Phosphorescent Hydride-Iridium(III)-Phosphine Emitters with Photocatalytic Achievement in C–C Coupling Reactions

**DOI:** 10.1021/acs.inorgchem.4c00115

**Published:** 2024-03-28

**Authors:** María Benítez, María L. Buil, Miguel A. Esteruelas, Ana M. López, Cristina Martín-Escura, Enrique Oñate

**Affiliations:** Departamento de Química Inorgánica, Instituto de Síntesis Química y Catálisis Homogénea (ISQCH), Centro de Innovación en Química Avanzada (ORFEO-CINQA), 111303 Universidad de Zaragoza-CSIC, Zaragoza 50009, Spain

## Abstract

Complex IrH_5_(P^i^Pr_3_)_2_ (**1**) activates two different σ-bonds of 3-phenoxy-1-phenylisoquinoline, 2-(1*H*-benzimidazol-2-yl)-6-phenylpyridine, 2-(1*H*-indol-2-yl)-6-phenylpyridine, 2-(2-hydroxyphenyl)-6-phenylpyridine, *N*-(2-hydroxyphenyl)-*N*′-phenylimidazolylidene, and 1,3-di­(2-pyridyl)-4,6-dimethylbenzene to give IrH­{κ^3^-*C,N,C*-[C_6_H_4_-isoqui-O-C_6_H_4_]}­(P^i^Pr_3_)_2_ (**2**), IrH­{κ^3^-*N,N,C*-[NBzim-py-C_6_H_4_]}­(P^i^Pr_3_)_2_ (**3**), IrH­{κ^3^-*N,N,C*-[Ind-py-C_6_H_4_]}­(P^i^Pr_3_)_2_ (**4**), IrH­{κ^3^-*C,N,O*-[C_6_H_4_-py-C_6_H_4_O]}­(P^i^Pr_3_)_2_ (**5**), IrH­{κ^3^-*C,C,O*-[C_6_H_4_-Im-C_6_H_4_O]}­(P^i^Pr_3_)_2_ (**6**), and IrH­{κ^3^-*N,C,C*-[py-C_6_HMe_2_-C_5_H_3_N]}­(P^i^Pr_3_)_2_ (**7**), respectively. The activations are sequential, with the second generally being the slowest. Accordingly, dihydride intermediates IrH_2_{κ^2^-*C,N*-[C_6_H_4_-isoqui-O-C_6_H_5_]}­(P^i^Pr_3_)_2_ (**2d**), IrH_2_{κ^2^-*N,N*-[NBzim-py-C_6_H_5_]}­(P^i^Pr_3_)_2_ (**3d**), IrH_2_{κ^2^-*N,N*-[Ind-py-C_6_H_5_]}­(P^i^Pr_3_)_2_ (**4d**), and IrH_2_{κ^2^-*N,C*-[py-C_6_HMe_2_-py]}­(P^i^Pr_3_)_2_ (**7d**) were characterized spectroscopically. Complexes **3** and **5** are green phosphorescent emitters upon photoexcitation, exhibiting good absorption over a wide range of wavelengths, emission quantum yields about 0.70 in solution, long enough lifetimes (10–17 μs), and reversible electrochemical behavior. In agreement with these features, complex **3** promotes the photocatalytic α-amino C­(sp^3^)–H arylation of *N*,*N*-dimethylaniline and *N*-phenylpiperidine with 1,4-dicyanobenzene and 4-cyanopyridine under blue LED light irradiation. The C–C coupling products are isolated in high yields with only 2 mol % of photocatalyst after 24 h.

## Graphical abstract

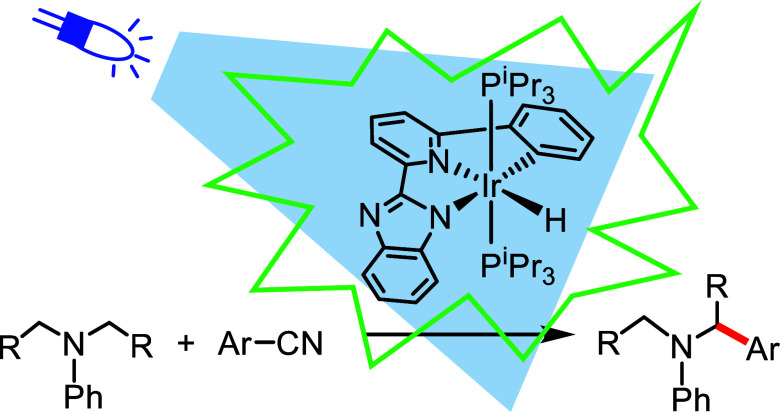

## Introduction

There is great interest in the search for new iridium­(III) phosphorescent emitters mainly for two reasons. The high spin–orbit coupling constant effect of the 5d-metal results in fast intersystem crossing between the S_1_-T_1_ excited states, allowing them to collect singlet and triplet excitons and achieve internal quantum efficiencies close to 100%.^1^ Furthermore, emissions show ligand dependence; therefore, the rational design of an emitter to achieve a given application should, in principle, be feasible by a judicious combination of the groups that form the coordination sphere around the iridium center.^2^

The most common design has been octahedral coordination spheres formed by three bidentate 3e donor ligands, two or three of them different. However, the use of this class of compounds presents two drawbacks: a high number of structural isomers with different photophysical properties and a marked thermodynamic tendency to form equilibrium mixtures of compounds resulting from ligand redistribution reactions.^3^ In line with the impact of pincer ligands in transition metal chemistry,^4^ a promising alternative to the application of bidentate groups is the employment of pincer donors of 5e and 4e.^5^ The coordination of both ligands to the same iridium­(III) center gives rise to six-coordinate complexes, which do not suffer the same problems as those formed by different bidentate groups.^6^ Although the rigidity imposed by these ligands could generate distortions in the emitter structure, leading to a decrease in emission efficiency, it is also true that the increase in the strength of the metal–ligand interaction should disfavor thermally induced quenching.^5b^

One way to partly avoid the distortion imposed by the coordination of both pincers is to release the donor atoms of the 5e-pincer. In this context, the replacement of said pincer by two basic phosphine groups, arranged mutually *trans*, and a hydride ligand seems in principle an interesting alternative. The presence of such ligands causes a large intensity of the ligand field. This situation destabilizes the metal-centered *dd* excited states, which guide radiationless deactivation processes as a consequence of their repulsive potential energy surface.^1e^ Furthermore, the HOMO–LUMO gap increases, which produces a shift of the emission toward higher energies. High Δ_0_ values also prevent ligand dissociations, decreasing possible emitter decomposition chemical pathways.^7^ Indeed, a few emitters carrying a coordination sphere formed by a pincer donor of 4e, two phosphines, and a hydride have recently been reported to be particularly efficient members^8^ of a small family of phosphine-derived emitters.^9^

Molecules in the excited state are more powerful oxidizing and reducing agents than in their ground state. As a consequence, photoexcitation of a phosphorescent emitter in its ground state S_0_ generates an excited state T_1_, which can accept an electron from an organic molecule or donate it to the latter. After this initial photoinduced single-electron-transfer process, the respective reducing or oxidizing form of the emitter can perform a new single electron transfer event with another organic reagent. This second redox reaction leads to an opposite radical, in addition to reforming the emitter in its ground state S_0_, closing a photoredox cycle. At the same time, the coupling of the radicals gives rise to a new neutral organic molecule.^10^ Iridium­(III) phosphorescent emitters are no exception^11^ and promote challenging organic reactions.^12^ In fact, interesting C–H functionalization reactions are photoinduced using complexes of this class that contain three bidentate ligands.^13^ In 2011, MacMillan et al. observed that the homoleptic complex Ir­{κ^2^-*C,N*-[2-C_6_H_4_-py]}_3_ bearing three orthometalated 2-phenylpyridine groups catalyzes the coupling of cyanobenzenes with α-amino radicals, in the presence of a base, upon photoexcitation.^14^ Houk, Mayer, Ellman, and co-workers have recently reported a diastereoselective version of the reaction.^15^ The couplings generate valuable benzylic amine products. The proposed mechanism for such α-amino C­(sp^3^)–H arylations (Scheme 1) proceeds through a cycle involving Ir­(III) and [Ir­(IV)]^+^ species. Photoexcitation of the ground state of the catalyst, ^S0^Ir­(III), generates an excited state ^T1^Ir­(III), which reduces the cyanobenzenes to the corresponding anionic radicals providing the oxidant Ir­(IV)-cation. Then, a single-electron-transfer between the latter and the amines produces cationic amine radicals and regenerates ^S0^Ir­(III). The α-C–H bonds of these cationic radicals are extremely acidic and susceptible to easy deprotonation. The proton abstraction leads to a neutral α-amino radical. Coupling of the latter with the cyanobenzene radical, followed by loss of a cyanide anion, provides the α-arylated product.^14^

**Scheme 1:**
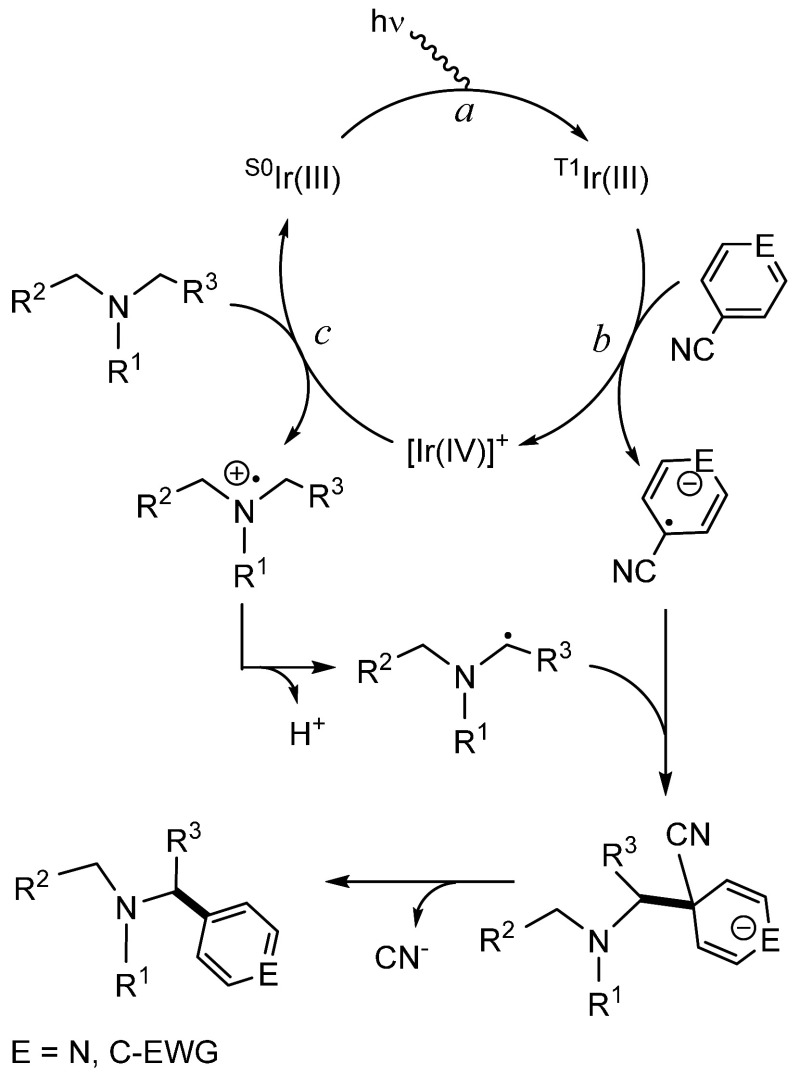
Proposed Mechanism for Photoredox α-Amino C­(sp^3^)–H Arylation with Cyanobenzenes Catalyzed by Ir­{κ^2^-*C,N*-[2-C_6_H_4_-py]}_3_

Polyhydride complexes of platinum group metals are particularly useful in promoting the activation of σ-bonds.^16^ Related to this ability is its relevance in catalysis^17^ and its use to prepare challenging organometallic complexes with interesting photoluminescent properties.^18^ Iridium pentahydride IrH_5_(P^i^Pr_3_)_2_ (**1**) is a member of this class of compounds that possess such qualities.^3v,8b,9f,19^ In the search for the first iridium­(III) phosphorescent emitters based on the *trans*-IrH­(P^i^Pr_3_)_2_ moiety, which present photocatalytic activity, we have studied the reactivity of said polyhydride against the pro-pincer organic compounds shown in Chart 1. This article presents the members of a family of iridium­(III) phosphorescent emitters, *trans*-IrH­(κ^3^-L)­(P^i^Pr_3_)_2_, resulting from the studied reactions, which includes a photocatalyst for the α-amino C­(sp^3^)–H arylation.

**Chart 1:**
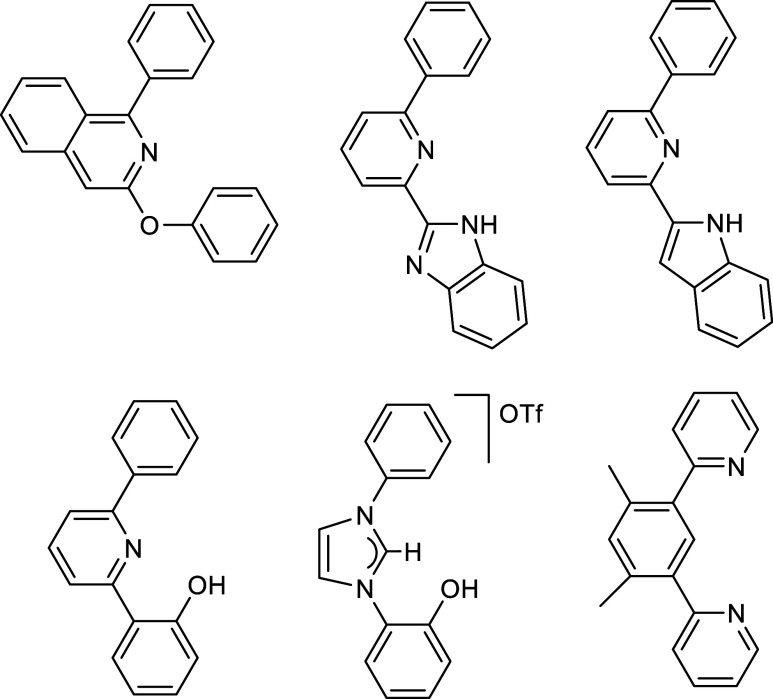
Pro-Pincer Ligands Used in This Study.

## Results and Discussion

### σ-Bond Activation Reactions: Preparation of the Emitters

Scheme 2 brings together the prepared members of the IrH­(κ^3^-L)­(P^i^Pr_3_)_2_ family, complexes IrH­{κ^3^-*C,N,C*-[C_6_H_4_-isoqui-O-C_6_H_4_]}­(P^i^Pr_3_)_2_ (**2**), IrH­{κ^3^-*N,N,C*-[NBzim-py-C_6_H_4_]}­(P^i^Pr_3_)_2_ (**3**), IrH­{κ^3^-*N,N,C*-[Ind-py-C_6_H_4_]}­(P^i^Pr_3_)_2_ (**4**), IrH­{κ^3^-*C,N,O*-[C_6_H_4_-py-C_6_H_4_O]}­(P^i^Pr_3_)_2_ (**5**), IrH­{κ^3^-*C,C,O*-[C_6_H_4_-Im-C_6_H_4_O]}­(P^i^Pr_3_)_2_ (**6**), and IrH­{κ^3^-*N,C,C*-[py-C_6_HMe_2_-C_5_H_3_N]}­(P^i^Pr_3_)_2_ (**7**). They include compounds stabilized by five different types of pincer ligands, namely: *C,N,C*′; *N,N*′,*C*; *C,N,O*; *C,C*′,*O*; and *N,C,C*′. With the exception of complex **7**, these complexes result from the sequential activation of a σ bond of the substituents, in positions adjacent to the 2e-donor atom, of heterocycles such as isoquinoline, pyridine, and imidazolylidene and from the coordination of said donor atom of the central ring. Complex **7** has the positions of the 2e-donor heterocycle and one of the activated groups exchanged. Table 1 shows the most characteristic resonances of **2**–**7** in their NMR spectra. In addition to the triplets due to the metalated carbon atoms of the pincer ligands, in the ^13^C­{^1^H} NMR spectra, notable signals are a triplet due to the hydride ligand, in the high-field region of the ^1^H NMR spectra, and a singlet corresponding to the equivalent phosphines, which splits into a doublet under off-resonance conditions, in the ^31^P­{^1^H} NMR spectra. In agreement with the sequencing of the activations, the dihydride intermediates IrH_2_{κ^2^-*C,N*-[C_6_H_4_-isoqui-O-C_6_H_5_]}­(P^i^Pr_3_)_2_ (**2d**), IrH_2_{κ^2^-*N,N*-[NBzim-py-C_6_H_5_]}­(P^i^Pr_3_)_2_ (**3d**), IrH_2_{κ^2^-*N,N*-[Ind-py-C_6_H_5_]}­(P^i^Pr_3_)_2_ (**4d**), and IrH_2_{κ^2^-*N,C*-[py-C_6_HMe_2_-py]}­(P^i^Pr_3_)_2_ (**7d**) were spectroscopically detected and characterized. The inequivalent hydrides of these intermediates generate two doublets of triplets in the ^1^H NMR spectra, while the equivalent phosphines give rise to a singlet, which splits into a triplet under off-resonance conditions, in the ^31^P­{^1^H} NMR spectra (Table 2).

**Scheme 2:**
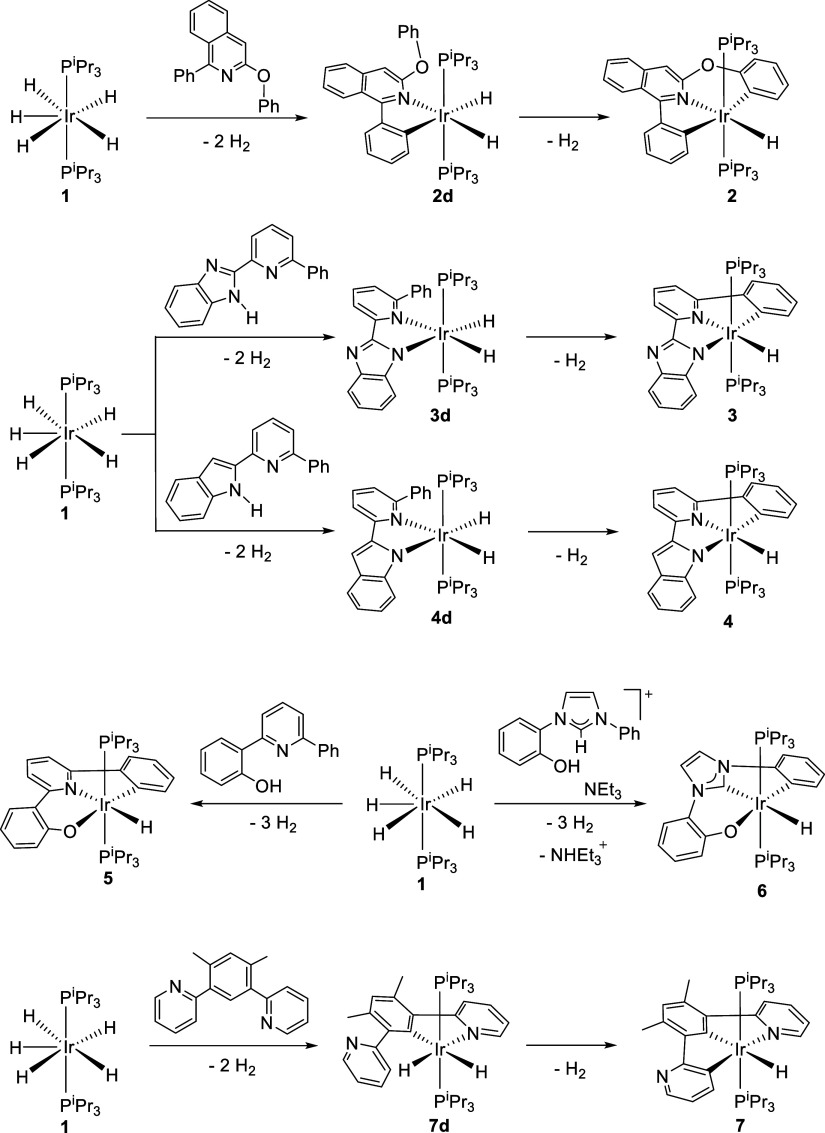
Synthesis of Complexes IrH­(κ^3^-L)­(P^i^Pr_3_)_2_ (**2–7**)

**Table 1:** Selected Resonance Signals of **2**–**7** in Their ^13^C­{^1^H}, ^1^H, and ^31^P­{^1^H}-NMR Spectra in C_6_D_6_ at 298 K^a^

complex	^13^C{^1^H} δ Ir–C (^2^ *J*_CP_)	^1^H δ Ir–H (^2^ *J*_HP_)	^31^P{^1^H} δ Ir–P
**2**	171.8 (8.8)	–18.37 (19.5)	2.3
	131.0 (9.4)		
**3**	146.3 (6.4)	–14.86 (19.4)	5.0
**4**	147.4 (7.8)	–14.83 (19.7)	4.4
**5**	146.9 (7.3)	–16.49 (18.2)	13.9
**6**	177.9 (4.5)	–8.02 (20.4)	21.9
	124.7 (7.0)		
**7**	195.0 (4.3)	–8.17 (22.8)	8.7
	138.0(9.4)		

a δ in ppm and *J* in Hertz.

**Table 2:** Selected Resonance Signals for the Dihydride Complexes in Their ^1^H and ^31^P­{^1^H}-NMR Spectra in C_6_D_6_ at 298 K^a^

complex	^1^H NMR			^31^P{^1^H}
	δ Ir–H	^2^ *J*_HP_	^2^ *J*_HH_	δ Ir–P
**2d**	–12.55	20.6	5.4	30.3
	–21.82	18.3		
**3d**	–20.35	17.1	7.0	20.7
	–23.18	18.4		
**4d** ^b^	–11.59	16.0	6.8	45.9
	–12.64	19.0		
**7d** ^b^	–12.74	21.5	4.4	21.8
	–20.91	18.5		
**8**	–14.03	20.6	4.3	28.4
	–14.31	19.7		

a δ in ppm and *J* in Hertz.

b In toluene.

The activation of a σ-bond of an organic molecule promoted by an unsaturated metal fragment occurs in two steps: coordination of the σ-bond and its subsequent cleavage.^20^ Therefore, the activation energy of the activation depends on two factors: the stability of the particular σ-intermediate and the strength of the coordinated bond.^21^ The breaking of the coordinated σ-bond has been proposed to be homolytic or heterolytic depending on the electronic nature of the metal center. Basic centers promote the former, while acidic centers promote the latter with the help of an internal or external base.^16,22^ Furthermore, it is worth mentioning that the activation of σ-bonds of organic coordinating molecules can be assisted by a chelating effect or atom-directed. The cleavage of the σ-bond occurs before the coordination of the assistant donor atom in the first case,^19b,23^ while in the second, the opposite sequence takes place, that is, the directing group coordinates first.^24^ Complex **1** is a saturated species, which reductively eliminates a hydrogen molecule to produce the unsaturated trihydride IrH_3_(P^i^Pr_3_)_2_ (**A**), which is responsible for initiating the σ-bond activation processes promoted by **1**. Scheme 3 shows a general rationalization of the products formed according to Scheme 2. Coordination of one of the σ- bonds of the pro-ligands to the iridium­(III) acid center of **A** would generate σ-intermediates **B**. Thus, heterolytic cleavage of the coordinated bond using a basic hydride could give the dihydride-iridium­(III)-dihydrogen species **C**, which should lead to dihydrides **D**, through replacement of the coordinated hydrogen molecule by the 2e-donor group. These dihydride type compounds are again saturated. Thus, they need to reductively remove molecular hydrogen to produce the iridium­(I) derivatives **E**. Then, the basic center of **E** should promote the homolytic oxidative addition of the second activated σ-bond to yield the products IrH­(κ^3^-L)­(P^i^Pr_3_)_2_, through the five-coordinate σ-intermediate **F**. In other words, the first σ-bond activation should involve a chelate-assisted heterolytic cleavage, while the second cleavage would be an atom-directed homolytic oxidative addition.

**Scheme 3:**
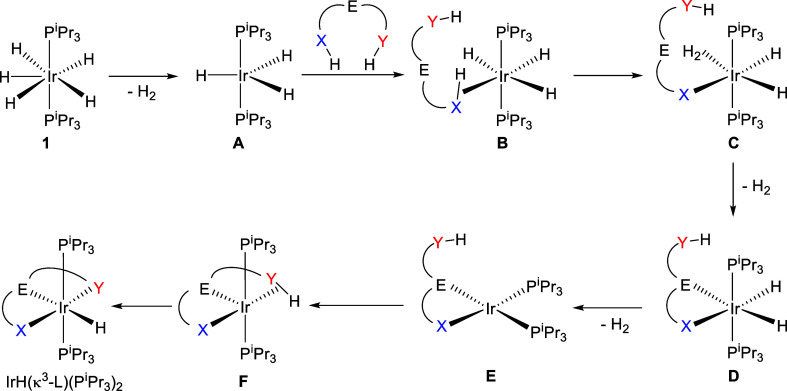
General Pathway for the Formation of Complexes IrH­(κ^3^-L)­(P^i^Pr_3_)_2_

The formation process of **2** is slow. In toluene, under reflux, it took a week to complete and at the end of the reaction a significant degree of decomposition was observed. Thus, complex **2** was isolated as an orange solid, in moderate yield (32%), after purifying the crude oil by column chromatography on deactivated silica gel. After 24 h of reaction, a mixture of **1**, **2d**, and **2** was formed in a molar ratio of 15:54:31. Colorless crystals of **2d** suitable for X-ray diffraction analysis were obtained from the mixture. Figure 1 shows a view of the structure, demonstrating the C–H activation of the phenyl group and the free nature of the phenoxy substituent. The coordination polyhedron around the iridium­(III) center is the expected distorted octahedron, with the phosphine ligands arranged in mutually *trans* positions [P(1)–Ir–P(2) = 161.42(2)°], while the chelate resulting from assisted C–H bond activation is placed *trans* to the inequivalent hydrides [C(1)–Ir–H(01) = 174.9(10) and N(1)–Ir–H(02) = 173.7(10)], in a plane perpendicular to the P–Ir–P direction. The distortion of the ideal octahedron is mainly due to the small C(1)–Ir–N(1) bite angle of the chelate, 77.21(9)°.

**Figure 1:**
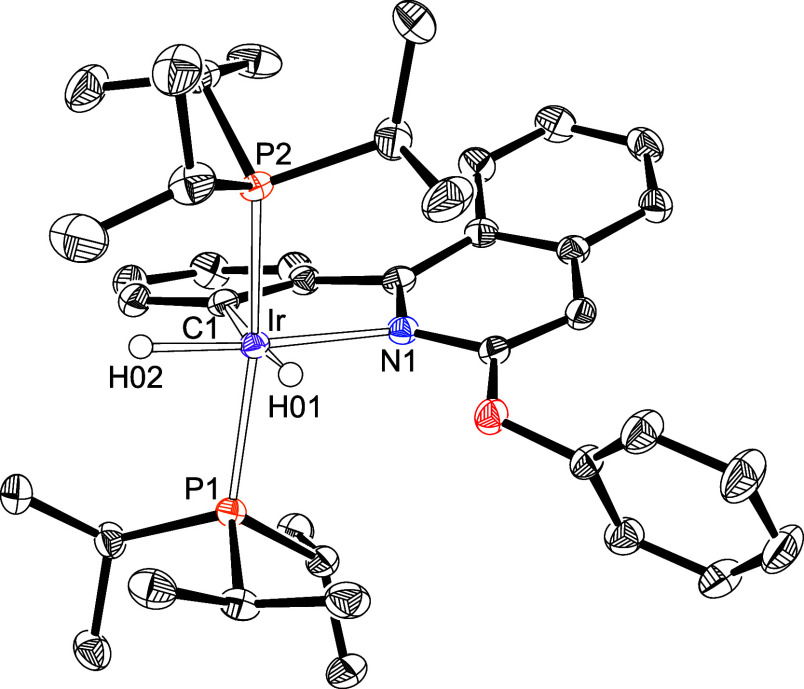
X-ray structure of **2d** (50% probability ellipsoids; hydrogen atoms, except hydride ligands, have been omitted). Selected bond lengths (Å) and angles (deg): Ir–C(1) = 2.078(2), Ir–N(1) = 2.208(2), Ir–P(1) = 2.2985(11), Ir–P(2) = 2.3043(12), P(1)–Ir–P(2) = 161.42(2), C(1)–Ir–H(01) = 174.9(10), N(1)–Ir–H(02) = 173.7(10), C(1)–Ir–N(1) = 77.21(9).

The selective activation of a particular C–H bond of an aromatic organic molecule depends kinetically on the stability of the σ-(MCH) intermediate since the strength of the different C–H bonds is similar. Therefore, it is controlled by steric factors. First, the C–H bonds located in the sterically least hindered positions are activated. Consequently, the formation of **2** via **2d** might seem surprising since the latter results from a C–H activation of the phenyl substituent of 3-phenoxy-1-phenylisoquinoline in the presence of a sterically less hindered phenoxy group. However, it should be noted that the C–H activation of the phenyl substituent is thermodynamically favored with respect to the C–H activation of the phenoxy group, mainly because the former provides a more entropic species than the latter.

The reactions of **1** with 2-(1*H*-benzimidazol-2-yl)-6-phenylpyridine and 2-(1*H*-indol-2-yl)-6-phenylpyridine to give **3** and **4**, respectively, are significantly faster than with 3-phenoxy-1-phenylisoquinoline. Thus, the corresponding monohydrides were isolated as yellow solids, with good yields (69–77%), after 24 h, in toluene, at reflux. After 10 h, the reaction of **1** with 2-(1*H*-benzimidazol-2-yl)-6-phenylpyridine generates a mixture of the dihydride intermediate **3d** and **3** in a molar ratio of 80:20. For its part, the reaction of **1** and 2-(1*H*-indol-2-yl)-6-phenylpyridine provides a mixture of **1**, **4d**, and **4** in a molar ratio of 10:42:48, after 3 h. The ^1^H NMR spectra of the mixtures reveal the absence of any NH resonance corresponding to any metallic species. This strongly supports that, as shown in Scheme 2, the N–H activation of the bicyclic substituent of the pro-ligand is prior to the C–H activation of the phenyl group, in both cases. The initial activation of the N–H bond of the bicycle was expected given the greater polarity of the N–H bond with respect to the o*rtho*-CH bond of the phenyl substituent and the heterolytic nature of the first σ-activation of the pro-ligand. Complex **3** was characterized by X-ray diffraction analysis. The structure (Figure 2) demonstrates the formation of the *N,N*′,*C*-pincer. The donor atoms around the metal center form a distorted octahedron with *trans* arranged phosphines [P(1)–Ir–P(2) = 160.39(4)°]. The pincer acts with a C(1)–Ir–N(2) bite angle of 156.14(14)°, in the plane perpendicular to the P–Ir–P direction, and places the central pyridyl ring *trans* to the hydride ligand [H(01)–Ir–N(1) = 176.2(13)°].

**Figure 2:**
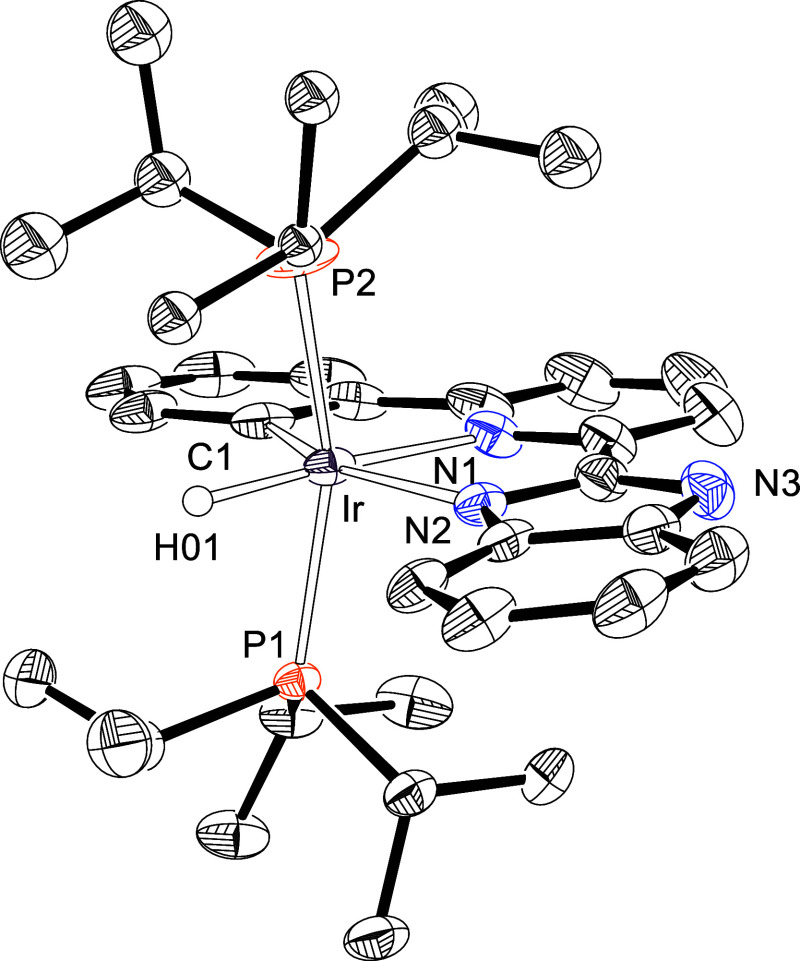
X-ray structure of **3** (50% probability ellipsoids; hydrogen atoms, except the hydride ligand, have been omitted). Selected bond lengths (Å) and angles (deg): Ir–C(1) = 2.038(3), Ir–N(1) = 2.068(3), Ir–N(2) = 2.162(2) Ir–P(1) = 2.3507(7), Ir–P(2) = 2.3451(9), P(1)–Ir–P(2) = 160.39(4), N(1)–Ir–H(01) = 176.2(13), C(1)–Ir–N(2) = 156.14(14), C(1)–Ir–N(1) = 79.46(13), N(1)–Ir–N(2) = 76.68(10).

Replacement of the bicyclic substituent of 2-(1*H*-benzimidazol-2-yl)-6-phenylpyridine and 2-(1*H*-indol-2-yl)-6-phenylpyridine by a 2-hydroxyphenyl group again increases the rate of the formation of the pincer. In refluxing toluene, the reaction of **1** with 2-(2-hydroxyphenyl)-6-phenylpyridine is complete after 16 h, approximately 8 h less than the time required for the generation of the *N,N*′,*C*-pincers. Complex **5** was isolated as a yellow solid, in 70% yield, and characterized by X-ray diffraction analysis. Figure 3 provides a view of the molecule. The coordination polyhedron around the metal center is the expected octahedron, where the pincer lies in the plane perpendicular to the P–Ir–P direction [P(1)–Ir–P(2) = 158.58(1)°] with the pyridyl group *trans* disposed to the hydride ligand [N(1)–Ir–H(01) = 178.5(8)°]. The most notable feature of the octahedron is the bite angle of the pincer, 170.93(6)° [C(1)–Ir–O(1)], which is approximately 15° greater than that observed in **3** and therefore closer to the ideal value of 180°. In this case, a dihydride intermediate of type **D** was not detected, which points out that the first σ-bond cleavage is slower than the second one and, therefore, it could be the C–H activation of the phenyl substituent. The reason is that a O–H bond is much more polar than a C–H bond and furthermore the O–H activation provides a six-membered ring with an almost ideal O(1)–Ir–N(1) angle of 90.25(5)°. In this context, it should be mentioned that the activation of the phenolic substituent does not need the reductive elimination of molecular hydrogen from the corresponding intermediate **D** of Scheme 3. Phenol is quite acidic and could protonate one of the hydrides of the latter to produce a hydride-iridium­(III)-dihydrogen species, where the coordinated hydrogen molecule would be susceptible to displacement by the oxygen atom of the resulting phenolate.

**Figure 3:**
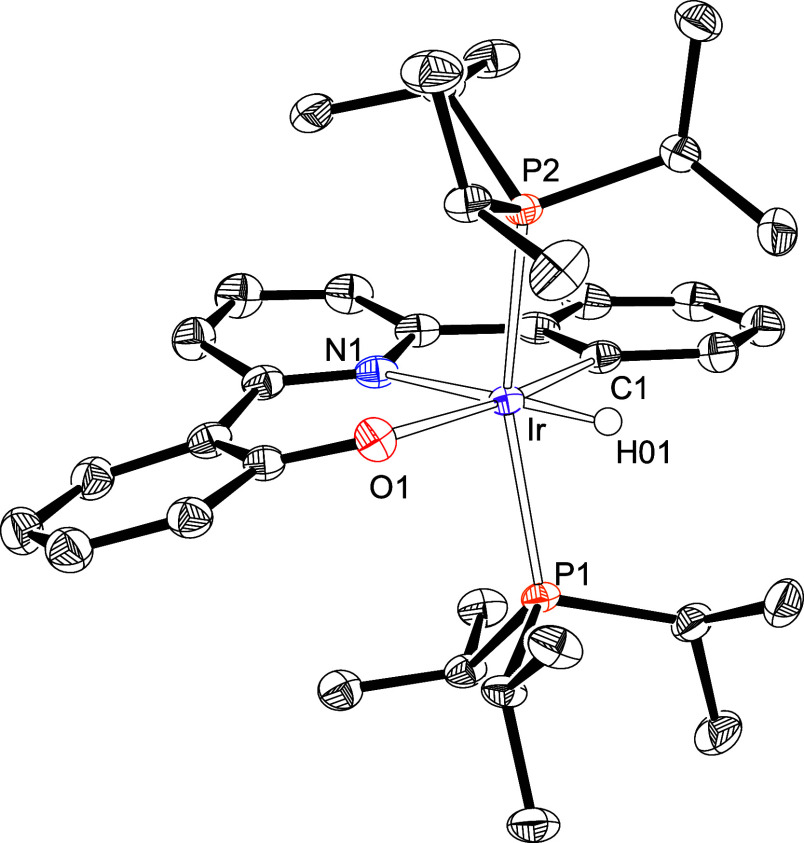
X-ray structure of **5** (50% probability ellipsoids; hydrogen atoms, except the hydride ligand, have been omitted). Selected bond lengths (Å) and angles (deg): Ir–C(1) = 2.0144(15), Ir–O(1) = 2.1172(12), Ir–N(1) = 2.1285(13), Ir–P(1) = 2.3381(4), Ir–P(2) = 2.3427(4), P(1)–Ir–P(2) = 158.58(1), N(1)–Ir–H(01) = 178.5(8), C(1)–Ir–O(1) = 170.93(6), O(1)–Ir–N(1) = 90.25(5), C(1)–Ir–N(1) = 80.86(6).

The oxygen atom of the 2-hydroxyphenyl substituent certainly plays a fundamental role in the construction of this type of *C,X,O*-pincers (X = N, C), from a thermodynamic point of view. This is evident when comparing the reactions of **1** with the triflate salts of *N*-(2-hydroxyphenyl)-*N*′-phenylimidazolium and *N*,*N*′-diphenylimidazolium cations. In toluene, at reflux, the former quickly gives **6**, which was isolated as a white solid in 66% yield, while *N,N*′-diphenylimidazolium leads to the dihydride IrH_2_{κ^3^-*C,C*,-[C_6_H_4_-Im-C_6_H_5_]}­(P^i^Pr_3_)_2_ (**8** in Scheme 4), after 1 h, as a solid white, in 40% isolated yield. Unlike intermediates **2d**–**4d** and **7d**, complex **8** is stable and does not provide the corresponding *C,C*′,*C*-pincer even in decalin, at reflux, for days. The marked stability of **8** is also evidence in favor of a rapid O–H activation of the 2-hydroxyphenyl substituent in the dihydride intermediates of type **D**.

**Scheme 4:**
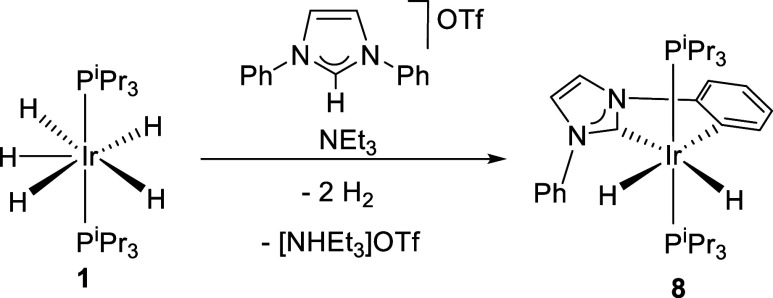
Preparation of Complex **8**

Complex **8** was characterized by X-ray diffraction analysis. The structure (Figure 4) demonstrates *ortho*-CH activation of only one of the phenyl substituents. The most notable feature of the octahedron formed by the donor atoms around the iridium center is its strong distortion caused by the small C(1)–Ir–C(5) bite angle of the chelate, 77.82(10)°. The NMR findings (Table 2) are consistent with the structure and with those of the dihydrides shown in Scheme 2.

**Figure 4:**
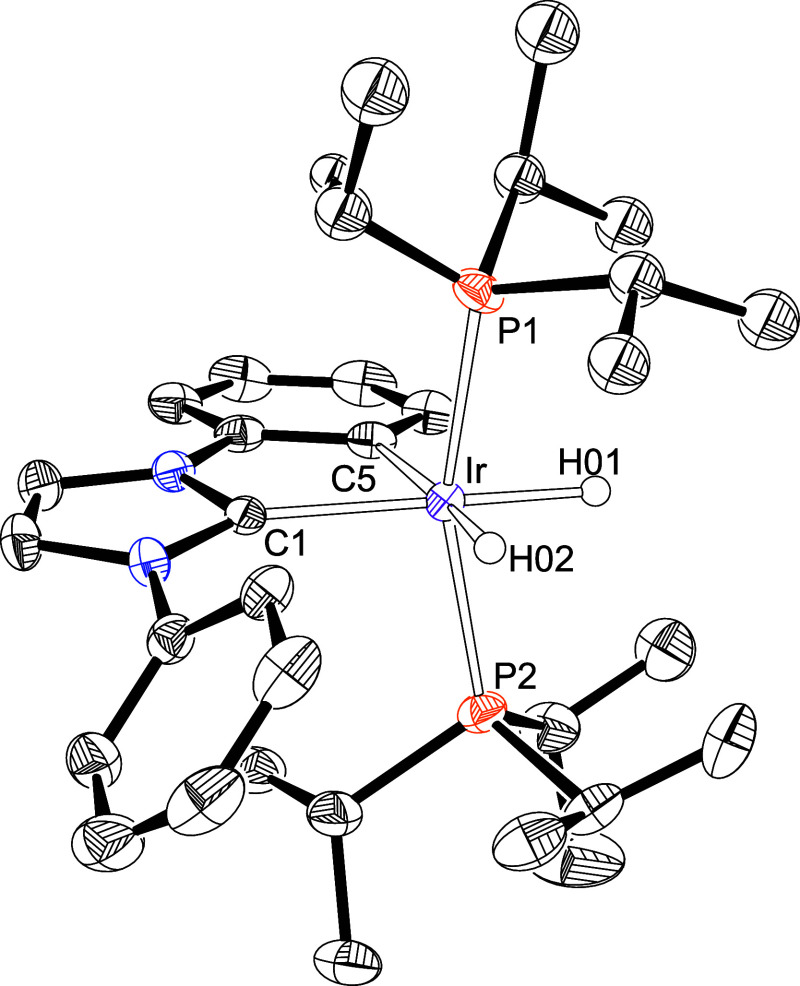
X-ray structure of **8** (50% probability ellipsoids; hydrogen atoms, except hydride ligands, have been omitted). Selected bond lengths (Å) and angles (deg): Ir–C(1) = 2.050(2), Ir–C(5) = 2.136(3), Ir–P(1) = 2.3037(7), Ir–P(2) = 2.2922(7), P(1)–Ir–P(2) = 160.50(3), C(1)–Ir–H(01) = 173.3(12), C(5)–Ir–H(02) = 174.3(12), C(1)–Ir–C(5) = 77.82(10).

1,3-Di­(2-pyridyl)-4,6-dimethylbenzene is generally the pro-ligand of an interesting 5e-donor *N,C,N*-pincer, resulting from the activation of the C–H bond in the 2-position of the central phenyl group and the *mer*-coordination of its pyridyl substituents.^25^ Said ligand plays a fundamental role in the development of heteroleptic phosphorescent iridium­(III) emitters, based on the coordination of two different pincer ligands.^6g,6j^ Complex **7** is a rare case in which a rollover-type^26^ C–H activation of one of the pyridyl groups of this pro-ligand generates a 4e-donor *N,C,C*′-pincer. Its formation requires at least 2 days and occurs with significant decomposition. Complex **7** was isolated as a yellow solid, with low yield (12%), after a tedious purification of the crude oil, which involved column chromatography on deactivated silica gel and subsequently several washes of the residue obtained with diethyl ether. The rollover-type CH activation of one of the pyridyl groups was confirmed by X-ray diffraction analysis. Figure 5 shows a view of the octahedral structure, which resembles that of the other members of the family. This particular pincer acts with a C(1)–Ir–N(1) bite angle of 157.90(9)°.

**Figure 5:**
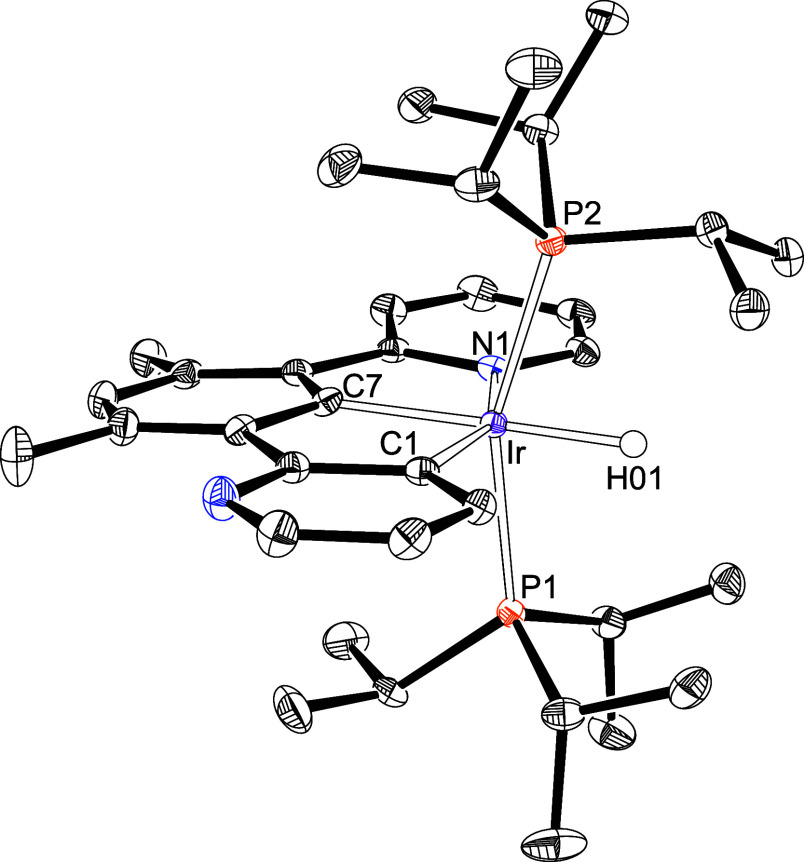
X-ray structure of **7** (50% probability ellipsoids; hydrogen atoms, except the hydride ligand, have been omitted). Selected bond lengths (Å) and angles (deg): Ir–C(1) = 2.053(2), Ir–C(7) = 2.014(3), Ir–N(1) = 2.135(2), Ir–P(1) = 2.3355(6), Ir–P(2) = 2.3415(6), P(1)–Ir–P(2) = 157.72(2), C(1)–Ir–N(1) = 157.90(9), C(7)–Ir–H(01) = 179.4(10).

The rollover-type C–H activation of the pyridyl group, which appears to be the last step of the pincer formation process, deserves some additional comment. Reductive elimination of the molecular hydrogen from **7d** should lead to an *N,C,N*-pincer-iridium­(I) intermediate, Ir­{κ^3^-*N,C,N*-[py-C_6_HMe_2_-py]}­(P^i^Pr_3_)_2_, which would be stabilized with respect to the square-planar species **E** by coordination of the free pyridyl group, given the marked ability of iridium­(I) to allow five-coordinate species. Accordingly, after 24 h, the ^31^P­{^1^H} NMR spectrum of the reaction solution contains a singlet at 36.3 ppm that, unlike the signals corresponding to **7** and **7d**, does not split under off-resonance conditions (Figure S30). There are precedents for similar transformations; for example, the osmium­(II) complex OsH­{κ^3^-*N,N′,C*-[Mepz-py-C_6_H_4_]}­(P^i^Pr_3_)_2_ is unstable in solution and evolves toward its osmium­(IV) isomer OsH_2_{κ^3^-*C*,*N*,*C′*-[C_3_NNMe-py-C_6_H_4_]}­(P^i^Pr_3_)_2_ via rollover-type CH activation of the pyrazolyl group.^27^

### Photophysical and Electrochemical Properties of **2–7**

To know the capacity of the prepared complexes to act as phosphorescent emitters with application as photocatalysts, their absorption and emission spectroscopic characteristics, as well as the electrochemical properties, were studied.

Table 3 shows the most notable absorptions of **2**–**7**. The UV–vis spectra of these compounds, in 2-methyltetrahydrofuran (2-MeTHF), at room temperature contain bands with intensities that depend on their position (Figures S43–S48). Very strong absorptions (ε ≈ 69,000–10,000 M^–1^ cm^–1^) are located below 300 nm, strong bands (ε ≈ 27,000–5000 M^–1^ cm^–1^) emerge in the region of 300–430 nm (330–500 nm for **2**), while weak absorptions (ε < 3000 M^–1^ cm^–1^) appear at energies lower than 430 nm (510 nm for **2**). The spectra were calculated in tetrahydrofuran by TD-DFT (B3LYP-D3//SDD­(f)/6-31G**); Figures S43–S48 give a view of those obtained, while Tables S1–S6 collect the transitions that contribute to the different bands. Figures S49–S54 show the most relevant orbitals, whereas Tables S7–S12 list the fragments involved in said orbitals. The higher energy bands are mainly due to intrapincer ^1^π–π* transitions. The absorptions observed in the intermediate energy region correspond to spin-allowed charge transfers from the iridium center to the pincer combined with intrapincer transitions. Lower energy tails imply formally spin-forbidden HOMO–LUMO, combined with HOMO–1–LUMO or HOMO–LUMO + 1, transitions, which result from the large spin–orbit coupling provided by iridium. The HOMO of all of them is distributed between the metal (17–37%) and one of the activated groups (40–73%). For its part, LUMO covers the 2e donor group (60–80%) and the other activated group (17–31%), except for complex **6**, which has it distributed in a similar way between the three groups of the pincer ligand.

**Table 3:** Selected UV–Vis Absorptions Recorded in 2-MeTHF and Computed TD-DFT Vertical Excitation Energies (in THF)

λ exp (nm)	ε (M^–^^1^ cm^–^^1^)	excitation energy (nm)	oscillator strength, *f*	transition	character of the transition
Complex **2**					
291	24,000	284	0.0348	HOMO–7 → LUMO (76%)	(L → L)
429	9000	443	0.0659	HOMO–1 → LUMO (74%)	(Ir + L → L)
517	300	509	0	HOMO → LUMO (91%)	(Ir + L → L)
Complex **3**					
260	52,000	274	0.0253	HOMO–5 → LUMO (91%)	(L → L)
330	27,300	326	0.2957	HOMO–3 → LUMO (68%)	(Ir + L → L)
370	17,400	377	0.0377	HOMO → LUMO + 1 (80%)	(Ir + L → L)
430	2500	433	0	HOMO–1 → LUMO (52%)	(Ir + L → L)
				HOMO → LUMO + 1 (25%)	
Complex **4**					
240	68,100	270	0.0513	HOMO–5 → LUMO (85%)	(L → L)
340	18,900	323	0.2495	HOMO–3 → LUMO + 1 (49%)	(Ir + L → L)
				HOMO–3 → LUMO (37%)	
396	16,700	383	0.1974	HOMO–1 → LUMO (85%)	(Ir + L → L)
432	2900	442	0	HOMO–1 → LUMO (55%)	(Ir + L → L)
				HOMO → LUMO (25%)	
Complex **5**					
278	16,690	274	0.0545	HOMO–4 → LUMO + 1 (70%)	(L → L)
322	9120	313	0.1077	HOMO–3 → LUMO + 1 (84%)	(Ir + L → L)
366	6720	366	0.0819	HOMO–1 → LUMO (67%)	(Ir + L → L)
480	330	491	0	HOMO → LUMO (15%)	(Ir + L → L)
				HOMO → LUMO + 1 (67%)	
Complex **6**					
238	38,584	230	0.2884	HOMO–4 → LUMO (53%)	(L → L)
339	6957	320	0.0577	HOMO → LUMO + 1 (95%)	(Ir + L → L)
362	5768	340	0.0382	HOMO → LUMO (95%)	(Ir + L → L)
387	1465	401	0	HOMO → LUMO (65%)	(Ir + L → L)
				HOMO → LUMO + 1 (25%)	
Complex **7**					
300	10,910	299	0.1822	HOMO–4 → LUMO (66%)	(L → L)
370	4400	383	0.0818	HOMO–1 → LUMO (73%)	(Ir + L → L)
408	5740	402	0.1429	HOMO → LUMO (85%)	(Ir + L → L)
490	380	472	0	HOMO → LUMO (65%)	(Ir + L → L)

The HOMO–LUMO gap is between 4.35 and 3.25 eV, decreasing in the sequence **6** > **3** > **7** > **5** > **4** > **2**. For the six complexes, the energy calculated for the HOMO and that estimated from the potentials versus Fc^+^/Fc of the first oxidation are in excellent agreement (Table 4). In this context, it is worth highlighting the depth of the HOMO of complex **3**. Its energy level of −5.20 eV is between 0.4 and 0.5 eV below the rest. Figure S55 represents the voltammograms, which were measured in dichloromethane, under argon, using [Bu_4_N]­PF_6_ as the supporting electrolyte. Complexes **2** and **4** present three oxidations (Ir­(III)/Ir­(IV), Ir­(IV)/Ir­(V), and Ir­(V)/Ir­(VI)) between −0.10 and 1.03 V, while for **3** and **5**–**7**, only two (Ir­(III)/Ir­(IV) and Ir­(IV)/Ir­(V)) are observed between −0.06 and 0.88 V. They are reversible except for the second one of **2** and the first of **7**, which appear at 0.20 and 0.03 V, respectively.

**Table 4:** Electrochemical and DFT MO Energy Data for Complexes **2**–**7**

complex	*E*_1/2_^ox^ ^a^ (V)	obs (eV)	calcd (eV)		
		HOMO^b^	HOMO^c^	LUMO^c^	HLG^c^,^d^
**2**	–0.10, 0.20^e^, 0.84	–4.70	–4.92	–1.67	3.25
**3**	0.40, 0.88	–5.20	–5.01	–1.24	3.75
**4**	–0.01, 0.21, 1.03	–4.79	–4.64	–1.10	3.54
**5**	0.05, 0.59	–4.85	–4.66	–1.07	3.59
**6**	–0.06, 0.21	–4.74	–4.56	–0.21	4.35
**7**	0.03^e^, 0.40	–4.83	–4.82	–1.14	3.68

a Measured in CH_2_Cl_2_ (10^–3^ M)/[Bu_4_N]­PF_6_ (0.1 M) solutions, under argon, vs Fc^+^/Fc at 0.1 V/s, at room temperature.

b HOMO = −[*E*_1/2_^ox^ vs Fc^+^/Fc + 4.8] eV.

c Values from electronic structure DFT calculations.

d HGL = LUMO – HOMO.

e Anodic potential (irreversible oxidation).

Complexes **3** and **5** are efficient green emitters (498–572 nm). On the contrary, compounds **2**, **4**, and **7** are very low emissive in the red, orange, and green regions, respectively, and the NHC derivative **6** is not emissive. The measurements were carried out by photoexcitation, in three different conditions: in a poly­(methyl methacrylate) film (PMMA) doped with 5% by weight of the corresponding compound, at room temperature, and in 2-MeTHF at room temperature and 77 K. Table 5 lists the most notable findings. Figure 6 shows the spectra of **3** and **5** and Figures S57–S72 those of **2**, **4**, and **7**. The emissions originate from the respective T_1_ states, as deduced from the excellent agreement between the wavelengths of the maxima, in 2-MeTHF, at room temperature and the values calculated for the energy differences between the optimized states T_1_ and S_0_, in tetrahydrofuran.

**Table 5:** Emission Data for **2**–**5** and **7**

calcd λ_em_ (nm)	media (*T*/K)	λ_em_ (nm)	fwhm^a^ (nm)	τ^b^ (μs)	Φ	*k*_r_ ^c^ (s^–1^)	*k*_nr_ ^c^ (s^–1^)	*k*_r_/*k*_nr_
Complex **2**								
	PMMA (298)	632, 679, 731	155	3.6 (0.4, 7.7%; 3.9, 92.3%)	0.07	1.9 × 10^4^	2.6 × 10^5^	0.08
657	MeTHF (298)	634, 682	130	4.8	0.04	8.3 × 10^3^	2.0 × 10^5^	0.04
	MeTHF (77)	620, 635, 676, 736		8.9 (9.7, 82.1%; 5.3, 17.9%)				
Complex **3**								
	PMMA (298)	530	86	11.3 (13.0, 70.4%; 7.5, 29.6%)	0.9	8.0 × 10^4^	8.9 × 10^3^	9
527	MeTHF (298)	530	83	17.3	0.67	3.9 × 10^4^	1.9 × 10^4^	2.03
	MeTHF (77)	498, 528, 572		21.3 (24.4, 65.9%; 15.4, 34.1%)				
Complex **4**								
	PMMA (298)	568, 612	102	13.4 (21.9, 32.3%; 9.3, 67.7%)	0.15	1.1 × 10^4^	6.3 × 10^4^	0.18
597	MeTHF (298)	576, 624, 680	98	36.5	0.09	2.5 × 10^3^	2.5 × 10^4^	0.1
	MeTHF (77)	552, 602, 652		95				
Complex **5**								
	PMMA (298)	534	88	5.9 (0.5, 2.1%; 6.0, 97.9%)	0.66	1.1 × 10^5^	5.8 × 10^4^	1.94
554	MeTHF (298)	539	87	9.6	0.65	6.8 × 10^4^	3.7 × 10^4^	1.86
	MeTHF (77)	470, 507, 542		13.6				
Complex **7**								
	PMMA (298)	540	90	2.0 (0.4, 18.7%; 2.4, 81.3%)	0.03	1.5 × 10^4^	4.9 × 10^5^	0.03
523	MeTHF (298)	535	96	2.0 (4.0, 18.8%; 1.9, 81.2%)	0.03	1.0 × 10^4^	3.3 × 10^5^	0.03
	MeTHF (77)	487, 501, 524		4.1 (5.1, 68.0%; 2.0, 32.0%)				

a Full width at half-maximum.

b Relative amplitudes (%) are given in parentheses for biexponential decays.

c Calculated according to *k*_r_ = Φ/τ and *k*_nr_ = (1 – Φ)/τ. For biexponential decays, the amplitude-weighted average lifetimes were used.

**Figure 6:**
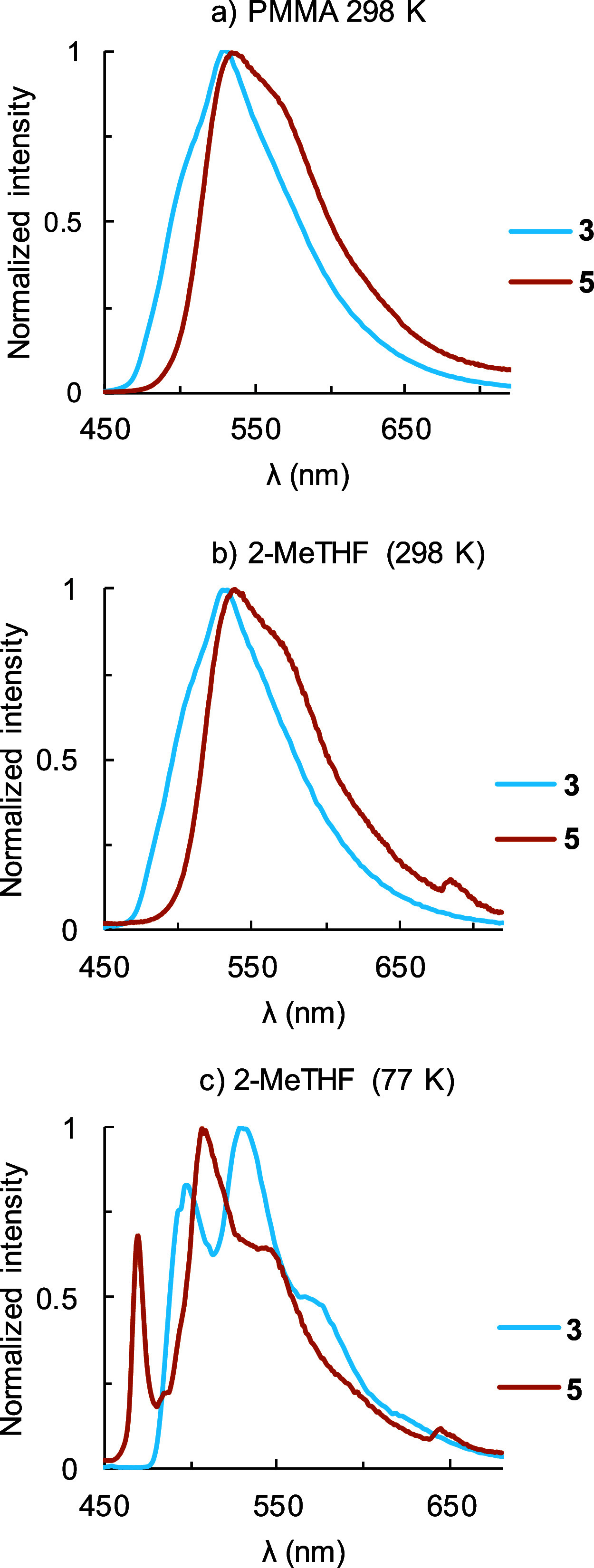
Emission spectra of **3** and **5** in (a) 5 wt % PMMA films at 298 K, (b) 2-MeTHF at 298 K, and (c) 2-MeTHF at 77 K.

The spectra of the efficient green emitters **3** and **5** are similar. They show structureless bands at room temperature with fwhm values in the range of 83–88 nm in both PMMA and 2-MeTHF. However, these bands split into the typical vibronic fine structures observed in 2-MeTHF at 77 K. This confirms a relevant contribution of an intraligand π–π* component to the emission, involving the pincer. The lifetime values of **3** are relatively long, ranging from 11.3 to 21.3 μs, and about twice as long as the values of **5**; 5.9–13.6 μs. The quantum yield of **3** in 2-MeTHF is high at 0.67 and increases to 0.90 in PMMA film. The decrease in quantum yield in solution seems to be associated with a nonradiative decay of the excited state. In accordance with this, a significant reduction of the nonradiative rate constant (*k*_nr_) is observed in the PMMA film with respect to the 2-MeTHF solution (8.9 × 10^3^ s^–1^ versus 1.9 × 10^4^ s^–1^); the value of the radiation rate constant (*k*_r_) in the PMMA film is also twice that in the 2-MeTHF solution. The quantum yields of **5** in PMMA and 2-MeTHF are high and almost identical: 0.66 and 0.65.

Low quantum yields have been associated with the existence of thermally accessible triplet excited states, centered on the metal, that provide nonradiative decay pathways.^9f,28^ However, this does not appear to be the case for **2**, **4**, and **7** since there are no significant differences between the spin density distribution calculated for the optimized triplet T_1_ of these compounds and those of **3** and **5**; in all cases, it is mainly centered on the pincer (Figure 7). On the contrary, it is observed that the nonradiative rate constant is 1 or 2 orders of magnitude greater than the radiative rate constant, for the three emitters, both in PMMA film and in 2-MeTHF solution.

**Figure 7:**
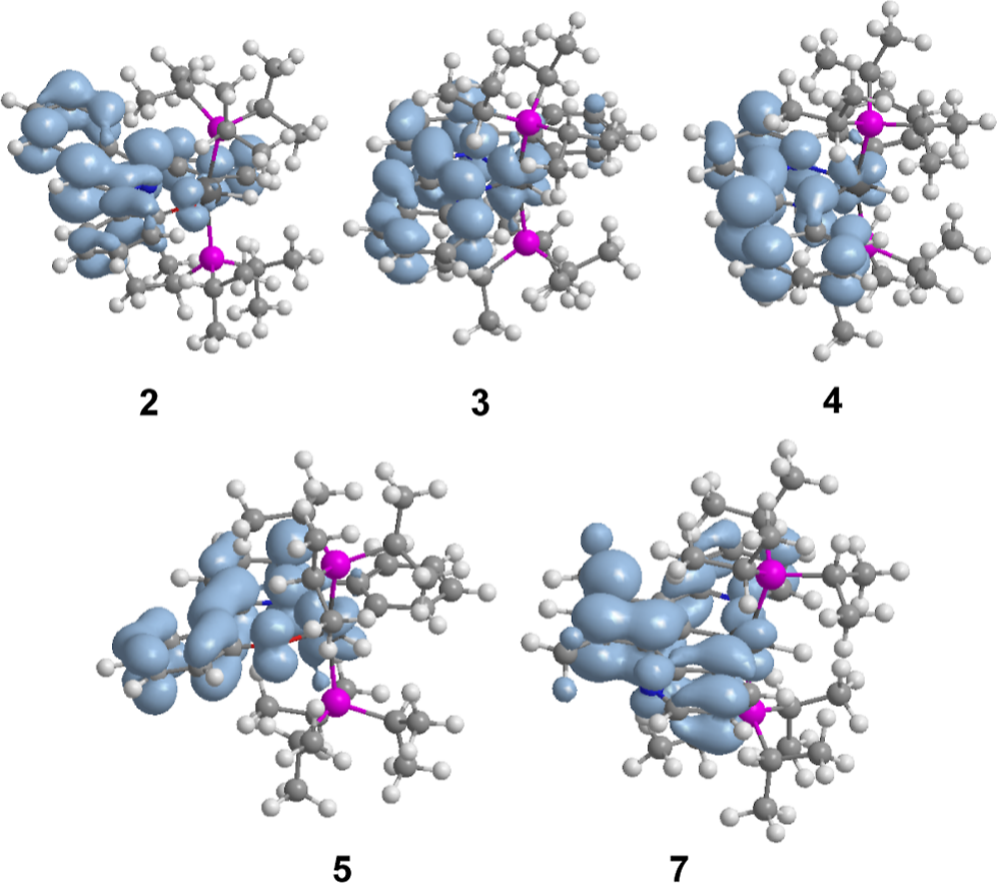
Spin density distributions for the optimized triplet T_1_ of complexes **2**–**5** and **7** (0.002 isovalue).

### Photocatalytic Performance of **3** and **5** in the α-Amino C­(sp^3^)–H Arylation

A large majority of iridium­(III) complexes that have photocatalytic applications are bis- or tris-cyclometalated species;^13^ iridium­(III) photocatalysts containing three monodentate ligands, including IrH­(PR_3_)_2_ moieties, are unknown. However, complexes **3** and **5**, especially **3**, exhibit characteristics consistent with notable capacity as photocatalysts.^29^ Both complexes have good absorption in a wide range of wavelengths. Its emission also achieves a high quantum yield in solution and therefore the formation yield of the respective reactive excited state should be high; approximately 70%. Furthermore, said excited state would persist long enough to react with the substrate since they have a sufficiently long lifetime (10–17 μs). Complexes **3** and **5** exhibit reversible electrochemical behavior. In this context, it should be mentioned that the *E*_1/2_(Ir^4+^/Ir^3+^) value *versu*s Fc^+^/Fc for **3** (0.40 V) is similar to that for Ir­{κ^2^-*C,N*-[2-C_6_H_4_-py]}_3_ (0.36 V);^30^ a prototypical photocatalyst for the α-amino C­(sp^3^)–H arylation with cyano derivatives.^14,15^

The potentials *E*_1/2_(Ir^4+^/^T1^Ir^3+^) of the excited state of a iridium­(III) photocatalyst can be determined using eq 1, where *E*_1/2_(Ir^4+^/Ir^3+^) is the reduction potential for the oxidation of Ir^3+^ to Ir^4+^ and *E*_0‑0_ is the free energy stored in the excited state.^31^ The value of *E*_0‑0_ has been obtained in different ways.^11a^ Given the observed divergence of values, we decided to estimate those of **3** and **5** using several methods, including: DFT calculations considering a typical solvent for α-amino C­(sp^3^)–H arylation reactions as *N*,*N*-dimethylacetamide, from the interception of the absorption and emission spectra in 2-MeTHF at room temperature and 77 K, and from the emission maximum in the spectra in 2-MeTHF at room temperature and 77 K. By using eq 1, the *E*_0‑0_ values obtained from these methods yield *E*_1/2_(Ir^4+^/^T1^Ir^3+^) potentials in the range between −2.24 and −1.88 V for **3** and −2.66 and −2.11 V for **5** (Table 6).

1 $$E_{1/2}\left({Ir}^{4+}{/}^{T1}{Ir}^{3+}\right)=E_{1/2}\left({Ir}^{4+}/{Ir}^{3+}\right)-E_{0‐0}$$

**Table 6:** Electrochemical Data for the Photocatalytic Reactions

compound	*E*_1/2_ (Ir^4+^/Ir^3+^)^a^ (V)	*E*_0__‑__0_ (eV)	*E*_1/2_ (Ir^4+^/^T1^Ir^3+^)^b^ (V)	*E*_1/2_ (NCb/NCb^–^)^c^ (V)	*E*_p/2_ (am^+^/am)^d^ (V)	Δ*E*_b_ ^e^ (V)		Δ*E*_c_ ^f^ (V)	
						1,4-DCB	4-NCpy	PhNMe_2_	phpiperidine
**3**	0.4	2.28^g^	–1.88			0.39	0.32	0.1	0.1
		2.68^h^	–2.28			0.79	0.72		
		2.64^i^	–2.24			0.75	0.68		
		2.34^j^	–1.94			0.45	0.38		
		2.49^k^	–2.09			0.6	0.53		
**5**	0.05	2.16^g^	–2.11			0.62	0.55	–0.25	–0.25
		2.57^h^	–2.52			1.03	0.96		
		2.71^i^	–2.66			1.17	1.1		
		2.32^j^	–2.27			0.78	0.71		
		2.64^k^	–2.59			1.1	1.03		
1,4-DCB				–1.49					
4-NCpy				–1.56					
PhNMe_2_					0.3				
phpiperidine					0.3				

a Values from Table 4.

b Estimated from eq 1.

c *E*_1/2_ in CH_2_Cl_2_ vs Fc^+/^Fc.

d Half-peak potential in CH_2_Cl_2_ vs Fc^+/^Fc.

e According to eq 2.

f According to eq 3.

g Estimated from DFT calculations in *N*,*N*-dimethylacetamide as the singlet-triplet energy gap (Δ*G*_ST_).

h Estimated from the intercept of the absorption and emission spectra in 2-MeTHF at rt.

i Estimated from the intercept of the absorption and emission spectra in MeTHF at 77 K.

j Estimated from λ_max_ in MeTHF at rt.

k Estimated from λ_max_ in MeTHF at 77 K.

The values in both ranges are more negative than the reduction potential reported for the reduction of 1,4-dicyanobenzene (1,4-DCB), −1.20 V versus Fc^+^/Fc. To confirm this, we studied the electrochemical properties of 1,4-dicyanobenzene and 4-cyanopyridine (4-NCpy) under the conditions previously used to obtain the potentials collected in Table 4. In our hands, the reversible reduction of these cyano derivatives (NCb) to the corresponding anionic radicals provides reduction potentials (*E*_1/2_(NCb/NCb^–^)) of −1.49 and −1.56 V versus Fc^+^/Fc, respectively (Table 6), which are in fact less negative than those obtained for the excited state oxidation of **3** and **5** to the respective iridium­(IV) species. Therefore, the redox reaction *b* of the cycle shown in Scheme 1 is a possible thermodynamic process (Δ*E*_b_ > 0) for both cyano derivatives and both emitters, according to eq 2.

2 $$\Delta E_{b}=E_{1/2}\left(NCb/{NCb}^{-}\right)-E_{1/2}\left({Ir}^{4+}{/}^{T1}{Ir}^{3+}\right)$$

Once the viability for the reduction of the cyano derivatives to the corresponding anionic radicals was established, we selected dimethylphenylamine and *N*-phenylpiperidine as model amines (am) to be arylated and studied their electrochemical features under the same conditions as previously mentioned. Both amines present an irreversible oxidation to the respective cationic amine radicals. It has been argued that peak potentials overestimated the redox potential in many cases, being the half-peak potential *E*_p/2_ an appropriate alternative. It corresponds to the potential at the half the maximum current in the cyclic voltammogram.^32^ For both amines, the value of *E*_p/2_(am^+^/am) is 0.30 V versus Fc^+^/Fc (Table 6), which is less positive than the *E*_1/2_(Ir^4+^/Ir^3+^) value for **3**, but more positive than that for **5**. Thus, according to eq 3, the oxidation of both amines, the redox reaction *c* of the cycle shown in Scheme 1, is thermodynamically possible (Δ*E*_c_ > 0) with **3**, but not with **5** (Δ*E*_c_ < 0).

3 $$\Delta E_{c}=E_{1/2}\left({Ir}^{4+}/{Ir}^{3+}\right)-E_{p/2}\left({am}^{+}/am\right)$$

Having established the ability of **3** to successfully perform the cycle shown in Scheme 1, we verified its actual photocatalytic performance for α-amino C­(sp^3^)–H arylation. We used an excess of amine because Δ*E*_c_ < Δ*E*_b_ and the amine oxidation could probably be slower than the reduction of the cyano derivative. Thus, solutions containing the cyano compound (0.25 M) and the amine (0.75 M), in *N*,*N*-dimethylacetamide, at room temperature, in the presence of 2 mol % of **3** and sodium acetate (0.5 M), were irradiated with blue LEDs (see the Experimental Section). As expected, effective formation of the desired products took place under these conditions, after 24 h. Thus, we observed quantitative arylations of both amines with 1,4-dicyanobenzene and dimethylphenylamine with 4-cyanopyridine, while the arylation of *N*-phenylpiperidine with 4-cyanopyridine required 3 mol % of the photocatalyst and the product was formed with only 60% yield. Isolated yields are given in Scheme 5.

**Scheme 5:**
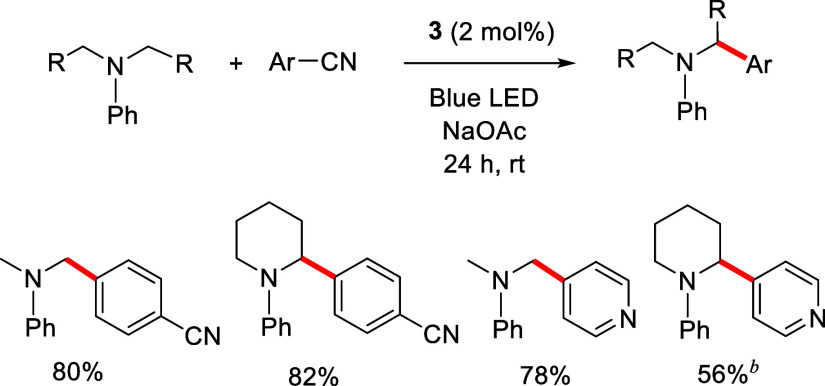
α-Amino C­(sp^3^)–H Arylation Reactions Photocatalyzed by **3** ^a^

a Reaction conditions: cyano compound (0.5 mmol), amine (1.5 mmol), **3** (7.8 mg, 0.01 mmol, 2 mol %), and NaOAc (82 mg, 1 mmol) in *N*,*N*-dimethylacetamide (2 mL) at rt.
b Three mol % of **3**.

## Concluding Remarks

This study reveals that the iridium-pentahydride complex IrH_5_(P^i^Pr_3_)_2_ activates two different σ-bonds (C–H, N–H, and O–H) of the substituents, in positions adjacent to the 2e-donor atom, of heterocycles such as isoquinoline, pyridine, and imidazolylidene to give IrH­(κ^3^-L)­(P^i^Pr_3_)_2_ complexes with five different classes of L-pincer ligands: *C,N,C*′; *N,N,*′*C*; *C,N,O*; *C,C*′*O*; and *N,C,C*′. The activations are sequential, with the second generally being the slowest. Accordingly, dihydride intermediates have been detected and characterized spectroscopically. Complexes **3** and **5** prepared in this way are green phosphorescent emitters upon photoexcitation, exhibiting characteristics consistent with notable ability as photocatalysts, including: good absorption over a wide range of wavelengths, emission achieving high quantum yields in solution (about 0.70), long enough lifetimes (10–17 μs), and reversible electrochemical behavior. Accordingly, complex **3** is an unusual example of a hydride-metal-phosphine photocatalyst. It is effective for the α-amino C­(sp^3^)–H arylation with aromatic cyano derivatives, under blue light irradiation.

We can conclude that the ability of platinum group metal polyhydride complexes to promote σ-bond activation reactions can be employed to prepare new classes of photocatalysts for organic reactions, including the formation of C–C bonds, in what represents a new evidence of the actual usefulness of polyhydride derivatives.

## Experimental Section

General information and instrumental methods used for characterization, X-ray information, and computational details are given in the Supporting Information. Chemical shifts (in ppm) are referenced to residual solvent peaks (^1^H, ^13^C­{^1^H}) and external H_3_PO_4_ (^31^P­{^1^H}). Coupling constants, *J*, and *N* (*N* = ^3^ *J*_H–P_ + ^5^ *J*_H–P′_ for ^1^H or ^1^ *J*_C–P_ + ^3^ *J*_C–P′_ for ^13^C) are given in Hertz. Complex IrH_5_(P^i^Pr_3_)_2_ (**1**) was prepared according to the published method.^3v^

### Preparation of IrH­{κ^3^-*C,N,C*-(C_6_H_4_-isoqui-OC_6_H_4_)}­(P^i^Pr_3_)_2_ (**2**)

A solution of **1** (300 mg, 0.58 mmol) and 3-phenoxy-1-phenylisoquinoline (207 mg, 0.70 mmol) in toluene (15 mL) was refluxed for a week. The solvent was then evaporated. The crude product was purified by column chromatography in deactivated silica gel using a pentane/dichloromethane mixture (4:1) as eluent, collecting an orange fraction. The solvent was evaporated by vacuum, giving an orange powder. Yield: 148 mg (32%). Anal. Calcd for C_39_H_56_IrNOP_2_: C, 57.90; H, 6.98; N, 1.73. Found: C, 58.27; H, 6.86; N, 1.89. HRMS (electrospray, *m*/*z*): calcd for C_39_H_56_IrNOP_2_ [M]^+^: 809.3461; found, 809.3463. IR (cm^–1^): *v*(IrH) 2194 (w). ^1^H NMR (300 MHz, C_6_D_6_, 298 K): δ 8.86 (d, ^3^ *J*_H–H_ = 8.9, 1H, CH arom), 8.43–8.36 (2H, CH arom), 8.17, (d, ^3^ *J*_H–H_ = 8.2, 1H, CH arom), 7.43 (dd, ^3^ *J*_H–H_ = 7.9, ^4^ *J*_H–H_ = 1.4, 1H, CH arom), 7.37 (d, ^3^ *J*_H–H_ = 8.0, 1H, CH arom), 7.17–7.04 (6H, CH arom), 6.95 (ddd, ^3^ *J*_H–H_ = 7.2, ^3^ *J*_H–H_ = 7.2, ^4^ *J*_H–H_ = 1.4, 1H, CH arom), 1.85 (m, 6H, PCH), 0.96 (dvt, *N* = 13.2, ^3^ *J*_H–H_ = 6.4, 18H, PCHC*H*_3_), 0.71 (dvt, *N* = 13.0, ^3^ *J*_H–H_ = 6.5, 18H, PCHC*H*_3_), −18.37 (t, ^2^ *J*_H–P_ = 19.5, 1H, IrH). ^31^P­{^1^H}­NMR: (121.5 MHz, C_6_D_6_, 298 K): δ 2.3 (s, d under off-resonance conditions). ^13^C­{^1^H}­NMR (75.5 MHz, C_6_D_6_, 298 K): δ 171.8 (t, ^2^ *J*_C–P_ = 8.8, IrC), 171.4, 157.0, 154.3 (all s, C_q_), 149.7 (s, CH arom), 148.3 (s, C_q_), 144.2 (s, CH arom), 140.3 (s, C_q_), 131.8 (s CH arom), 131.0 (t, ^2^ *J*_C–P_ = 9.4, IrC), 129.7, 126.7, 124.8 (all s, CH arom), 123.6 (s, C_q_), 122.9, 122.7, 120.0, 115.6, 103.6 (s, CH arom), 25.9 (vt, *N* = 26.6, P*C*H), 19.9, 19.3 (both s, PCH*C*H_3_).

### Reaction of 1 with 3-Phenoxy-1-phenylisoquinoline: Spectroscopic Detection of IrH_2_{κ^2^-*C,N*,-(C_6_H_4_-isoqui-OC_6_H_5_)}­(P^i^Pr_3_)_2_ (**2d**)

A solution of **1** (300 mg, 0.58 mmol) and 3-phenoxy-1-phenylisoquinoline (207 mg, 0.70 mmol) in toluene (15 mL) was refluxed during 24 h. After that time, NMR spectra of the crude in C_6_D_6_ showed the presence of monohydride **2**, dihydride **2d**, and **1** in a 15:54:31 molar ratio. Small amount of crystals of **2d** suitable for X-ray diffraction analysis were grown in a concentrated solution of the mixture in pentane at −18 °C. NMR data for **2d**: ^1^H NMR (300 MHz, C_6_D_6_, 298 K): δ 8.85 (d, ^3^ *J*_H–H_ = 7.9, 1H, CH arom), 8.67 (d, ^3^ *J*_H–H_ = 6.9, 1H, CH arom), 8.34 (dd, ^3^ *J*_H–H_ = 7.3, ^4^ *J*_H–H_ = 1.8, 1H, CH arom), 7.43 (dd, ^3^ *J*_H–H_ = 7.9, ^4^ *J*_H–H_ = 1.4, 1H, CH arom), 7.37 (d, ^3^ *J*_H–H_ = 7.7, 1H, CH arom), 7.30 (d, ^3^ *J*_H–H_ = 8.3, 1H, CH arom), 7.28–7.04 (8H, CH arom), 2.04 (m, 6H, PCH), 1.05 (dvt, *N* = 13.4, ^3^ *J*_H–H_ = 7.0, 18H, PCHC*H*_3_), 1.01 (dvt, *N* = 12.7, ^3^ *J*_H–H_ = 6.9, 18H, PCHC*H*_3_), −12.55 (tdd, ^2^ *J*_H–P_ = 20.6, ^2^ *J*_H–H_ = 5.4, ^4^ *J*_H–H_ = 1.4 1H, IrH), −21.82 (td, ^2^ *J*_H–P_ = 18.3, ^2^ *J*_H–H_ = 5.4, 1H, IrH). ^31^P­{^1^H}­NMR: (121.5 MHz, C_6_D_6_, 298 K): δ 30.3 (s, t under off-resonance conditions). ^13^C­{^1^H}­NMR (75.5 MHz, C_6_D_6_, 298 K): δ 185.0 (t, ^2^ *J*_C–P_ = 6.8, IrC), 174.2, 161.7, 155.4, 149.4 (all s, C_q_), 144.9 (s, CH arom), 139.6 (s, C_q_), 130.6, 130.5, 129.9, 128.8, 127.94, 126.8, 125.4, 124.0 (all s, CH arom), 123.7 (s, C_q_), 121.5, 120.7, 104.6 (all s, CH arom), 26.7 (vt, *N* = 27.1, P*C*H), 20.4, 19.9 (both s, PCH*C*H_3_).

### Preparation of IrH­{κ^3^-*N,N,C*-[NBzim-py-C_6_H_4_]}­(P^i^Pr_3_)_2_ (**3**)

A colorless solution of **1** (100 mg, 0.193 mmol) in 10 mL of toluene was treated with 2-(1*H*-benzimidazol-2-yl)-6-phenylpyridine (HNbzim-py-C_6_H_5_) (52 mg, 0.193 mmol) and heated under reflux for 24 h. The solvent was removed under vacuum and methanol was added to afford a yellow solid, which was washed with methanol at −78 °C and vacuum-dried. Yield: 104 mg (69%). Alternatively, the reaction can be carried out in refluxing *p*-xylene for 5 h. Crystals suitable for X-ray diffraction analysis were grown in a benzene-*d*_6_ solution at room temperature. Anal. Calcd for C_36_H_55_IrN_3_P_2_: C, 55.15; H, 7.07; N, 5.36. Found: C, 55.01; H, 7.14; N, 5.26. HRMS (electrospray, *m*/*z*): calcd for C_36_H_55_IrN_3_P_2_ [M]^+^ 784.3497; found, 784.3507. IR (cm^–1^): *v*(IrH) 2168 (w). ^1^H NMR (300 MHz, C_6_D_6_, 298 K): δ 8.39 (dd, ^3^ *J*_H–H_ = 7.3, ^4^ *J*_H–H_ = 1.5, 1H, H py), 8.17 (m, 1H, C_6_H_4_), 7.75–7.62 (2H arom), 7.43 (dd, ^3^ *J*_H–H_ = 7.1, ^4^ *J*_H–F_ = 2.2, 1H bzim), 7.30–7.07 (4H arom), 7.01–6.89 (2H arom), 1.69 (m, 6H, PCH), 0.70 (dvt, *N* = 13.1, ^3^ *J*_H–H_ = 6.5, 18H, PCHC*H*_3_), 0.64 (dvt, *N* = 13.37, ^3^ *J*_H–H_ = 6.7, 18H, PCHC*H*_3_), −14.86 (t, ^2^ *J*_H–P_ = 19.4, 1H, IrH). ^31^P­{^1^H} NMR (121.42 MHz, C_6_D_6_, 298 K): δ 5.0 (s, d under off-resonance conditions). ^13^C­{^1^H} NMR (75.42 MHz, C_6_D_6_): δ 165.9 (s, C_q_ py), 164.9 (s, NC_q_N), 155.7 (s, C_q_ py), 148.4 (s, C_q_ C_6_H_4_), 148.1 (s, C_q_ bzim), 147.6 (s, C_q_ bzim), 146.3 (t, ^2^ *J*_C–P_ = 6.4, IrC), 141.0 (s, CH arom), 137.0 (s, CH py), 129.7, 124.4, 121.1, 121.0, 120.9, 120.7 (all s, CH arom), 116.9 (s, CH py), 115.7 (s, CH arom), 114.7 (s, CH py), 25.3 (vt, *N* = 26.7 Hz, PCH), 19.3, 18.9 (both s, PCH*C*H_3_).

### Reaction of 1 with 2-(1*H*-Benzimidazol-2-yl)-6-phenylpyridine: Spectroscopic Detection of IrH_2_{κ^2^-*N,N*-[NBzim-py-C_6_H_5_]}­(P^i^Pr_3_)_2_ (**3d**)

A colorless solution of **1** (100 mg, 0.193 mmol) in 10 mL of toluene was treated with 2-(1*H*-benzimidazol-2-yl)-6-phenylpyridine (52 mg, 0.193 mmol) and heated under reflux. After 10 h, the ^31^P­{^1^H} NMR spectrum of an aliquot of the reaction crude showed the presence of a mixture containing **3d** and **3**, in a molar ratio of 80:20. NMR Data for **3d**: ^1^H NMR (300 MHz, C_6_D_6_, 298 K): δ 9.07 (dd, ^3^ *J*_H–H_ = 7.9, ^4^ *J*_H–H_ = 1.6, 1H, py), 8.31 (m, 1H, bzim), 7.78 (m, 1H, bzim), 7.70 (m, 2H Ph), 7.35 (m, 2H, bzim), 7.23–7.14 (m, 3H Ph), 7.11 (dd, ^3^ *J*_H–H_ = 7.9, ^3^ *J*_H–H_ = 7.5, 1H py), 6.80 (dd, ^3^ *J*_H–H_ = 7.5, ^4^ *J*_H–H_ = 1.6, 1H bzim), 1.98 (m, 6H, PCH), 0.86 (dvt, *N* = 13.2, ^3^ *J*_H–H_ = 6.6, 18H, PCHC*H*_3_), 0.60 (dvt, *N* = 12.9, ^3^ *J*_H–H_ = 6.6, 18H, PCHC*H*_3_), −20.35 (td, ^2^ *J*_H–P_ = 17.1, ^2^ *J*_H–H_ = 7.0, 1H, IrH), −23.18 (td, ^2^ *J*_H–P_ = 18.4, ^2^ *J*_H–H_ = 7.0, 1H, IrH). ^31^P­{^1^H} NMR (121.42 MHz, C_6_D_6_, 298 K): δ 20.7 (s, t under off-resonance conditions). ^13^C­{^1^H} NMR (75.42 MHz, C_6_D_6_): δ 163.3 (s, NC_q_N bzim), 162.5 (s, C_q_ py), 158.8 (s, C_q_ py), 148.6 (s, C_q_ py), 146.6 (s, C_q_ py), 143.1 (s, C_q_ Ph), 136.4 (s, CH py), 130.8 (s, 2CH Ph), 128.5 (s, CH Ph), 127.0 (s, 2CH Ph), 124.3 (s, CH py), 122.4 (s, CH py), 120.9 (s, CH bzim), 120.7 (s, CH bzim), 120.6 (s, CH bzim), 116.5 (s, CH bzim), 27.1 (vt, *N* = 26.2 Hz, PCH), 19.8, 18.9 (both s, PCH*C*H_3_).

### Preparation of IrH­{κ^3^-*N,N,C*-[Ind-py-C_6_H_4_]}­(P^i^Pr_3_)_2_ (**4**)

A colorless solution of **1** (200 mg, 0.386 mmol) in 10 mL of toluene was treated with 2-(1*H*-indol-2-yl)-6-phenylpyridine (104 mg, 0.386 mmol) and heated under reflux. After 24 h, the solvent was removed under vacuum and methanol was added to afford a yellow solid, which was washed with methanol at −78 °C and vacuum-dried. Yield: 250 mg (77%). Anal. Calcd for C_37_H_56_IrN_2_P_2_: C, 56.75; H, 7.21; N, 3.58. Found: C, 56.65; H, 7.05; N, 3.7. HRMS (electrospray, *m*/*z*): calcd for C_37_H_56_IrN_2_P_2_ [M]^+^ 783.3543; found, 783.3517. IR (cm^–1^): *v*(IrH) 2154 (w). ^1^H NMR (300 MHz, C_6_D_6_, 298 K): δ 7.97 (d, ^3^ *J*_H–H_ = 7.2, 1H, H arom), 7.87 (m, 1H arom), 7.72 (d, m, 1H arom), 7.57 (m, 1H arom), 7.43 (dd, ^3^ *J*_H–H_ = 7.4, ^4^ *J*_H–H_ = 1.1, 1H arom), 7.37 (m, 1H arom), 7.34 (m, 1H arom), 7.25–7.14 (3H arom), 7.09–7.00 (2H arom), 1.81 (m, 6H, PCH), 0.82 (dvt, *N* = 13.2, ^3^ *J*_H–H_ = 6.5, 18H, PCHC*H*_3_), 0.77 (dvt, *N* = 13.3, ^3^ *J*_H–H_ = 6.5, 18H, PCHC*H*_3_), −14.83 (t, ^2^ *J*_H–P_ = 19.7, 1H, IrH). ^31^P­{^1^H} NMR (121.42 MHz, C_6_D_6_, 298 K): δ 4.4 (s, d under off-resonance conditions). ^13^C­{^1^H} NMR (75.42 MHz, C_6_D_6_): δ 165.5 (s, C_q_), 158.8 (s, C_q_), 151.5 (s, C_q_), 149.0 (s, C_q_), 148.6 (s, C_q_), 147.4 (t, ^2^ *J*_C–P_ = 7.8, IrC^1^), 141.5, 136.0 (both s, CH arom), 131.6 (s, C_q_), 129.2, 124.4, 122.2, 120.7, 120.6, 117.7, 117.2, 113.8, 112.3, 103.5 (all s, CH Ar), 25.5 (vt, *N* = 26.3 Hz, PCH), 19.5, 19.1 (both s, PCH*C*H_3_).

### Reaction of 1 with 2-(1*H*-Indol-2-yl)-6-phenylpyridine: Spectroscopic Detection of IrH_2_{κ^2^-*N,N*-[Ind-py-C_6_H_5_]}­(P^i^Pr_3_)_2_ (**4d**)

A colorless solution of **1** (100 mg, 0.193 mmol) in 10 mL of toluene was treated with 2-(1*H*-indol-2-yl)-6-phenylpyridine (52 mg, 0.193 mmol) and heated under reflux. The ^31^P­{^1^H} NMR spectrum of an aliquot of the reaction crude showed the presence of a mixture containing **1**, **4d**, and **4** in a molar ratio of 10:42:48, after 3 h. Selected NMR data for **4d**: ^1^H NMR (300 MHz, toluene, 298 K): δ −11.59 (td, ^2^ *J*_H–P_ = 16.0, ^2^ *J*_H–H_ = 6.8, 1H, IrH), −12.64 (td, ^2^ *J*_H–P_ = 19.0, ^2^ *J*_H–H_ = 6.8, 1H, IrH). ^31^P­{^1^H} NMR (121.42 MHz, toluene, 298 K): δ 45.9 (s).

### Preparation of [IrH­{κ^3^-*C,N,O*-(C_6_H_4_-py-C_6_H_4_O)}­(P^i^Pr_3_)_2_] (**5**)

A solution of **1** (300 mg, 0.58 mmol) and 2-(2-hydroxyphenyl)-6-phenylpyridine (172 mg, 0.70 mmol) in toluene (15 mL) was refluxed for 16 h. After that time, the solvent was evaporated. The addition of 5 mL of pentane caused the appearance of a yellow solid, which was washed with pentane (2 × 3 mL) and vacuum-dried. Yield: 308 mg (70%). X-ray crystals were grown in a concentrated solution of pentane at −18 °C. Anal. Calcd for C_35_H_54_IrNOP_2_: C, 55.39; H, 7.17; N, 1.85. Found: C, 55.78; H, 7.00; N, 2.04. HRMS (electrospray, *m*/*z*): calcd for C_35_H_55_IrNOP_2_ [M + H]^+^: 760.3383; found, 760.3385. IR (cm^–1^): *v*(IrH) 2188 (w). ^1^H NMR (300 MHz, C_6_D_6_, 298 K): δ 7.81 (dd, ^3^ *J*_H–H_ = 8.2, ^4^ *J*_H–H_ = 1.7, 1H, CH OPh), 7.68 (d, ^3^ *J*_H–H_ = 7.5, 1H, CH Ph), 7.60 (d, ^3^ *J*_H–H_ = 8.3, 1H, CH OPh), 7.59 (dd, ^3^ *J*_H–H_ = 7.4, ^4^ *J*_H–H_ = 1.6, 1H, CH Ph), 7.48 (dd, ^3^ *J*_H–H_ = 8.0, ^4^ *J*_H–H_ = 1.2, 1H, CH py), 7.31 (dd, ^3^ *J*_H–H_ = 8.0; 8.0, 1H, CH py), 7.26 (ddd, ^3^ *J*_H–H_ = 8.3, ^3^ *J*_H–H_ = 6.5, ^4^ *J*_H–H_ = 1.7, 1H, CH OPh), 7.17 (dd, ^3^ *J*_H–H_ = 8.0, ^4^ *J*_H–H_ = 1.2, 1H, CH py), 7.07 (ddd, ^3^ *J*_H–H_ = 8.8, ^3^ *J*_H–H_ = 7.4, ^4^ *J*_H–H_ = 1.6, 1H, CH Ph), 7.00 (ddd, ^3^ *J*_H–H_ = 8.8, ^3^ *J*_H–H_ = 7.5, ^4^ *J*_H–H_ = 1.6, 1H, CH Ph), 6.64 (ddd, ^3^ *J*_H–H_ = 8.2, ^3^ *J*_H–H_ = 6.5, ^4^ *J*_H–H_ = 1.6, 1H, CH OPh), 2.15 (m, 6H, PCH), 0.99 (dvt, *N* = 13.1, ^3^ *J*_H–H_ = 6.9, 18H, PCHC*H*_3_), 0.87 (dvt, *N* = 12.9, ^3^ *J*_H–H_ = 6.9, 18H, PCHC*H*_3_), −16.49 (t, ^2^ *J*_H–P_ = 18.2, 1H, IrH). ^31^P­{^1^H}­NMR: (121.5 MHz, C_6_D_6_, 298 K): δ 13.9 (s). ^13^C­{^1^H}­NMR (75.5 MHz, C_6_D_6_, 298 K): δ 168.1 (s, C_q_ OPh), 166.7 (s, C_q_ py). 156.9 (s, C_q_ OPh), 146.9 (t, ^2^ *J*_C–P_ = 7.3, IrC Ph), 146.8 (s, C_q_ Ph), 141.0 (s, CH Ph), 134.7 (s CH py), 131.4, 130.7 (both s, CH OPh), 129.1 (s, CH Ph), 125.9 (s, CH py), 125.6 (s, C_q_ py), 123.7, 119.6 (both s, CH Ph), 118.0 (s, CH OPh), 114.9 (s, CH py), 113.5 (s, CH OPh), 23.8 (vt, *N* = 25.5, P*C*H), 19.0, 18.7 (both s, PCH*C*H_3_).

### Preparation of [IrH­{κ^3^-*C,C,O*-(C_6_H_4_-Im-C_6_H_4_O)}­(P^i^Pr_3_)_2_] (**6**)

To a solution of **1** (100 mg, 0.19 mmol) and *N*-(2-hydroxyphenyl)-*N*′-phenylimidazolium triflate (75 mg, 0.19 mmol), in toluene (5 mL), NEt_3_ (27 μL, 0.19 mmol) was added and the mixture was refluxed for 15 min. After that time, the solvent was evaporated. The addition of 2 mL of MeOH caused the precipitation of a white solid, which was washed with MeOH (1 × 2 mL) and vacuum-dried. Yield: 95 mg (66%). Anal. Calcd for C_33_H_53_IrN_2_OP_2_: C, 52.99; H, 7.14; N, 3.75. Found: C, 52.73; H, 7.30; N, 3.90. HRMS (electrospray, *m*/*z*): calcd for C_33_H_54_IrN_2_OP_2_ [M + H]^+^: 749.3335; found, 749.3337. IR (cm^–1^): *v*(IrH) 2014 (w). ^1^H NMR (300 MHz, C_6_D_6_, 298 K): δ 7.51 (d, ^3^ *J*_H–H_ = 7.5, 1H, CH Ph), 7.30 (d, ^3^ *J*_H–H_ = 2.1, 1H, CH im), 7.24 (dd, ^3^ *J*_H–H_ = 8.2, ^4^ *J*_H–H_ = 1.7, 1H, CH OPh), 7.21 (dd, ^3^ *J*_H–H_ = 8.3, ^4^ *J*_H–H_ = 1.7, 1H, CH OPh) 7.13 (ddd, ^3^ *J*_H–H_ = 8.2, ^3^ *J*_H–H_ = 6.8, ^4^ *J*_H–H_ = 1.7, 1H, CH OPh), 7.02 (d, ^3^ *J*_H–H_ = 2.1, 1H, CH im), 6.99–6.88 (3H, CH Ph), 6.58 (ddd, ^3^ *J*_H–H_ = 8.3, ^3^ *J*_H–H_ = 6.8, ^4^ *J*_H–H_ = 1.7, 1H, CH OPh), 2.11 (m, 6H, PCH), 1.07 (dvt, *N* = 13.3, ^3^ *J*_H–H_ = 7.0, 18H, PCHC*H*_3_), 0.85 (dvt, *N* = 13.2, ^3^ *J*_H–H_ = 7.0, 18H, PCHC*H*_3_), −8.02 (t, ^2^ *J*_H–P_ = 20.4, 1H, IrH). ^31^P­{^1^H}­NMR: (121.5 MHz, C_6_D_6_, 298 K): δ 21.9 (s). ^13^C­{^1^H}­NMR (75.5 MHz, C_6_D_6_, 298 K): δ 177.9 (t, ^2^ *J*_C–P_ = 4.5, IrC im), 158.4 (s, C_q_ OPh), 148.2 (s, C_q_ Ph), 142.0 (s. CH Ph), 129.0 (s, C_q_ OPh), 127.0, 126.0 (both s, CH OPh), 124.7 (t, ^2^ *J*_C–P_ = 7.0, IrC Ph), 124.5, 120.4 (both s, CH Ph), 117.8 (s, CH OPh), 114.8, 113.0 (both s, CH im), 111.9 (s, CH OPh), 110.2 (s, CH Ph), 23.5 (vt, *N* = 26.5, P*C*H), 18.8, 18.6 (both s, PCH*C*H_3_).

### Preparation of IrH­{κ^3^-*N,C,C*-[py-C_6_HMe_2_-C_5_H_3_N]}­(P^i^Pr_3_)_2_ (**7**)

A solution of **1** (300 mg, 0.58 mmol) and 1,3-di­(2-pyridyl)-4,6-dimethylbenzene (181 mg, 0.70 mmol) in toluene (15 mL) was refluxed for 2 days. After that time, the solvent was evaporated. The crude was purified by silica column chromatography (deactivated with NEt_3_) using pentane/dichloromethane (4:1) as eluent to eliminate an impurity and then ether to get **7** as a yellow solid. Yield: 54 mg (12%). X-ray crystals were grown in a concentrated solution of MeOH at 4 °C. Anal. Calcd for C_36_H_57_IrN_2_P_2_: C, 56.01; H, 7.44; N, 3.63. Found: C, 56.33; H, 7.32; N, 3.92. HRMS (electrospray, *m*/*z*): calcd for C_36_H_58_IrN_2_P_2_ [M + H]^+^: 773.3699; found, 73.3701. IR (cm^–1^): *v*(IrH) 1951 (w). ^1^H NMR (300 MHz, C_6_D_6_, 298 K): δ 8.86 (dd, ^3^ *J*_H–H_ = 5.7, ^4^ *J*_H–H_ = 0.9, 1H, CH py), 8.69 (dd, ^3^ *J*_H–H_ = 4.6, ^4^ *J*_H–H_ = 0.9, 1H, CH C_5_H_3_N), 7.96 (d, ^3^ *J*_H–H_ = 7.1, 1H, CH py), 7.94 (dd, ^3^ *J*_H–H_ = 7.4, ^4^ *J*_H–H_ = 0.9, 1H, CH C_5_H_3_N), 7.11 (ddd, ^3^ *J*_H–H_ = 7.1, ^3^ *J*_H–H_ = 7.1, ^4^ *J*_H–H_ = 0.9, 1H, py), 7.01 (s, 1H, C_6_ *H*(CH_3_)_2_), 6.77 (dd, ^3^ *J*_H–H_ = 7.4, ^3^ *J*_H–H_ = 4.6, 1H, CH C_5_H_3_N), 6.37 (ddd, ^3^ *J*_H–H_ = 7.1, ^3^ *J*_H–H_ = 5.7, ^4^ *J*_H–H_ = 1.3, 1H, CH py), 3.52, 2.69 (both s, 3H each, C_6_H­(C*H*_3_)_2_), 1.79 (m, 6H, PC*H*), 0.86 (m, 36H, PCHC*H*_3_), −8.17 (t, ^2^ *J*_H–P_ = 22.8, 1H, IrH). ^31^P­{^1^H}­NMR: (121.5 MHz, C_6_D_6_, 298 K): δ 8.7 (s). ^13^C­{^1^H}­NMR (75.5 MHz, C_6_D_6_, 298 K): δ 195.0 (t, ^2^ *J*_C–P_ = 4.3, IrC C_6_H­(CH_3_)_2_), 180.3 (s, C_q_ C_5_H_3_N), 172.7 (s, C_q_ py), 156.6 (s, CH py), 149.7 (s, C_q_ C_6_H­(CH_3_)_2_), 146.5, 141.3 (both s, CH C_5_H_3_N), 138.0 (t, ^2^ *J*_C–P_ = 9.4, IrC C_5_H_3_N), 135.9 (s, C_q_ C_6_H­(CH_3_)_2_), 134.6 (s, CH py), 132.4 (s, C_q_ C_6_H­(CH_3_)_2_), 130.4 (s, CH C_6_H­(CH_3_)_2_), 122.4 (s, CH py), 119.3 (s, CH C_5_H_3_N), 119.2 (s, CH py), 25.0 (vt, *N* = 26.9, P*C*H), 23.8, 21.8 (both s, C_6_H­(C*H*_3_)_2_), 18.9, 18.7 (both s, PCH*C*H_3_).

### Reaction of Complex **1** with 1,3-Di­(2-pyridyl)-4,6-dimethylbenzene: Spectroscopic Detection of IrH_2_{κ^2^-*N,C*-[py-C_6_HMe_2_-py]}­(P^i^Pr_3_)_2_ (**7d**)

A solution of **1** (100 mg, 0.19 mmol) and 1,3-di­(2-pyridyl)-4,6-dimethylbenzene (60 mg, 0.23 mmol) in toluene (5 mL) was refluxed for 24 h. After that time, the NMR spectrum of an aliquot of the reaction crude showed the presence of a mixture of monohydride **7** and dihydride **7d**, among other species. Selected NMR data for **7d**: ^1^H NMR (300 MHz, toluene, 298 K): δ −12.74 (td, ^2^ *J*_H–P_ = 21.5, ^2^ *J*_H–H_ = 4.4, 1H, IrH), −20.91 (td, ^2^ *J*_H–P_ = 18.5, ^2^ *J*_H–H_ = 4.4, 1H, IrH). ^31^P­{^1^H} NMR (121.42 MHz, toluene, 298 K): δ 21.8 (s, t under off-resonance conditions).

### Preparation of [IrH­{κ^2^-*C,C*-(C_6_H_4_-Im-C_6_H_5_)}­(P^i^Pr_3_)_2_] (**8**)

To a solution of **1** (100 mg, 0.19 mmol) and *N,N*′-diphenylimidazolium triflate (72 mg, 0.19 mmol) in toluene (5 mL), NEt_3_ (27 μL, 0.19 mmol) was added and the mixture was refluxed for 1 h. After that time, the solvent was evaporated and 2 mL of MeOH was added to precipitate a white solid, which was washed with MeOH (1 × 2 mL) and vacuum-dried. Yield: 57 mg (40%). X-ray crystals were grown in a concentrated solution of MeOH at 4 °C. Anal. Calcd for C_33_H_55_IrN_2_P_2_: C, 54.00; H, 7.55; N, 3.82. Found: C, 54.15; H, 7.73; N, 3.88. HRMS (electrospray, *m*/*z*): calcd for C_33_H_54_IrN_2_P_2_ [M – H]^+^: 733.3386; found, 733.3388. IR (cm^–1^): *v*(IrH) 2069, 2041 (w). ^1^H NMR (300 MHz, C_6_D_6_, 298 K): δ 8.36 (m, 1H, CH arom), 8.09–8.05 (2H, CH arom), 7.23–7.17 (6H, CH im + CH arom), 7.06 (m, 1H, CH arom), 6.73 (d, ^3^ *J*_H–H_ = 2.1, 1H, CH im), 1.95 (m, 6H, PCH), 1.03 (dvt, *N* = 13.4, ^3^ *J*_H–H_ = 7.0, 18H, PCHC*H*_3_), 0.85 (dvt, *N* = 12.6, ^3^ *J*_H–H_ = 7.0, 18H, PCHC*H*_3_), −14.03 (td, ^2^ *J*_H–P_ = 20.6, ^2^ *J*_H–H_ = 4.3, 1H, IrH), −14.31 (tdd, ^2^ *J*_H–P_ = 19.7, ^2^ *J*_H–H_ = 4.3, ^4^ *J*_H–H_ = 1.0, 1H, IrH). ^31^P­{^1^H}­NMR: (121.5 MHz, C_6_D_6_, 298 K): δ 28.4 (s). ^13^C­{^1^H}­NMR (75.5 MHz, C_6_D_6_, 298 K): δ 180.9 (t, ^2^ *J*_C–P_ = 6.2, IrC im), 152.0 (t, ^2^ *J*_C–P_ = 7.2, IrC C_6_H_4_), 149.0 (s, C_q_), 145.3 (s, CH arom), 141.9 (s, C_q_), 126.6, 125.2, 124.8, 120.3 (all s, CH arom), 119.2, 116.0 (both s, CH im), 110.6 (s, CH arom), 27.6 (vt, *N* = 28.4, P*C*H), 20.3, 19.4 (both s, PCH*C*H_3_).

### General Procedure for the α-Arylation of Amines

The reactions were carried out in Schlenk tubes under an argon atmosphere at room temperature. The Schlenk tube equipped with a magnetic stir bar was charged with complex **3** (0.01 or 0.015 mmol, 2 or 3 mol %), the corresponding aromatic cyano compound (0.5 mmol), sodium acetate (82 mg, 1.0 mmol; vacuum-dried at 100 °C for 12 h), 2.0 mL of *N*,*N*-dimethylacetamide, and the corresponding amine (1.5 mmol). The Schlenk was placed inside a homemade photoreactor: blue irradiation was performed with Anmossi LED strip light (2.5 m of 24 V blue (465 nm) LED strip; 40 LEDs, max. output ca. 1.2 W) strapped around a 9 cm diameter polypropylene white canister. The reactor was installed on top of a stirring plate and the top of the canister was covered with aluminum foil. After 24 h, the reaction was diluted with ethyl acetate (20 mL) and added to a separatory funnel containing 25 mL of a saturated aqueous solution of Na_2_CO_3_. The layers were separated, and the aqueous layer was extracted with EtOAc (3 × 10 mL). The combined organic extracts were washed with brine, dried (MgSO_4_), and concentrated in vacuo. Purification of the crude product by flash chromatography on silica gel using the indicated solvent system afforded the desired α-arylated amine product.

## Supplementary Material





## Notes

General information for the experimental section, NMR spectra, structural analysis, computational details and energies of optimized structures, experimental and computed UV/vis spectra, molecular orbitals, cyclic voltammograms, and normalized excitation and emission spectra (PDF)
Cartesian coordinates of the optimized structures (XYZ)
