# Supplementary material for: C–H, N–H, and O–H Bond Activations to Prepare Phosphorescent Hydride-Iridium(III)-Phosphine Emitters with Photocatalytic Achievement in C–C Coupling Reactions
Source: Inorg Chem. 2024 Mar 28;63(14):6346–61. doi: 10.1021/acs.inorgchem.4c00115 (PMC12582129; doi:10.1021/acs.inorgchem.4c00115)
Supplement: Supplementary file 1 [file ic4c00115_si_001.pdf]

Supporting Information For

C–H, N–H, and O–H Bond Activations to Prepare Phosphorescent Hydride-Iridium(III)-Phosphine Emitters with Photocatalytic Achievement in C–C Coupling Reactions

María Benítez, María L. Buil, Miguel A. Esteruelas, Ana M. López, Cristina Martín-Escura, and Enrique Oñate*

Departamento de Química Inorgánica, Instituto de Síntesis Química y Catálisis Homogénea (ISQCH), Centro de Innovación en Química Avanzada (ORFEO-CINQA), Universidad de Zaragoza – CSIC, 50009 Zaragoza, Spain

*Corresponding author's e-mail address: maester@unizar.es

CONTENTS

**Experimental Section: General Information**	S3
**Procedure for the α-Arylation of Amines**	S4
**NMR spectra**	S7
**Structural Analysis of Complexes 2d, 3, 5, 7, and 8**	S28
**Computational Details**	S30
**Energies of Optimized Structures of 2–7**	S31
**UV-Vis Spectra of Complexes 2–7 (Observed and Calculated)**	S34
**Analysis of Computed UV/Vis Data of Complexes 2–7**	S37
**Theoretical Analysis of Molecular Orbitals of Complexes 2–7**	S43
**Cyclic Voltammograms**	S55
**Normalized Excitation and Emission Spectra of Complexes 2–5 and 7**	S56
**Time-resolved Photoluminescence Decay of Complexes 2–5 and 7**	S62
**References**	S70

Experimental Section: General Information. All reactions were performed with rigorous exclusion of air at an argon/vacuum manifold using standard Schlenk-tube or glovebox techniques. Solvents were dried by the usual procedures and distilled under argon atmosphere or from an MBraun solvent purification apparatus. NMR spectra were recorded on a Bruker ARX 300 or Bruker Avance 300 MHz instruments. Chemical shifts (expressed in parts per million) are referenced to residual solvent peaks (^{1}H, $^{13}C\{^{1}H\}$) and external 85% H_3PO_4 ($^{31}P\{^{1}H\}$) or $CFCl_3$ (^{19}F). Coupling constants J and N ($N = J_{P\text{-}H} + J_{P'\text{-}H}$ for ^{1}H and $N = J_{P\text{-}C} + J_{P'\text{-}C}$ for $^{13}C\{^{1}H\}$) are given in Hertz. Elemental analyses were carried out in a Perkin-Elmer 2400-B Series II CHNS-Analyzer. High-resolution electrospray (HRMS) mass spectra were acquired using a MicroTOF-Q hybrid quadrupole time-of-flight spectrometer (Bruker Daltonics, Bremen, Germany). Attenuated total reflection infrared spectra (ATR-IR) of solid samples were run on a Perkin-Elmer Spectrum 100 FT-IR spectrometer. UV-visible spectra were registered on an Evolution 600 spectrophotomer. Steady-state photoluminescence spectra were recorded on a Jobin-Yvon Horiba Fluorolog FL-3-11 spectrofluorometer or with a PicoQuant FluoTime 300 spectrometer. Lifetimes were measured using an IBH 5000F coaxial nanosecond flash lamp or with a PicoQuant FluoTime 300 spectrometer. Data were fitted to either monoexponential or biexponential functions. Quantum yields were measured using the Hamamatsu Absolute PL Quantum Yield Measurement System C11347-11. Cyclic voltammetry measurements were performed using a Voltalab PST050 potentiostat with Pt wire as working electrode, Pt wire as counter electrode, and saturated calomel (SCE) as reference electrode. The experiments were carried out under argon in dichloromethane solutions (10^{-3} M), with Bu_4NPF_6 as supporting electrolyte (0.1 M). Scan rate was 100 $mV\cdot s^{-1}$. The potentials were referenced to the ferrocenium/ferrocene (Fc^{+}/Fc) couple.

Procedure for the α-Arylation of Amines: The reactions were carried out in schlenk tubes under argon atmosphere at room temperature. The schlenk tube equipped with a magnetic stir bar was charged with complex **3** (0.01 or 0.015 mmol, 2 or 3 mol%), the corresponding aromatic nitrile (0.5 mmol), sodium acetate (82 mg, 1.0 mmol; vacuum dried at 100 ºC for 12 h), 2.0 mL of dimethylacetamide (DMA), and the corresponding amine (1.5 mmol). The schlenk was placed inside a homemade photoreactor: blue irradiation was performed with Anmossi LED strip light (2.5 m of 24 V blue (465 nm) LED strip; 40 LEDs, max. output ca. 1.2 W) strapped around a 9 cm diameter polypropylene white canister. The reactor was installed on top of a stirring plate and the top of the canister was covered with aluminum foil. After 24 h, the reaction was diluted with ethyl acetate (20 mL) and added to a separatory funnel containing 25 mL of a saturated aqueous solution of Na_2CO_3. The layers were separated and the aqueous layer was extracted with EtOAc (3 x 10 mL). The combined organic extracts were washed with brine, dried ($MgSO_4$) and concentrated in vacuo. Purification of the crude product by flash chromatography on silica gel using the indicated solvent system afforded the desired α-arylated amine product.

Caution: NaCN formed as a byproduct is toxic and could lead to the release on HCN gas when treated with acid. Reactions should be conducted in a well-ventilated fume cupboard and aqueous cyanide-containing waste should be kept basic and disposed of in accord with institutional guidelines.

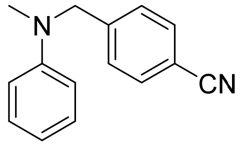

Preparation of 4-((Methyl(phenyl)amino)methyl)benzonitrile. Prepared according to the general procedure using 7.8 mg of **3** (0.01 mmol), 64 mg of 1,4-dicyanobenzene (0.5 mmol), 82 mg of sodium acetate (1.0 mmol), 190 µL of *N*,*N*-dimethylaniline (1.5 mmol) and 2.0 mL of DMA. After 24 h, the reaction mixture was subjected to the workup method defined in the general procedure and purified by column chromatography (silica gel: from 1% to 5% ethyl acetate

in hexanes) to afford a white syrup (89 mg, 0.40 mmol, 80%). ^{1}H-NMR (300 MHz, $CDCl_3$) δ 7.63 – 7.57 (m, 2H, H CNB), 7.37 – 7.30 (m, 2H, H CNB), 7.27 – 7.18 (m, 2H, H_m), 6.78 – 6.71 (m, 1H, H_p), 6.72 – 6.66 (m, 2H, H_o), 4.57 (s, 2H, NCH_2), 3.03 (s, 3H, NCH_3). ^{13}C-NMR (75 MHz, $CDCl_3$) δ 149.4 (C_q Ph), 145.1 (C_q CNB), 132.6 (CH CNB), 129.5 (CH_m), 127.5 (CH CNB), 119.0 (CN), 117.4 (CH_p), 112.6 (CH_o), 111.0 (C_q CNB), 56.8 (NCH_2), 39.0 (NCH_3). These NMR data agree with those previously reported for this compound.[1]

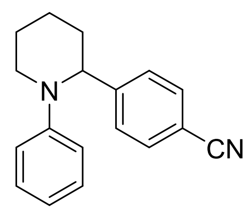

Preparation of 4-(1-Phenylpiperidin-2-yl)benzonitrile. Prepared according to the general procedure using 7.84 mg of iridium complex **3** (0.01 mmol, 0.02 equiv), 64 mg of 1,4-dicyanobenzene (0.5 mmol, 1.0 equiv), 80.6 mg of sodium acetate (1.0 mmol, 2.0 equiv), 242 µL of N-phenylpiperidine (1.5 mmol, 3.0 equiv) and 2.0 mL of DMA. After 24 h, the reaction mixture was subjected to the workup protocol outlined in the general procedure and purified by column chromatography (silica gel: from 2% to 5% ethyl acetate in hexanes) to afford a white syrup (107 mg, 0.41 mmol, 82%). ^{1}H-NMR (300 MHz, $CDCl_3$) δ 7.50 – 7.44 (m, 2H, H CNB), 7.39 – 7.34 (m, 2H, H CNB), 7.16 –7.08 (m, 2H, H_m), 6.89 – 6.83 (m, 2H, H_o), 6.83 – 6.77 (m, 1H, H_p), 4.39 (dd, J = 8.0, 4.0 Hz, 1H, NC*H*(Ph-4-CN)), 3.45 – 3.35 (m, 1H, CH_2N), 3.17 – 3.05 (m, 1H, CH_2N), 2.03 – 1.91 (m, 1H, $C\mathit{H}_2$CH(Ph-4-CN)), 1.86 – 1.65 (m, 4H, 2H $C\mathit{H}_2CH_2N$, 1H $C\mathit{H}_2CH_2CH_2N$ and 1H $C\mathit{H}_2$CH(Ph-4-CN)), 1.62 – 1.46 (m, 1H, $C\mathit{H}_2CH_2CH_2N$). ^{13}C-NMR (75 MHz, $CDCl_3$) δ 151.7 (C_q Ph), 150.1 (C_q CNB), 132.3 (CH CNB), 129.0 (CH_m), 128.1 (CH CNB), 121.1 (CH_p), 120.3 (CH_o), 119.1 (CN), 110.2 (C_q CNB), 61.9 (CHN), 52.7 (CH_2N), 34.4 ($\mathit{C}H_2$CH(Ph-4-CN)), 25.8 ($\mathit{C}H_2CH_2N$), 22.7 ($\mathit{C}H_2CH_2CH_2N$). These NMR data agree with those previously reported for this compound.[1]

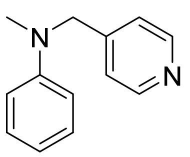

Preparation of 4-((Methyl(phenyl)amino)methyl)pyridine. Prepared according to the general procedure using 7.8 mg of **3** (0.01 mmol), 52.1 mg of 4-cyanopyridine (0.5 mmol), 82 mg of sodium acetate (1.0 mmol), 190 µL of *N*,*N*-dimethylaniline (1.5 mmol) and 2.0 mL of DMA. After 24 h, the reaction mixture was subjected to the workup method defined in the general procedure and purified by column chromatography (silica gel: from 40% to 70% ethyl acetate in hexanes) to afford a white syrup (77 mg, 0.39 mmol, 78%). ^{1}H-NMR (300 MHz, $CDCl_3$) δ 8.55 – 8.48 (m, 2H, py), 7.26 – 7.18 (m, 2H, H_m), 7.17 – 7.12 (m, 2H, py), 6.77 – 6.70 (m, 1H, H_p), 6.71 – 6.65 (m, 2H, H_o), 4.50 (s, 2H, NCH_2), 3.04 (s, 3H, NCH_3). ^{13}C-NMR (75 MHz, $CDCl_3$) δ 150.1 (CH py), 149.3 (C_q Ph), 148.6 (C_q py), 129.4 (CH_m), 121.9 (CH py), 117.3 (CH_p), 112.5 (CH_o), 56.1 (NCH_2), 39.0 (NCH_3).[2]

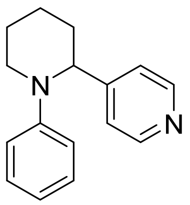

Preparation of 4-(1-Phenylpiperidin-2-yl)pyridine. Prepared according to the general procedure using 7.84 mg of iridium complex **3** (0.015 mmol), 52.1 mg of 4-cyanopyridine (0.5 mmol), 80.6 mg of sodium acetate (1.0 mmol), 242 µL of N-phenylpiperidine (1.5 mmol) and 2.0 mL of DMA. After 24 h, the reaction mixture was subjected to the workup protocol outlined in the general procedure and purified by column chromatography (silica gel: from 10% to 50% ethyl acetate in hexanes) to afford a white syrup (67 mg, 0.28 mmol, 56%). HRMS (electrospray, *m/z*): Calcd for $C_{16}H_{18}N_2$ $[M+H]^+$: 239.1543; found: 239.1531. ^{1}H-NMR (300 MHz, $CDCl_3$) δ 8.47 – 8.40 (m, 2H, H py), 7.22 – 7.17 (m, 2H, H py), 7.17 – 7.10 (m, 2H, H_m), 6.91 – 6.84 (m, 2H, H_o), 6.84 – 6.77 (m, 1H, H_p), 4.46 (dd, *J* = 7.2, 4.3 Hz, 1H, NC*H*(Ph-4-CN)), 3.37 (m, 1H, CH_2N), 3.21 (m, 1H, CH_2N), 2.05–1.93 (m, 1H, C*H*2CH(Ph-4-CN)), 1.93–1.83 (m, 1H, C*H*2CH(Ph-4-CN)), 1.80–1.72 (m, 2H, C*H*2CH2N), 1.71–1.62 (m, 1H, C*H*2CH2CH2N), 1.62–1.51 (m, 1H, C*H*2CH2CH2N). ^{13}C-NMR (75 MHz, $CDCl_3$) δ 153.2 (C_q py), 151.6 (C_q Ph), 150.0 (CH py), 129.1 (CH_m), 122.7 (CH py), 120.6 (CH_p), 119.3

(CH_o), 60.7 (CHN), 51.3 (CH_2N), 33.4 (*C*H_2CH(Ph-4-CN)), 25.7 (*C*H_2CH_2N), 22.3 (*C*$H_2CH_2CH_2N$).

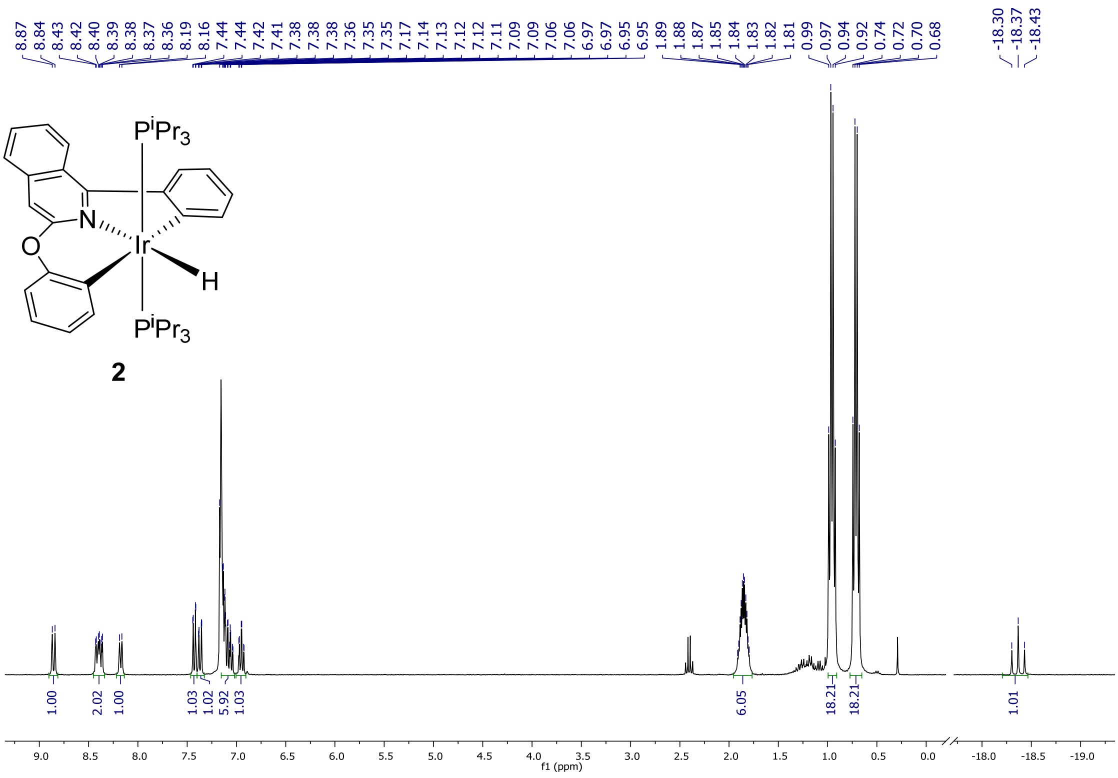

Figure S1. ^{1}H-NMR (300 MHz, C_6D_6, 298 K) of complex **2**.

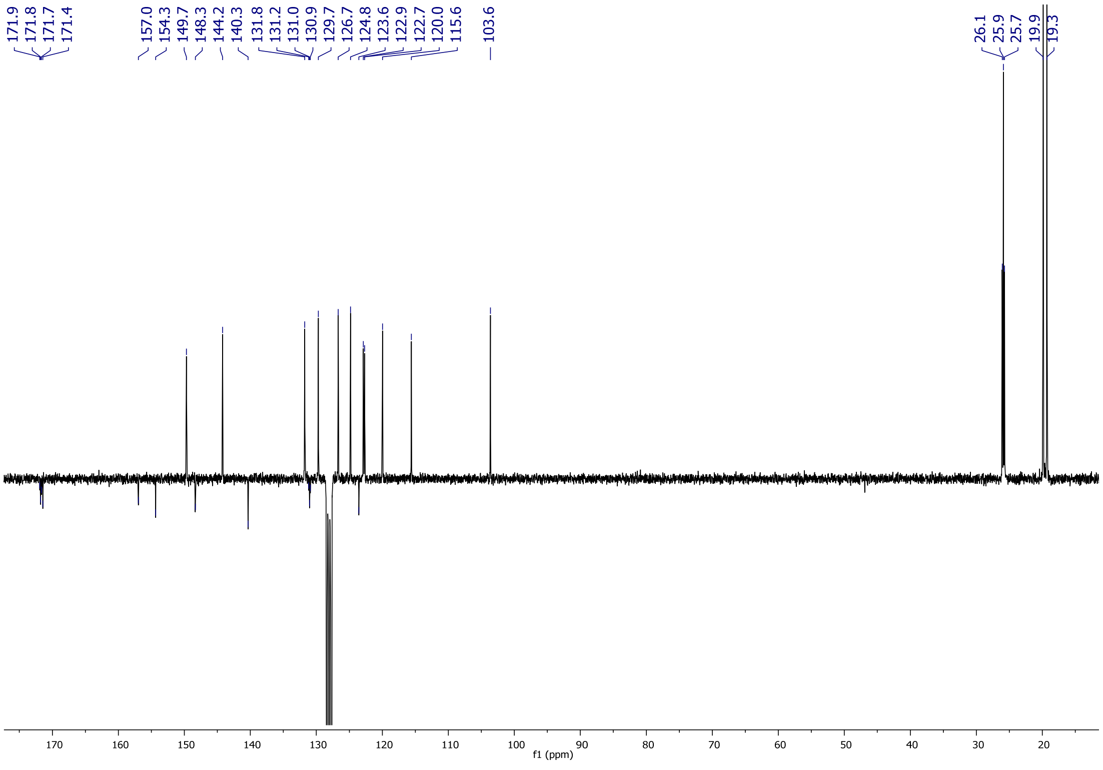

Figure S2. $^{13}C\{^1H\}$-APT NMR (75.5 MHz, C_6D_6, 298 K) of complex **2**.

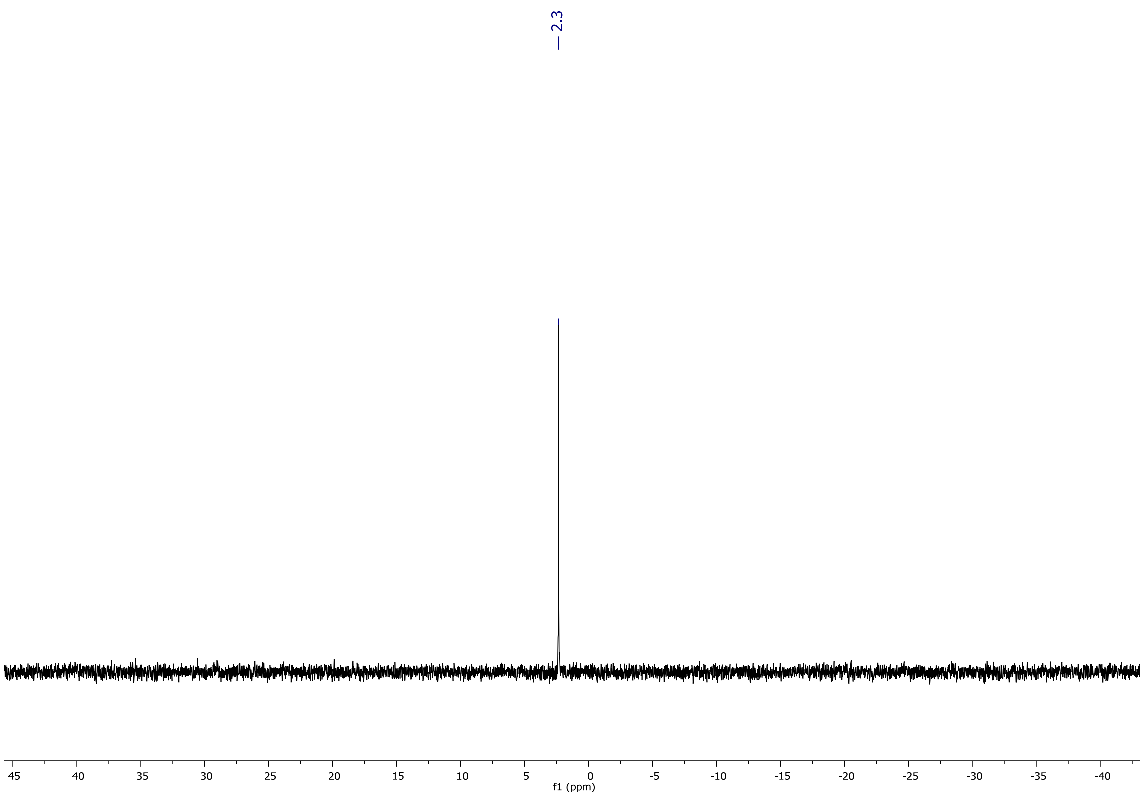

Figure S3. $^{31}P\{^{1}H\}$-NMR (121.5, MHz, C_6D_6, 298 K) of complex **2**.

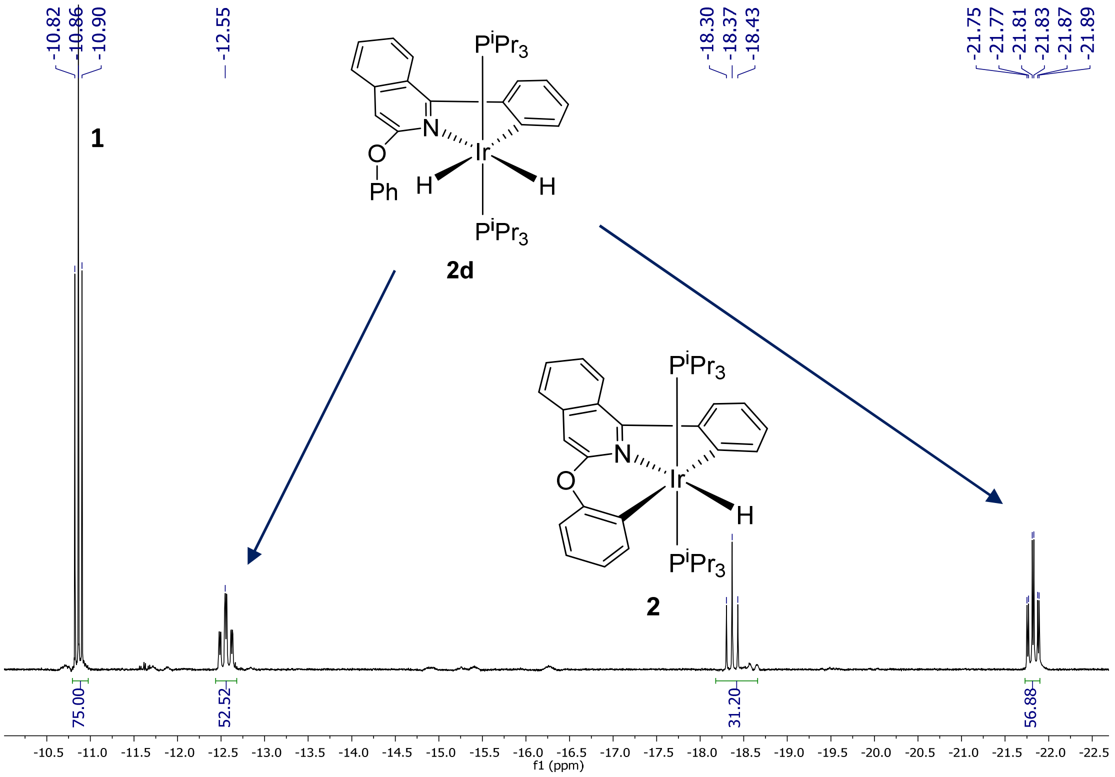

Figure S4. Hydride region of the ^{1}H NMR spectrum (300 MHz, C_6D_6, 298 K) of the reaction's crude between complex **1** and 3-phenoxy-1-phenylisoquinoline, after 24 h in toluene under reflux.

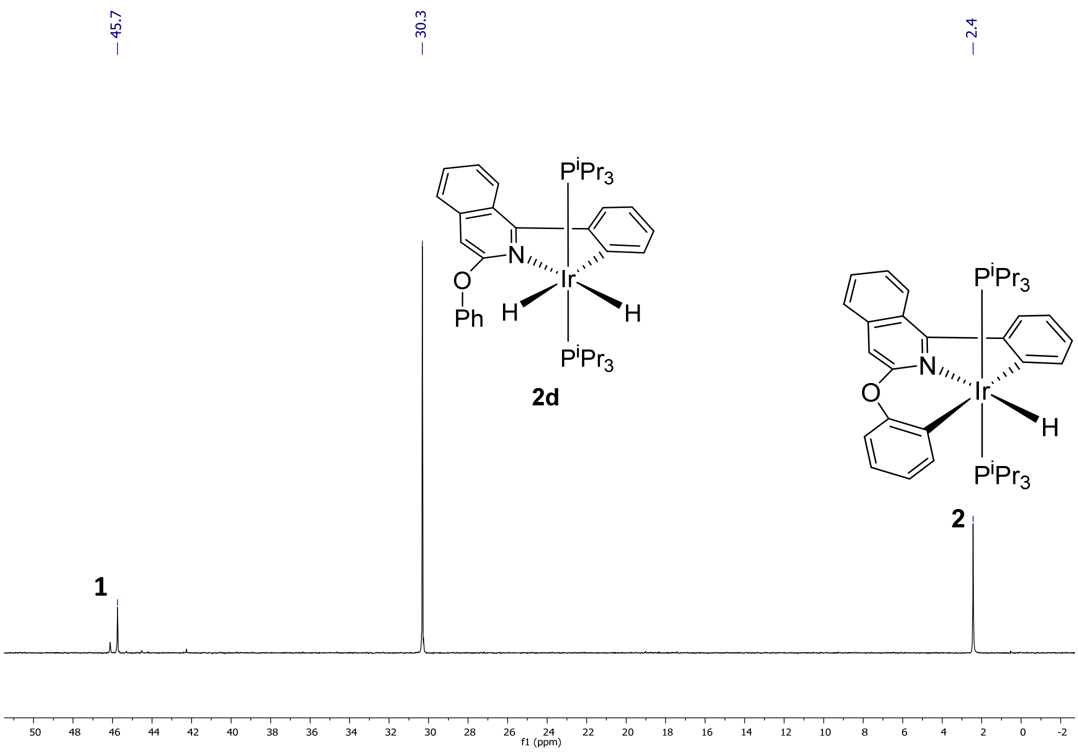

Figure S5. $^{31}P\{^{1}H\}$-NMR spectrum (121.49 MHz, C_6D_6, 298 K) of the reaction's crude between complex **1** and 3-phenoxy-1-phenylisoquinoline, after 24 h in toluene under reflux.

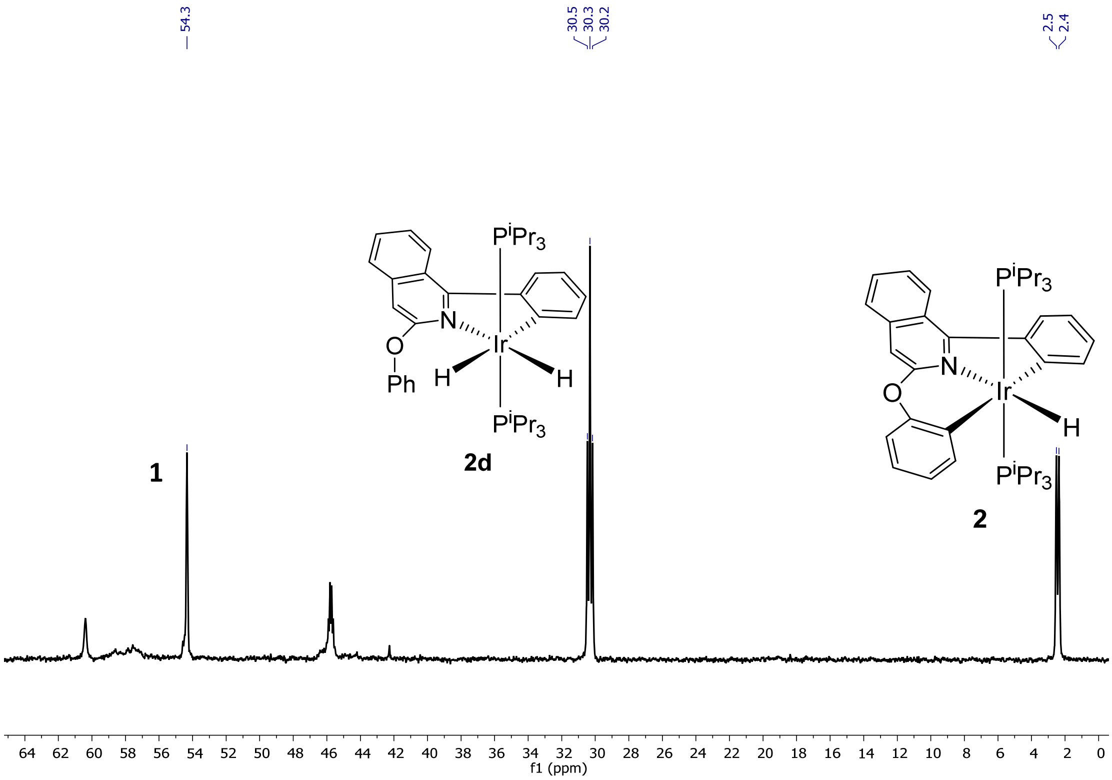

Figure S6. Off-resonance ^{31}P-NMR spectrum (121.49 MHz, C_6D_6, 298 K) of the reaction's crude between complex **1** and 3-phenoxy-1-phenylisoquinoline, after 24 h in toluene under reflux.

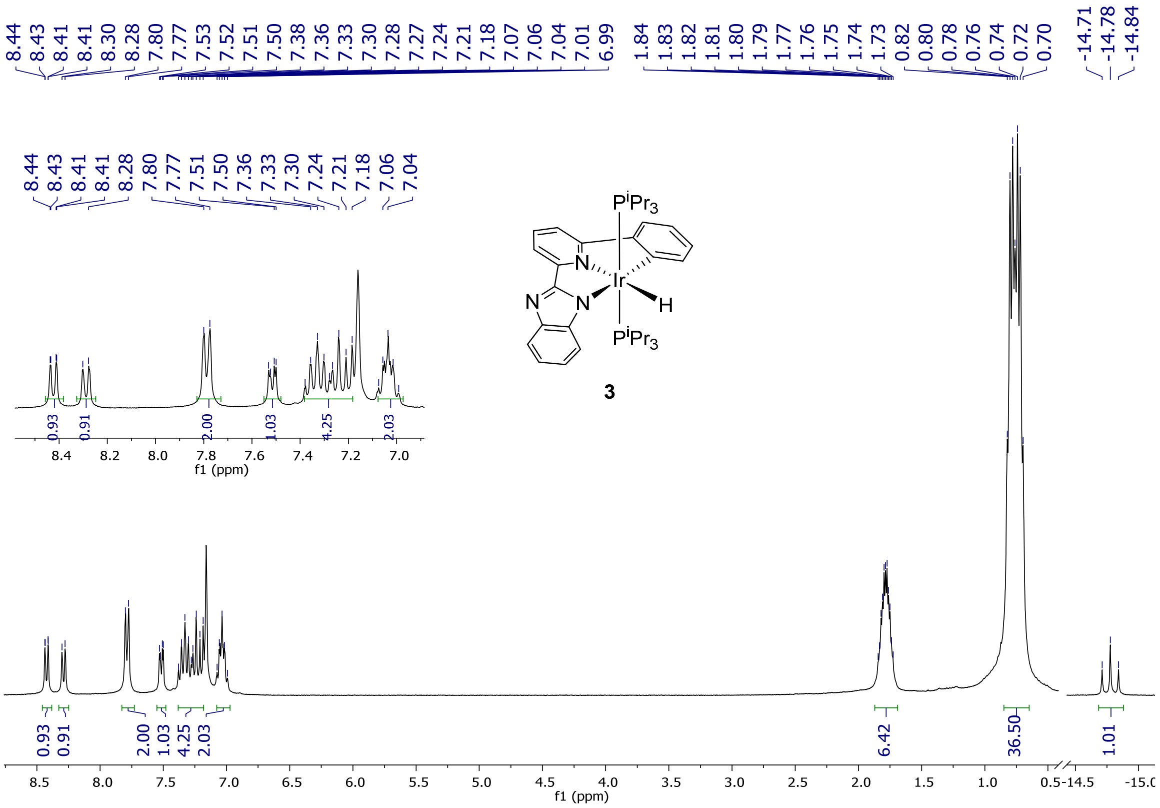

Figure S7. ^1H NMR (300 MHz, C_6D_6, 298 K) of complex **3**.

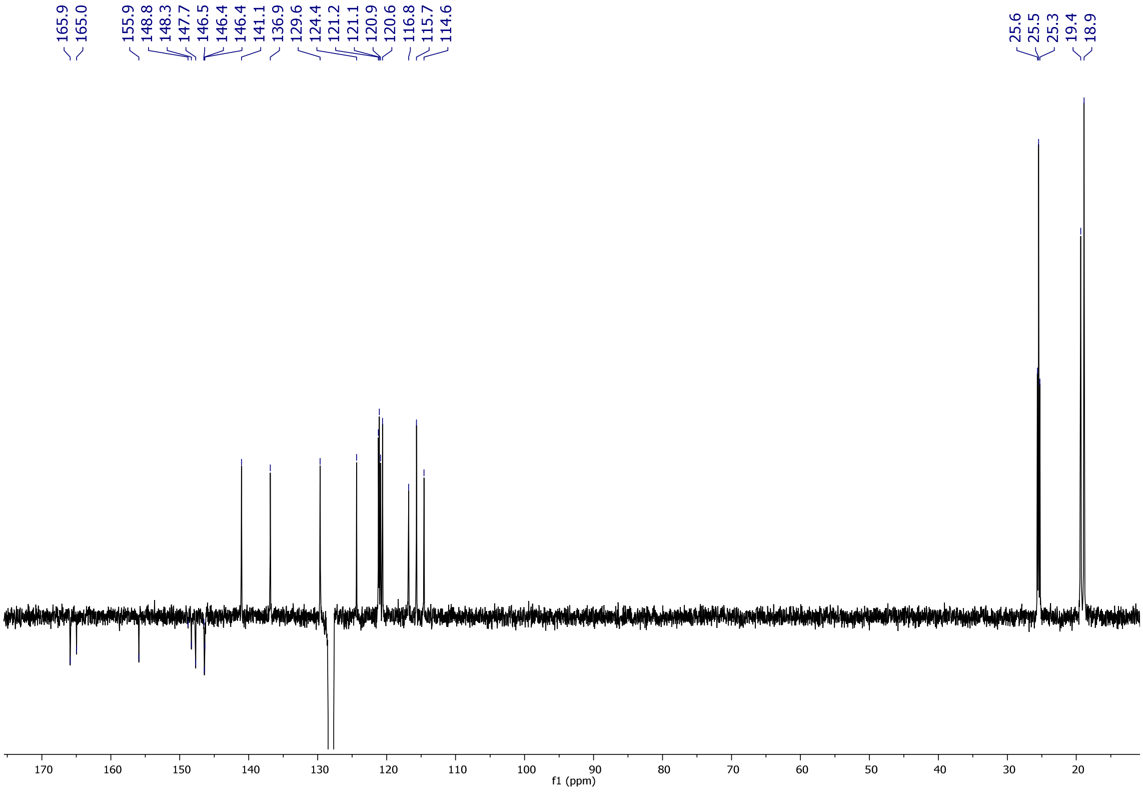

Figure S8. $^{13}C\{^1H\}$-APT NMR (75.5 MHz, C_6D_6, 298 K) of complex **3**.

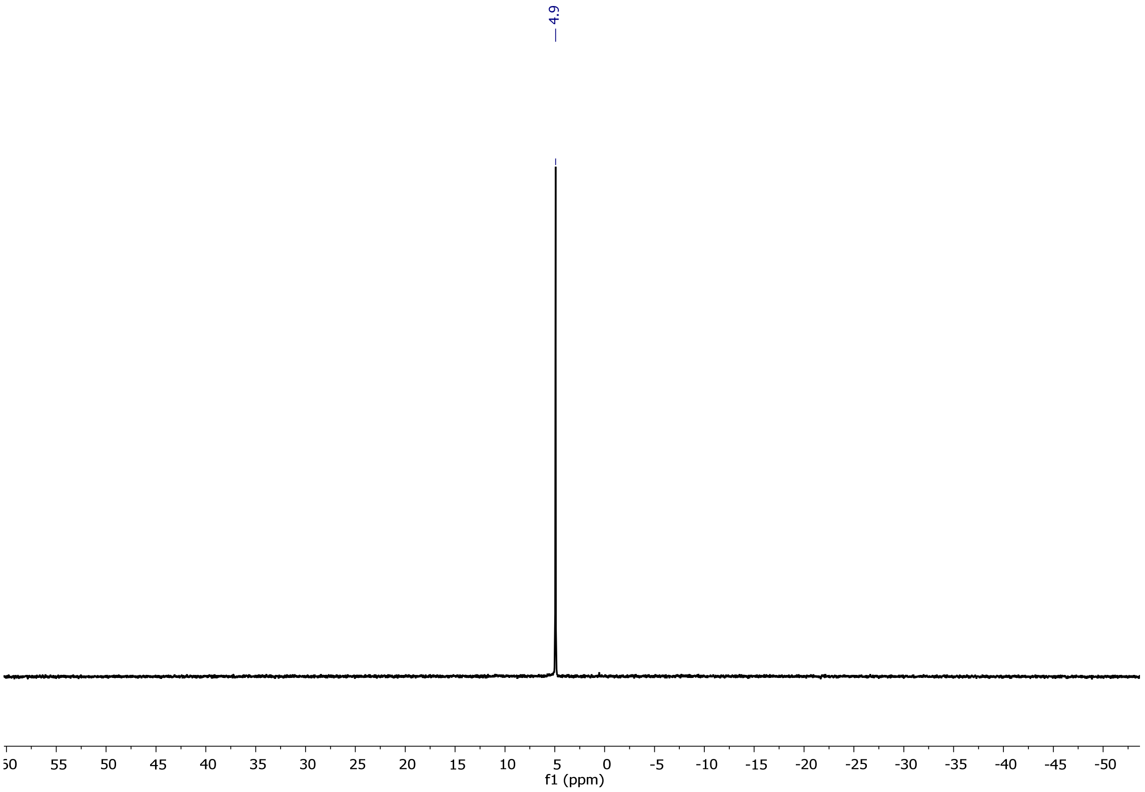

Figure S9. $^{31}P\{^{1}H\}$-NMR (121.5 MHz, C_6D_6, 298 K) of complex **3**.

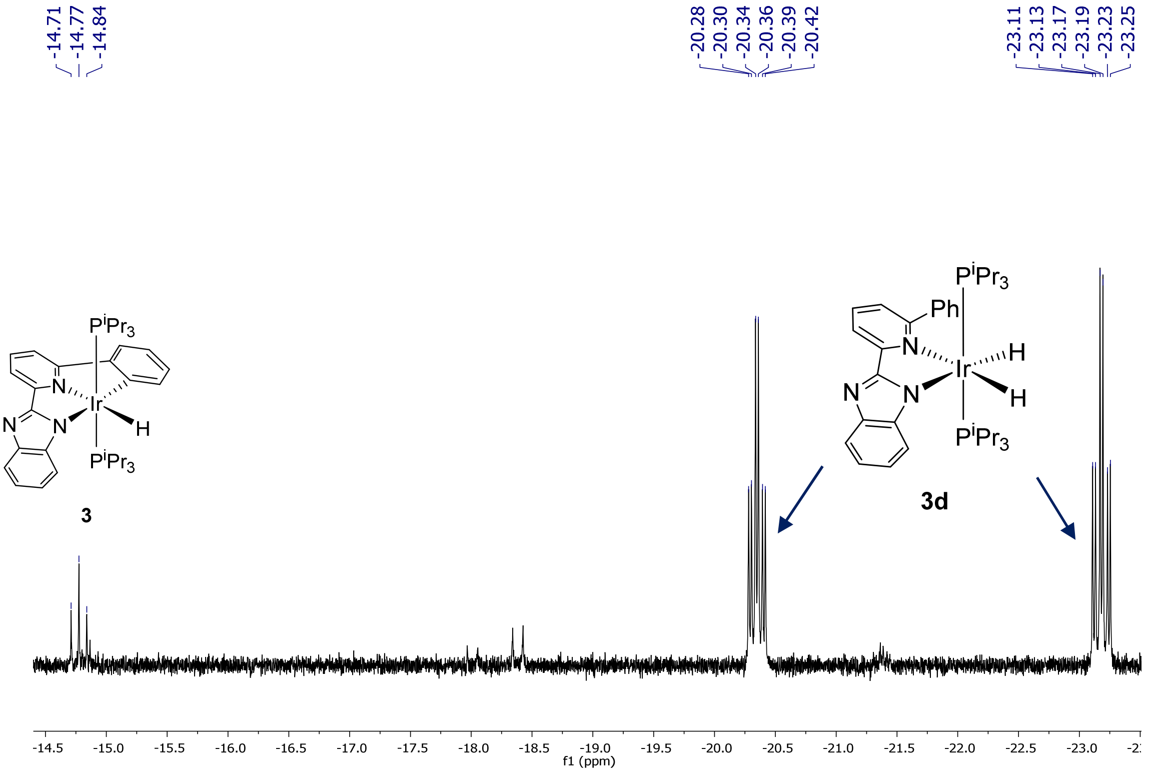

Figure S10. Hydride region of the ^{1}H NMR spectrum (300 MHz, C_6D_6, 298 K) of the reaction's crude between complex **1** and 2-(1*H*-benzimidazol-2-yl)-6-phenylpyridine, after 21 h in toluene under reflux.

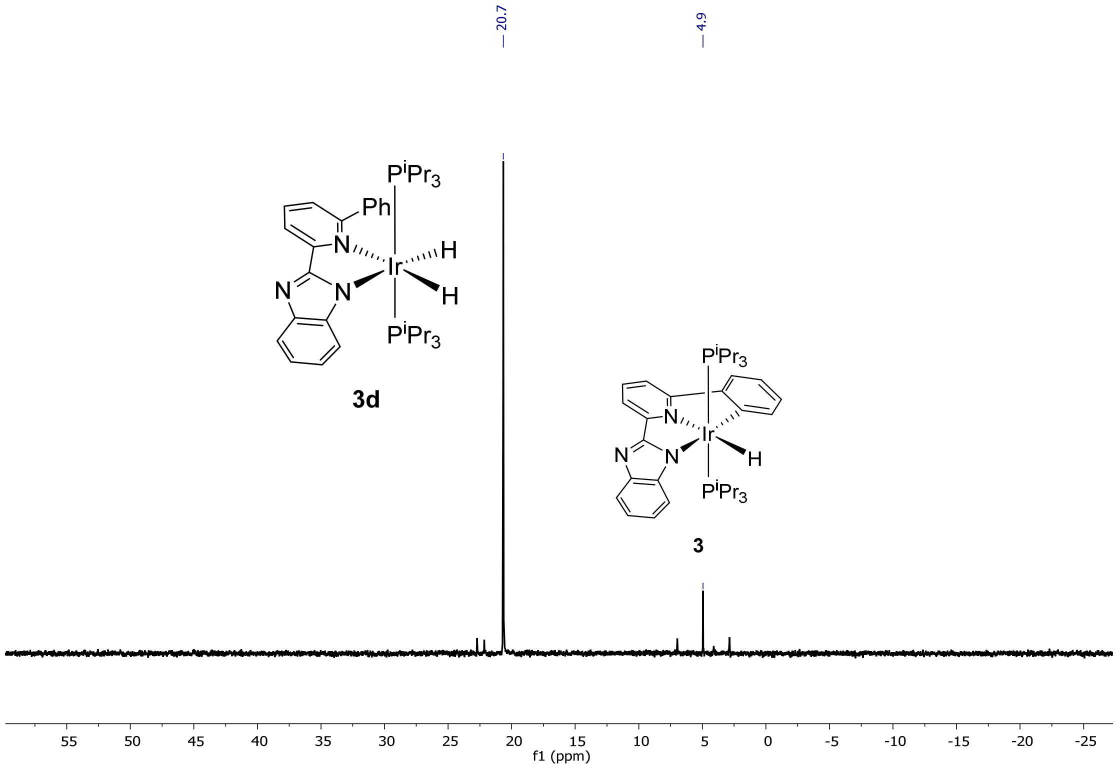

Figure S11. $^{31}P\{^1H\}$-NMR spectrum (121.49 MHz, C_6D_6, 298 K) of the reaction's crude between complex **1** and 2-(1*H*-benzimidazol-2-yl)-6-phenylpyridine, after 21 h in toluene under reflux.

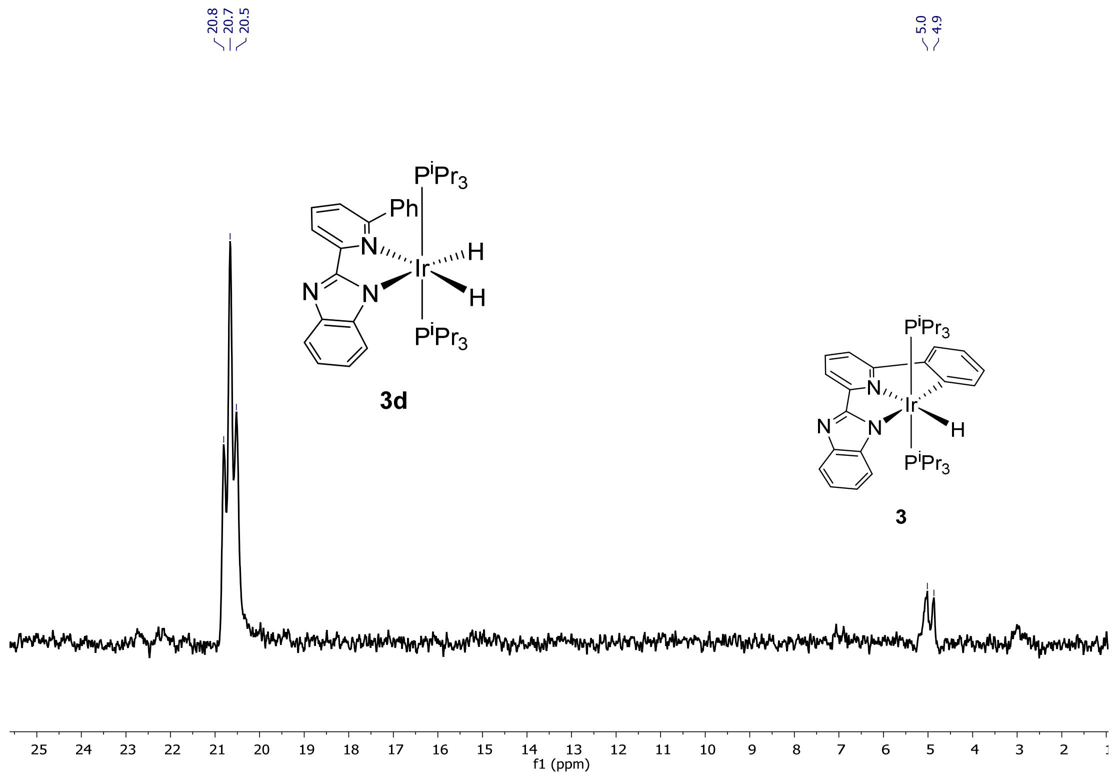

Figure S12. Off-resonance ^{31}P-NMR spectrum (121.49 MHz, C_6D_6, 298 K) of the reaction's crude between complex **1** and 2-(1*H*-benzimidazol-2-yl)-6-phenylpyridine, after 21 h in toluene under reflux.

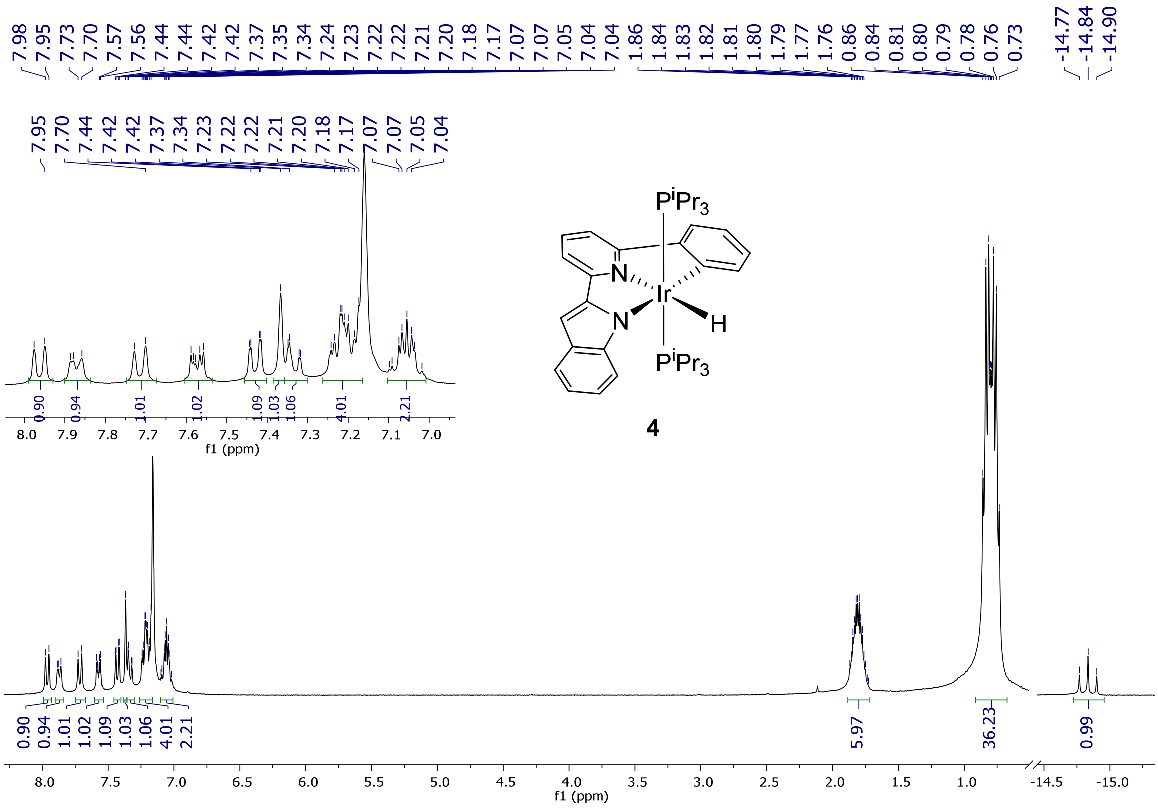

Figure S13. ^{1}H NMR (300 MHz, C_6D_6, 298 K) of complex **4**.

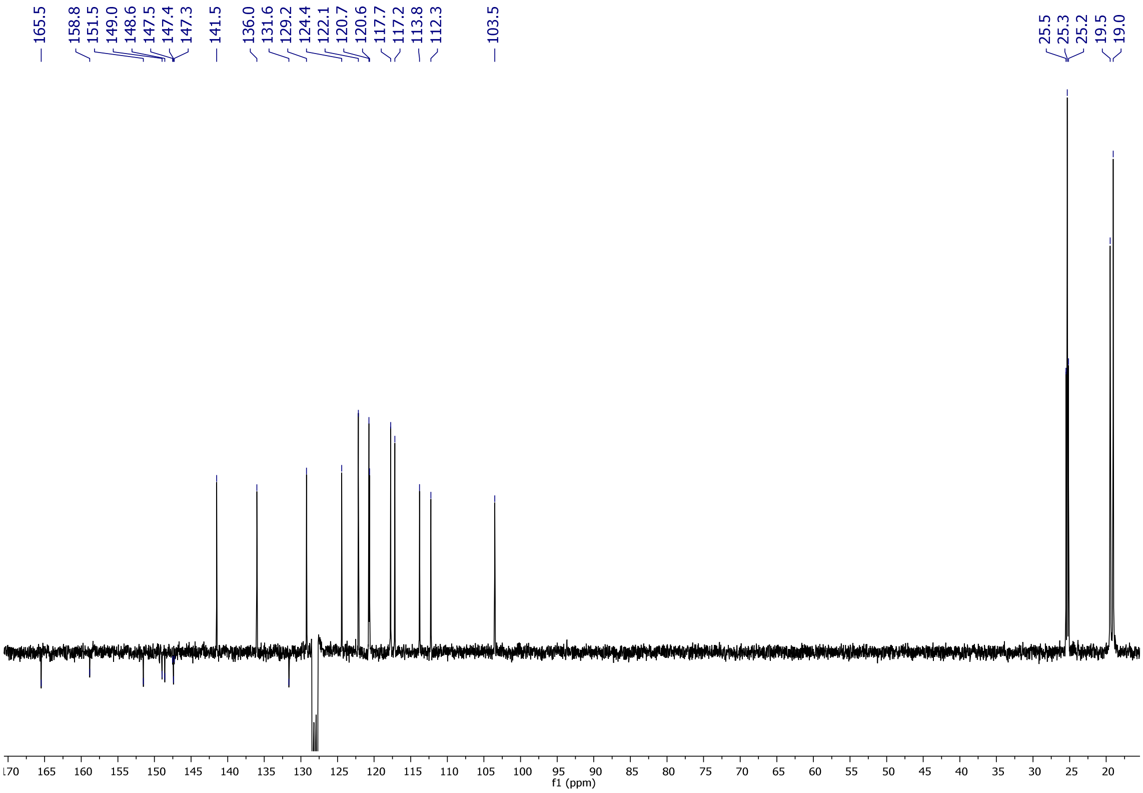

Figure S14. $^{13}C\{^{1}H\}$-APT NMR(75.5 MHz, C_6D_6, 298 K) of complex **4**.

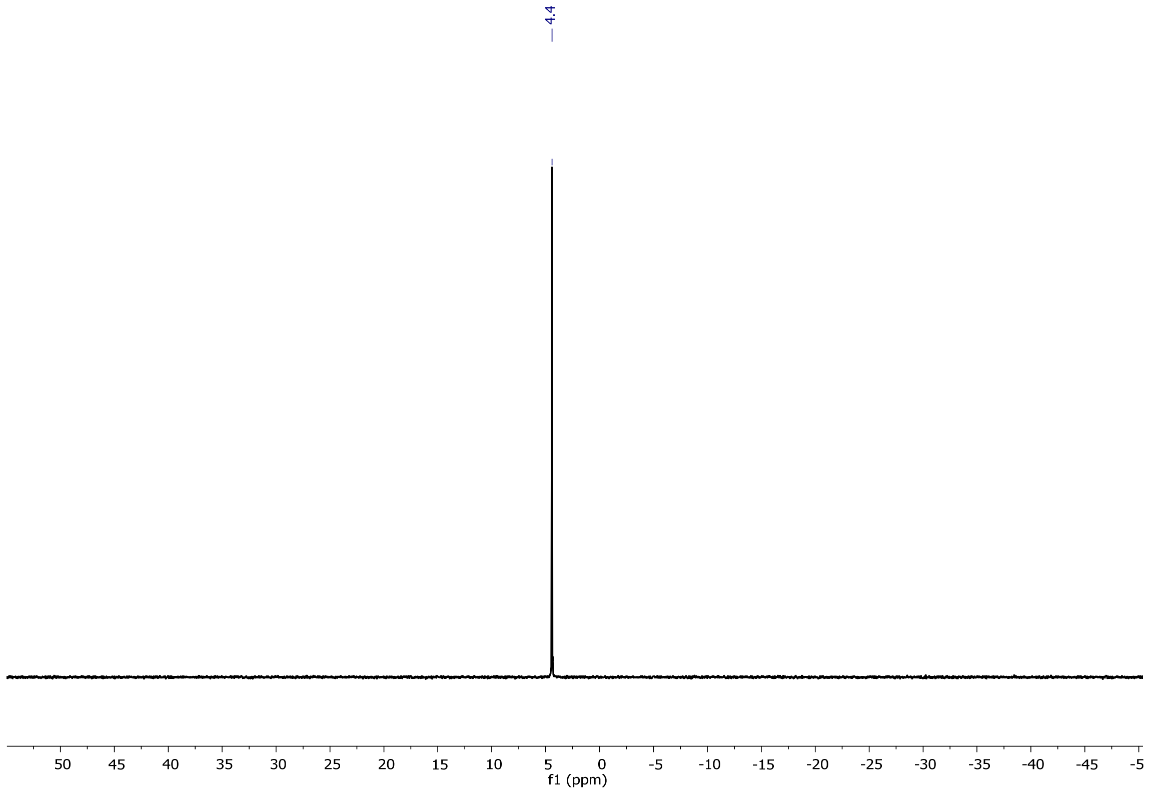

Figure S15. $^{31}P\{^{1}H\}$-NMR (121.5 MHz, C_6D_6, 298 K) of complex **4**.

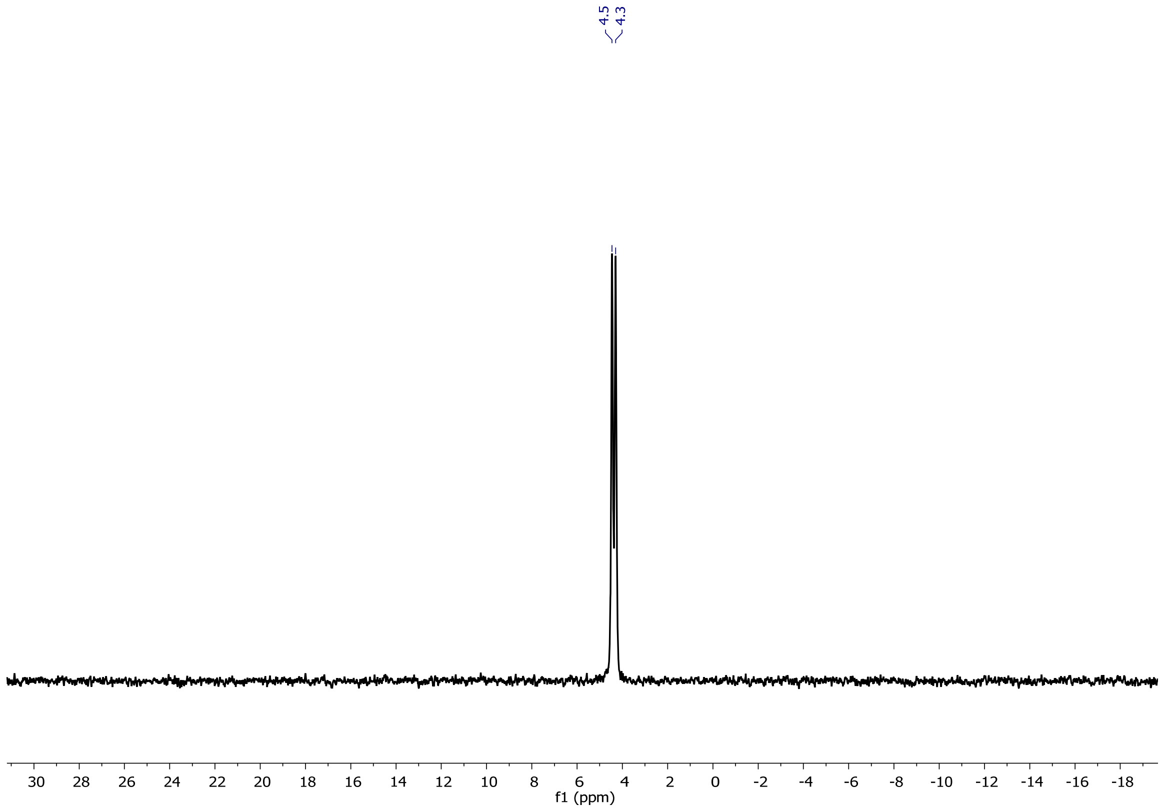

Figure S16. Off-resonance $^{31}P\{^{1}H\}$-NMR (121.5 MHz, C_6D_6, 298 K) of complex **4**.

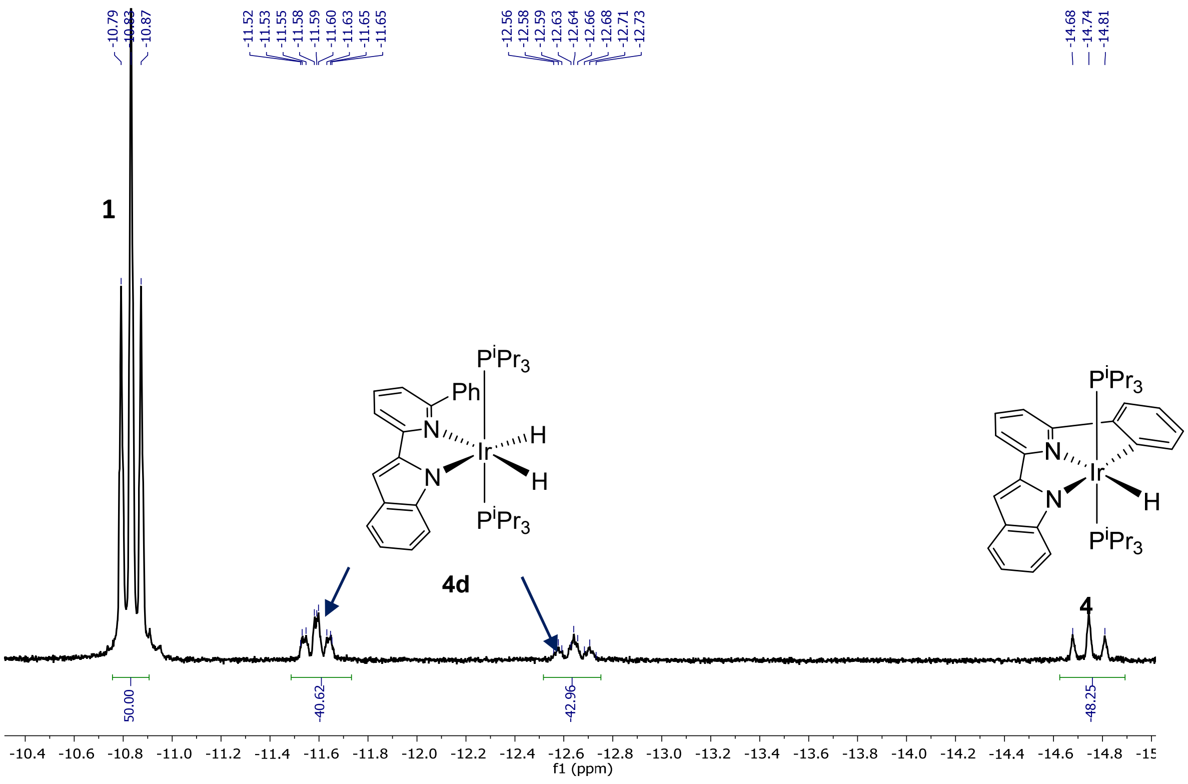

Figure S17. Hydride region of the ^{1}H NMR spectrum (300 MHz, toluene, 298 K) of the reaction's crude between complex **1** and 2-(1*H*-indol-2-yl)-6-phenyl-pyridine, after 3 h in toluene under reflux.

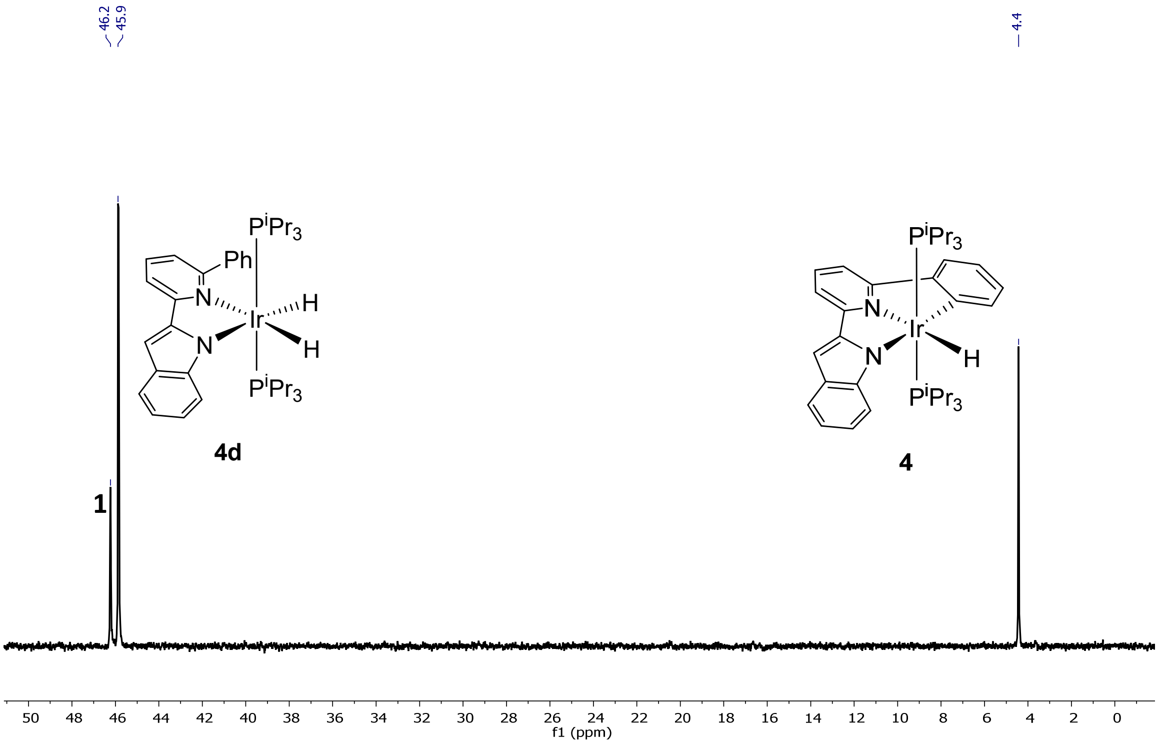

Figure S18. $^{31}P\{^{1}H\}$-NMR spectrum (121.49 MHz, toluene, 298 K) of the reaction's crude between complex **1** and 2-(1*H*-indol-2-yl)-6-phenyl-pyridine, after 3 h in toluene under reflux.

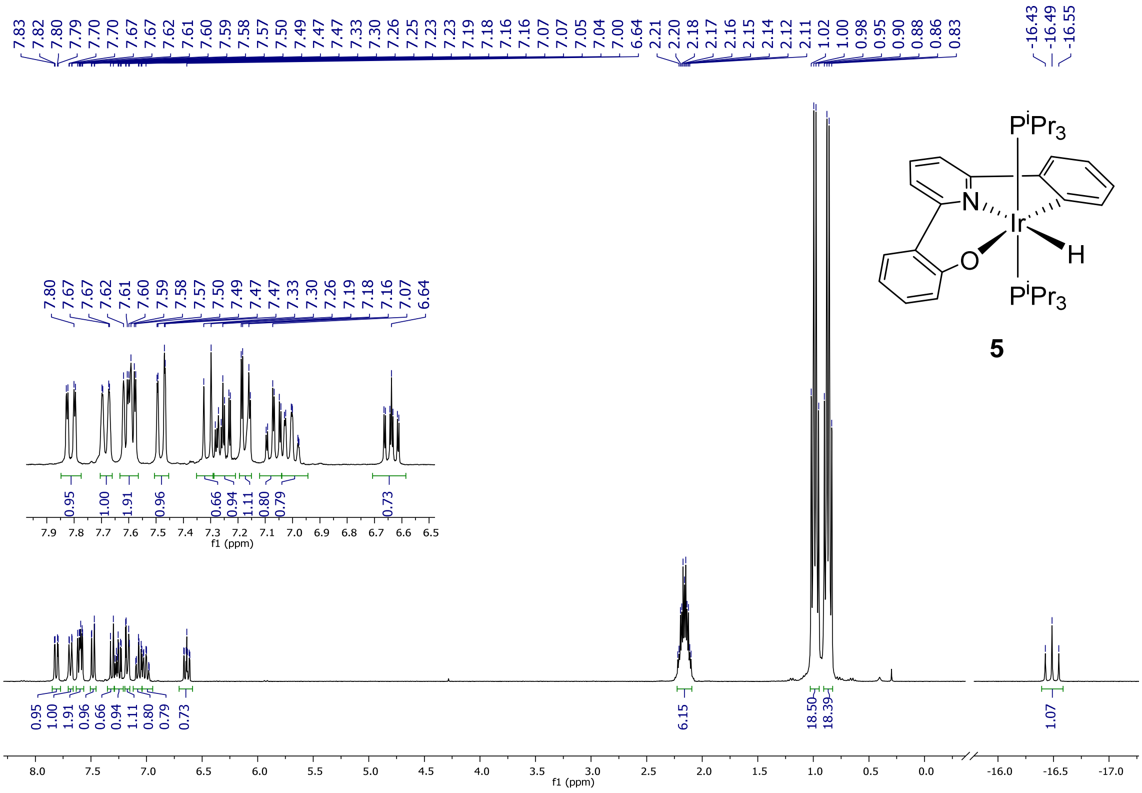

Figure S19. ^{1}H-NMR (300 MHz, C_6D_6, 298 K) of complex **5**.

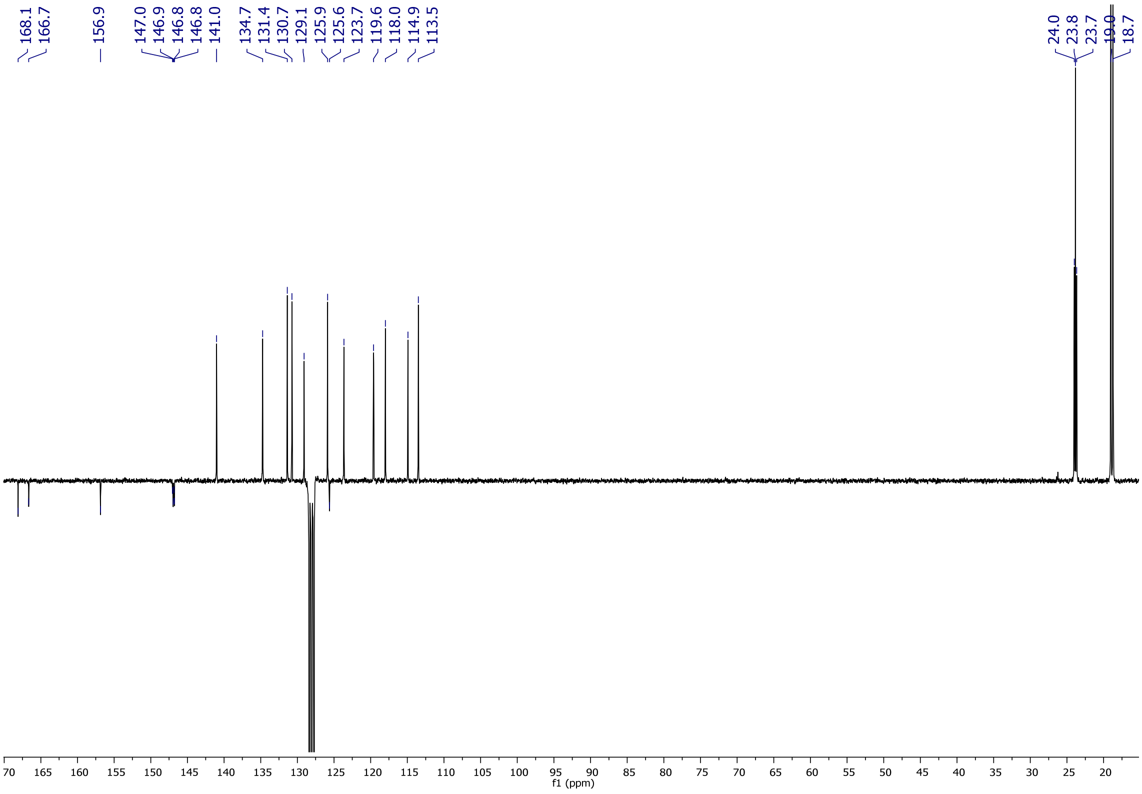

Figure S20. $^{13}C\{^{1}H\}$-APT NMR (75.5 MHz, C_6D_6, 298 K) of complex **5**.

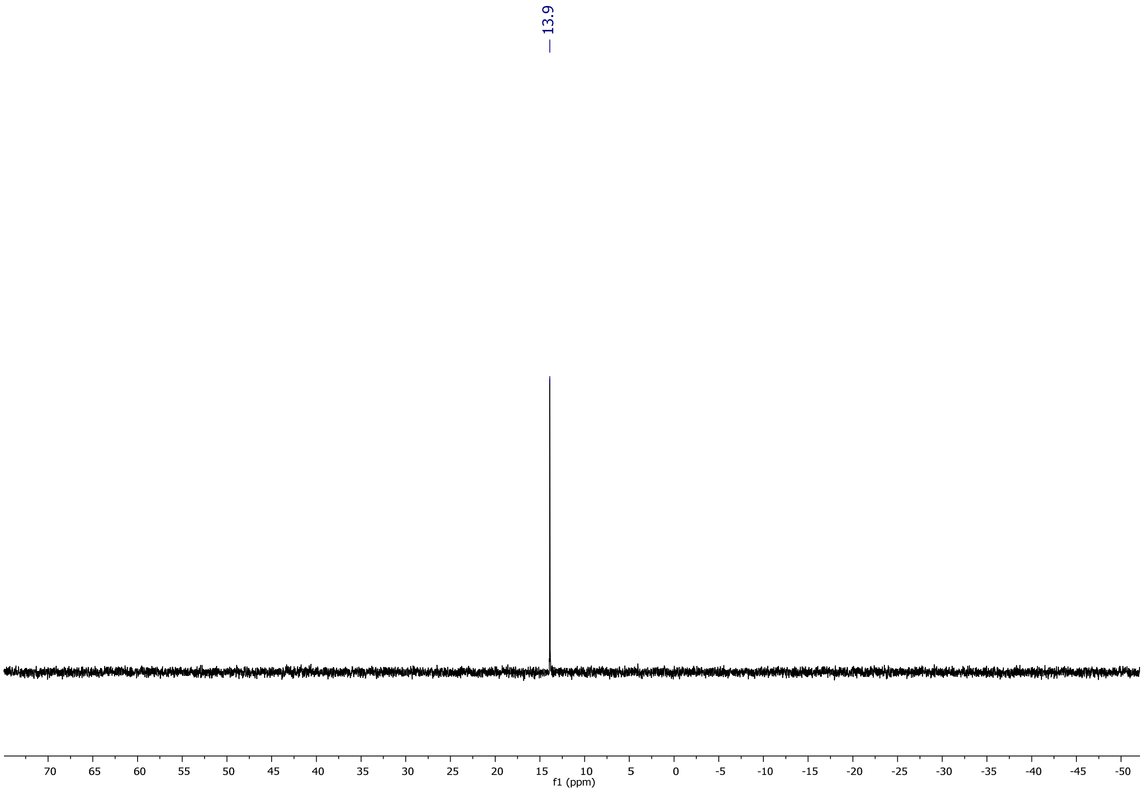

Figure S21. $^{31}P\{^{1}H\}$-NMR (121.5, MHz, C_6D_6, 298 K) of complex **5**.

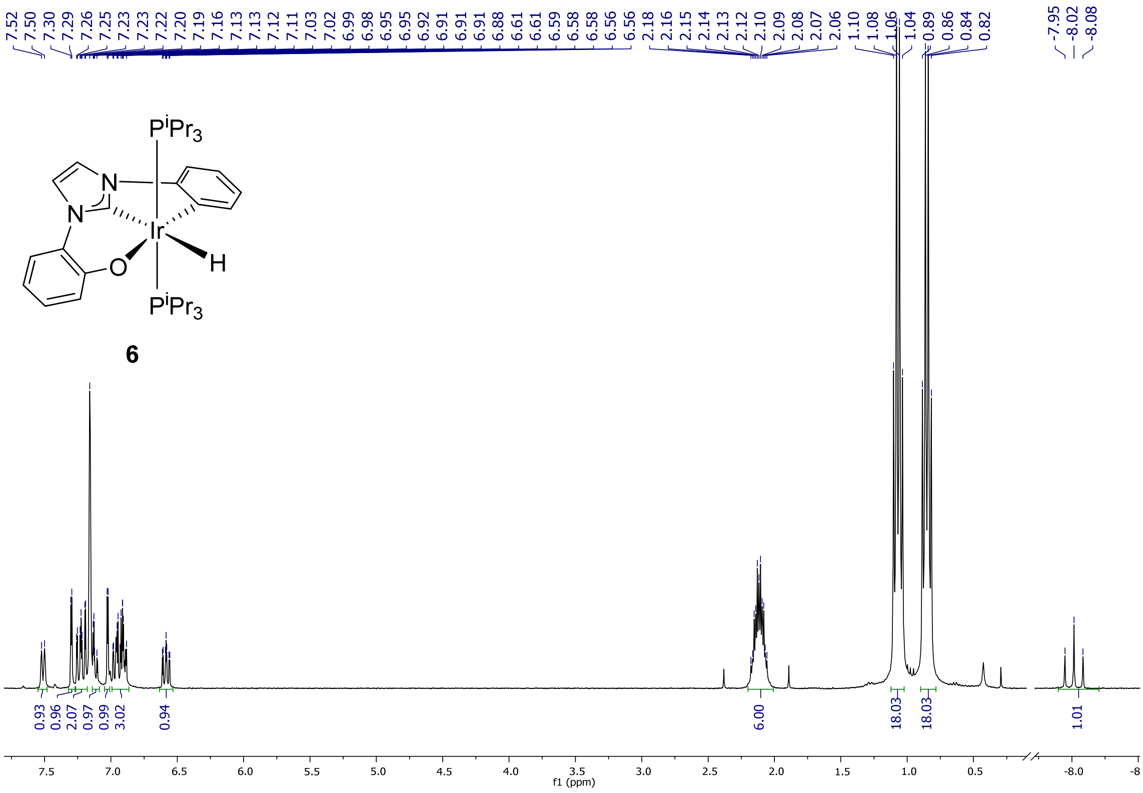

Figure S22. ^{1}H-NMR (300 MHz, C_6D_6, 298 K) of complex **6**.

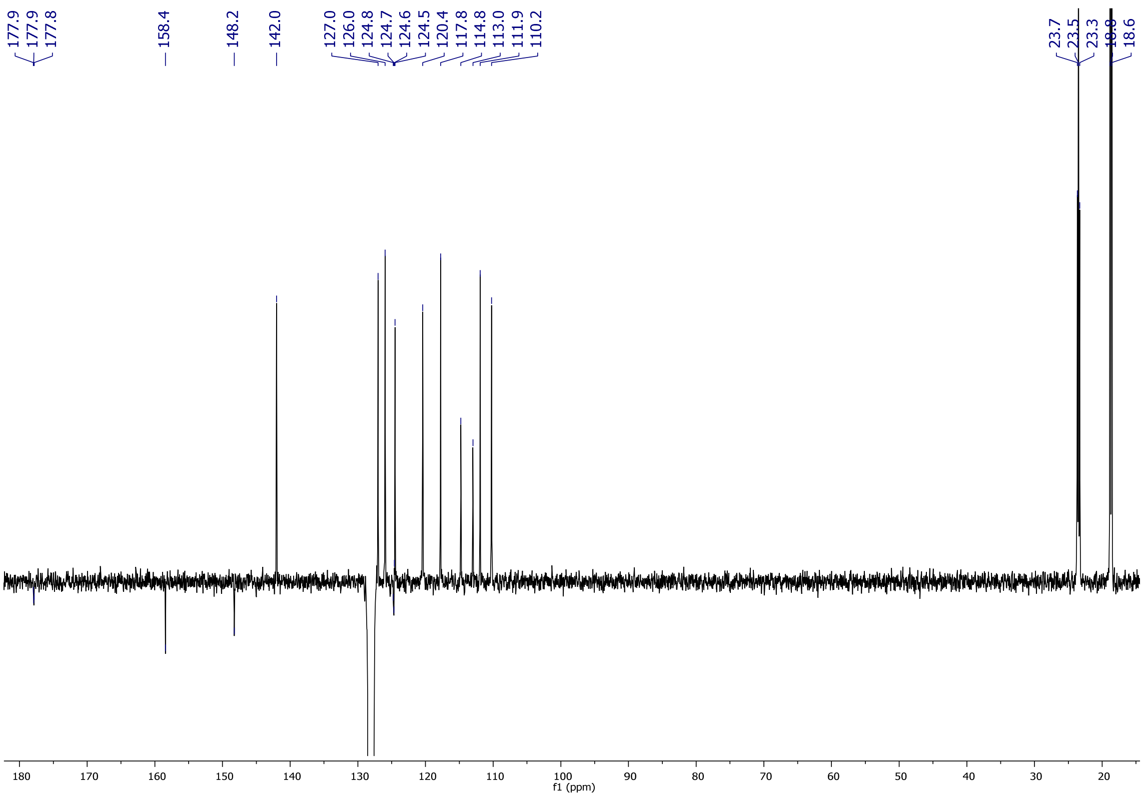

Figure S23. $^{13}C\{^{1}H\}$-APT NMR (75.5 MHz, C_6D_6, 298 K) of complex **6**.

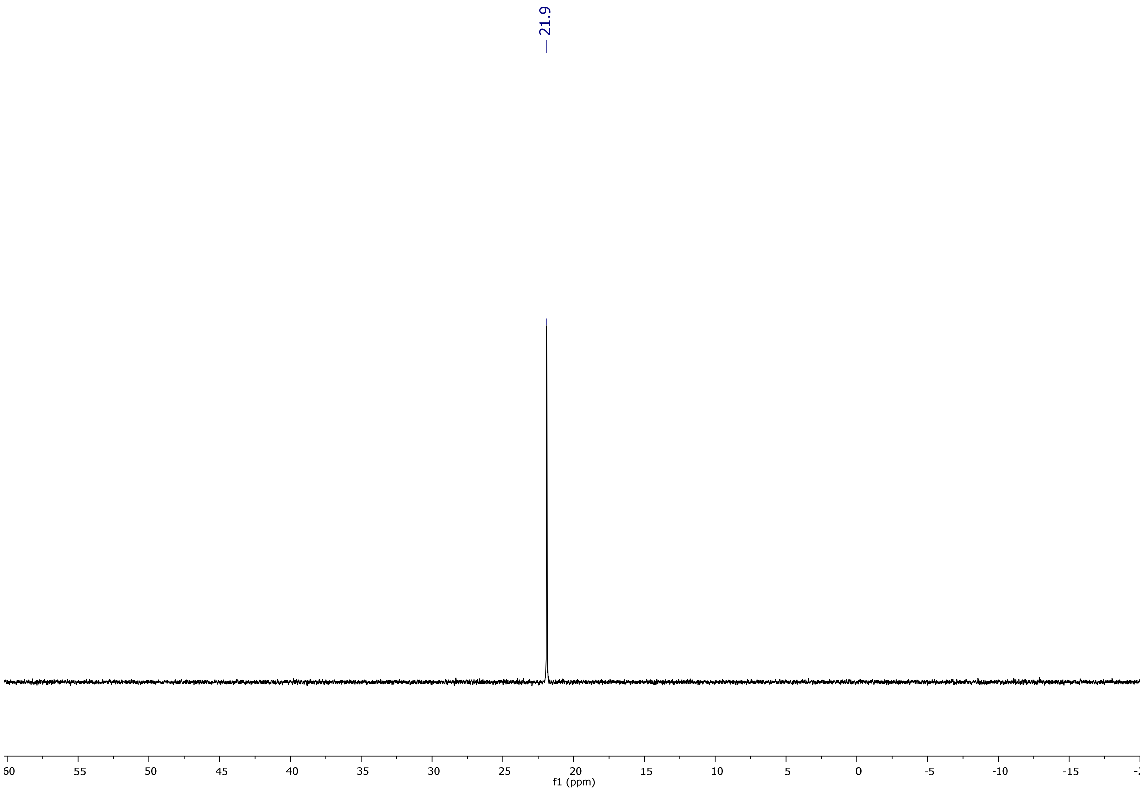

Figure S24. $^{31}P\{^{1}H\}$-NMR (121.5, MHz, C_6D_6, 298 K) of complex **6**.

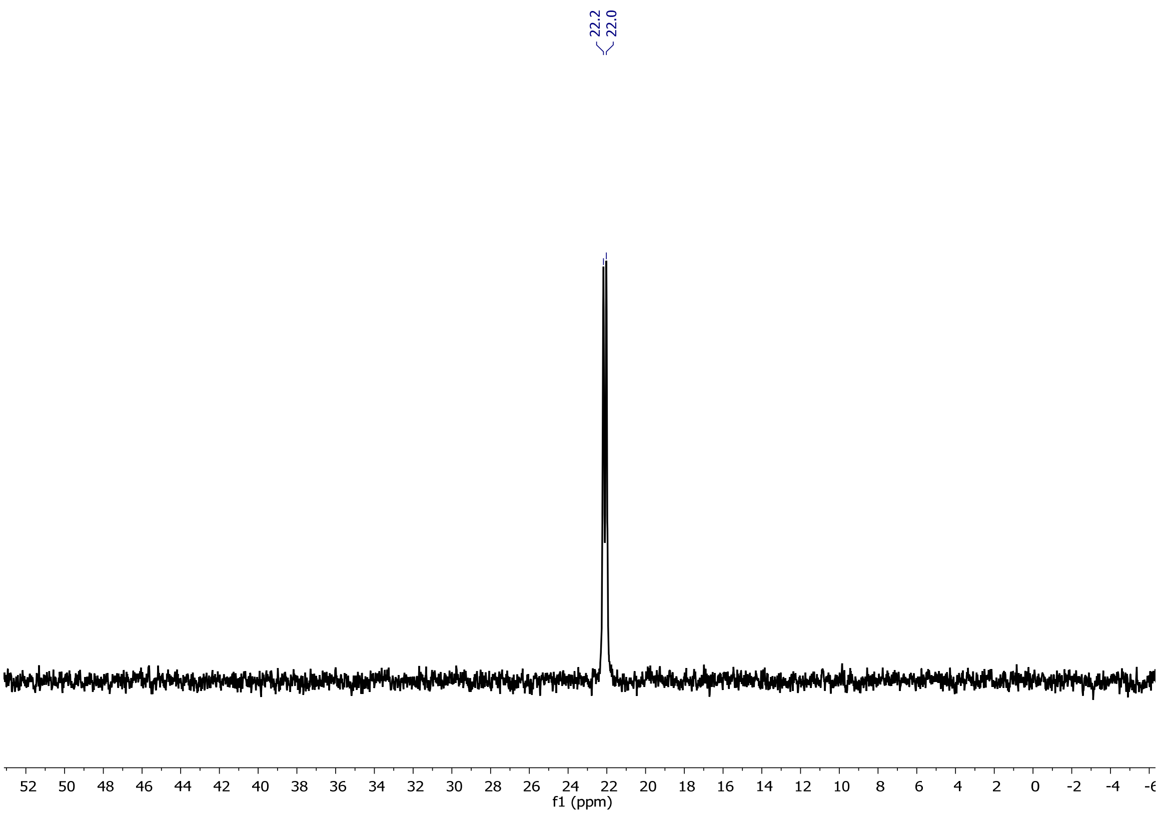

Figure S25. Off-resonance $^{31}P\{^1H\}$-NMR (121.5, MHz, C_6D_6, 298 K) of complex **6**.

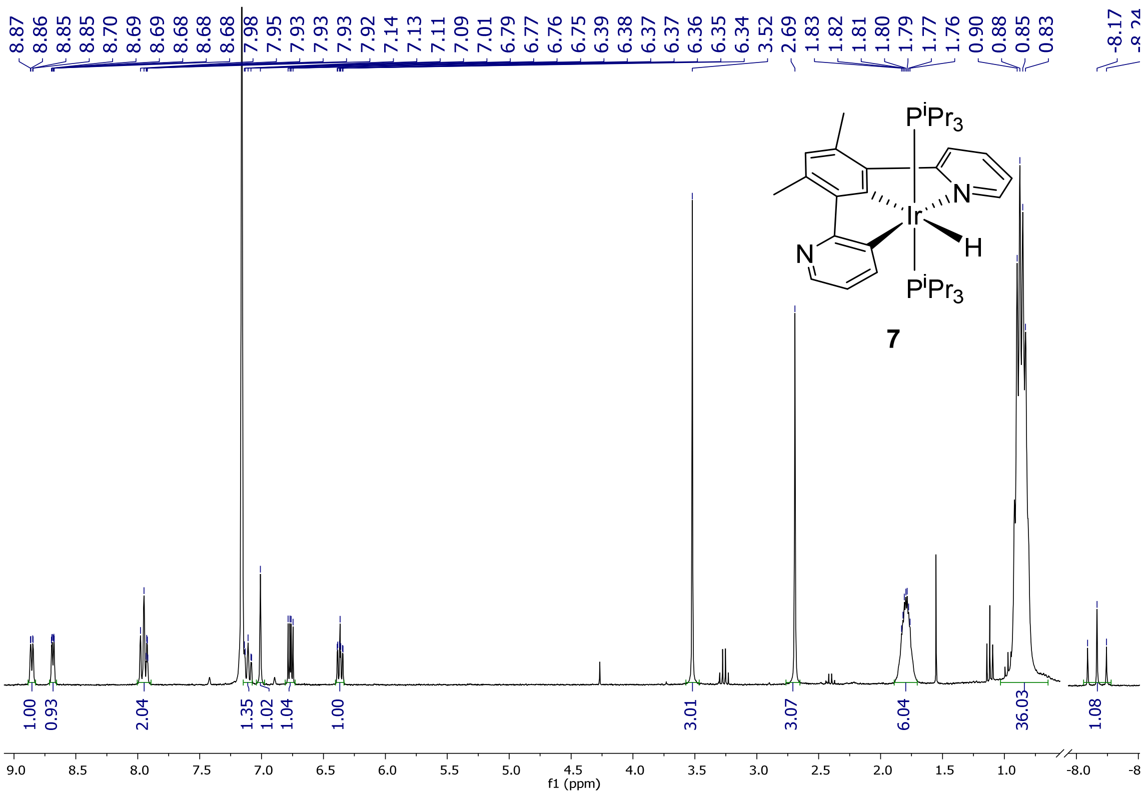

Figure S26. ^1H-NMR (300 MHz, C_6D_6, 298 K) of complex **7**.

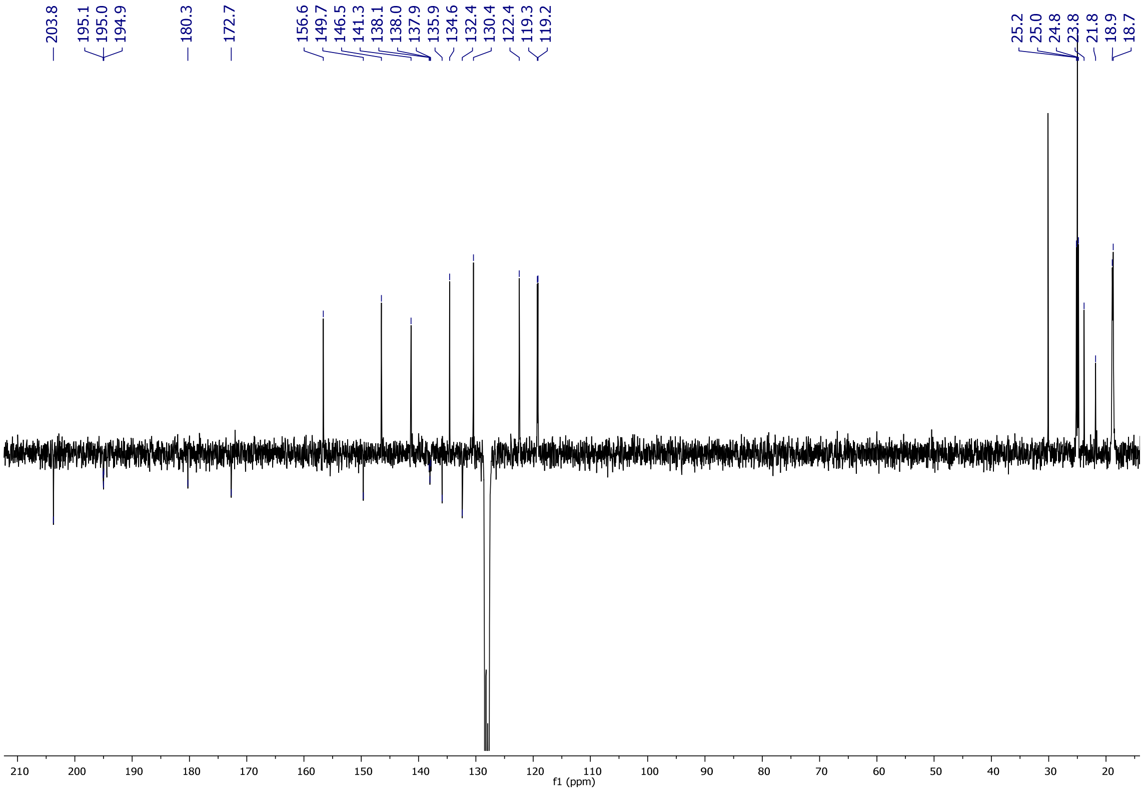

Figure S27. $^{13}C\{^{1}H\}$-APT NMR (75.5 MHz, C_6D_6, 298 K) of complex **7**.

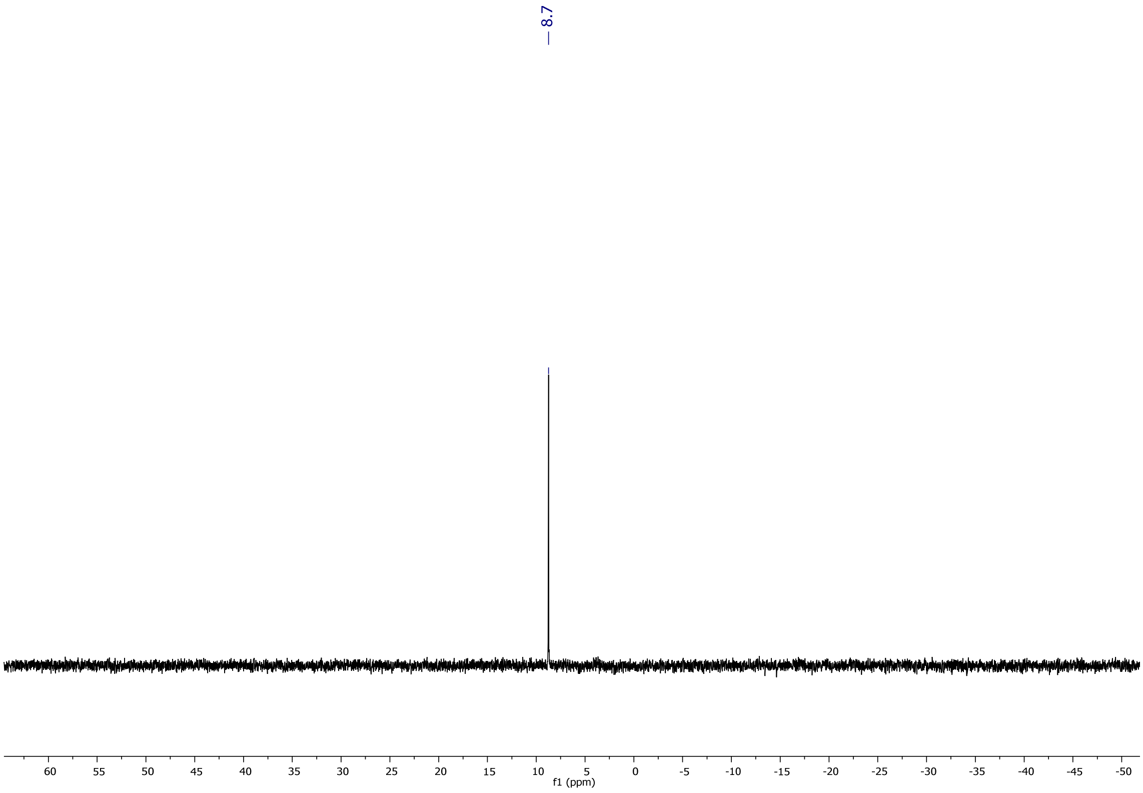

Figure S28. $^{31}P\{^{1}H\}$-NMR (121.5, MHz, C_6D_6, 298 K) of complex **7**.

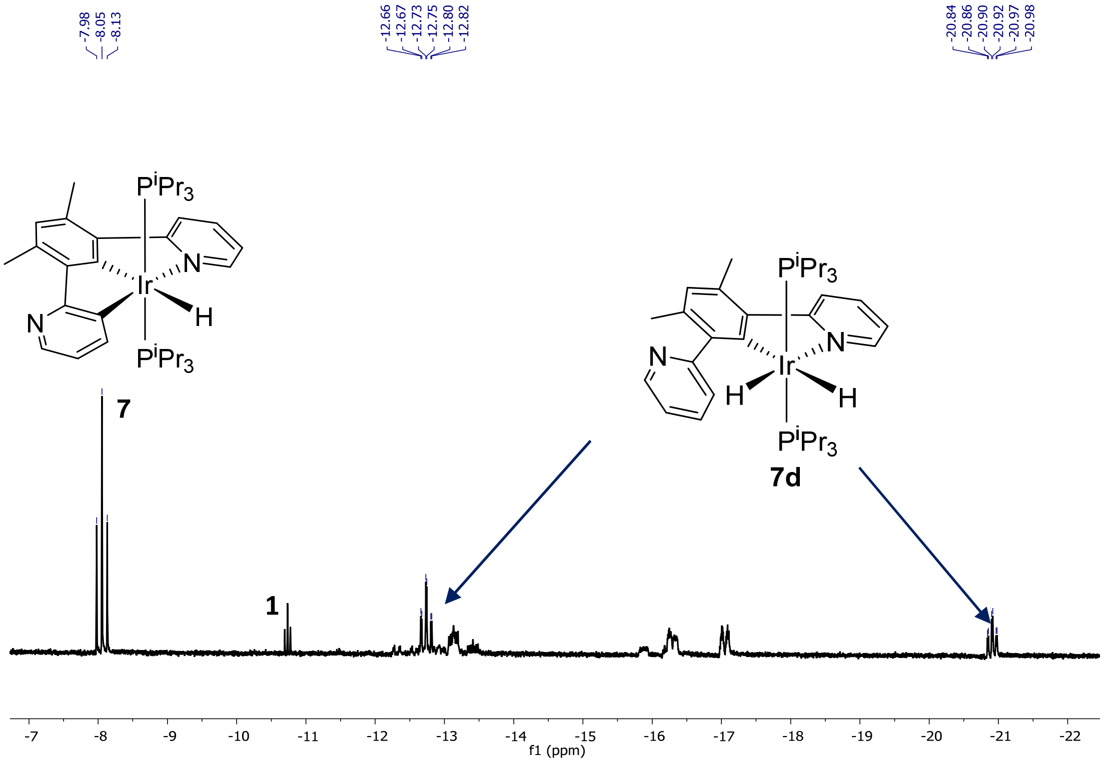

Figure S29. Hydride region of the ^{1}H NMR spectrum (300 MHz, toluene, 298 K) of the reaction's crude between complex **1** and 2-(1*H*-indol-2-yl)-6-phenyl-pyridine, after 3 h in toluene under reflux.

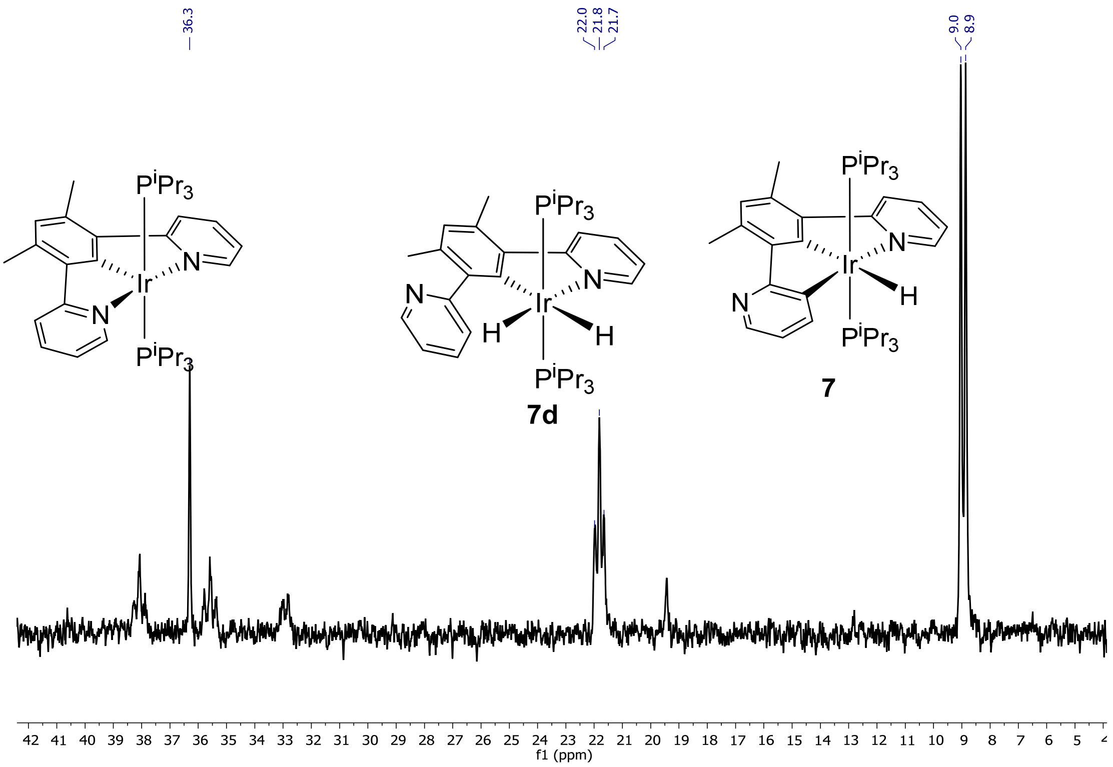

Figure S30. Off-resonance ^{31}P-NMR spectrum (121.49 MHz, toluene, 298 K) of the reaction's crude between complex **1** and 1,3-di(2-pyridyl)-4,6-dimethylbenzene, after 24 h in toluene under reflux.

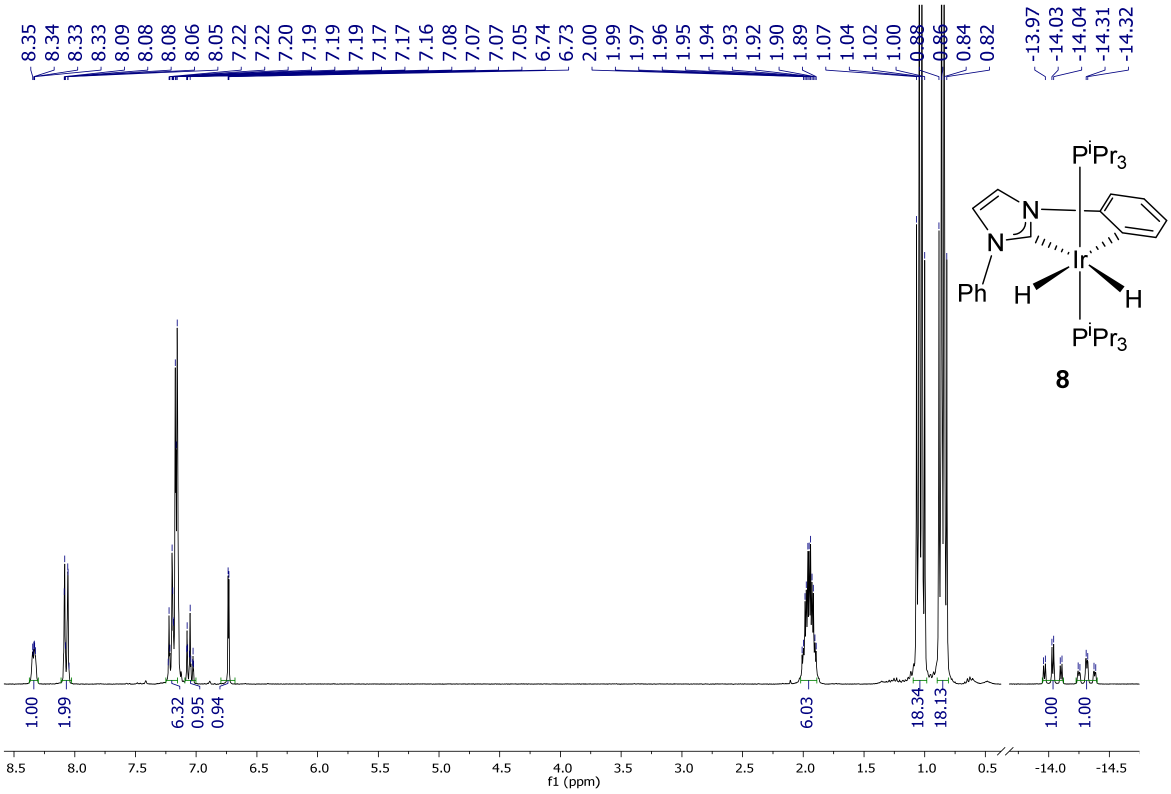

Figure S31. ^{1}H-NMR (300 MHz, C_6D_6, 298 K) of complex **8**.

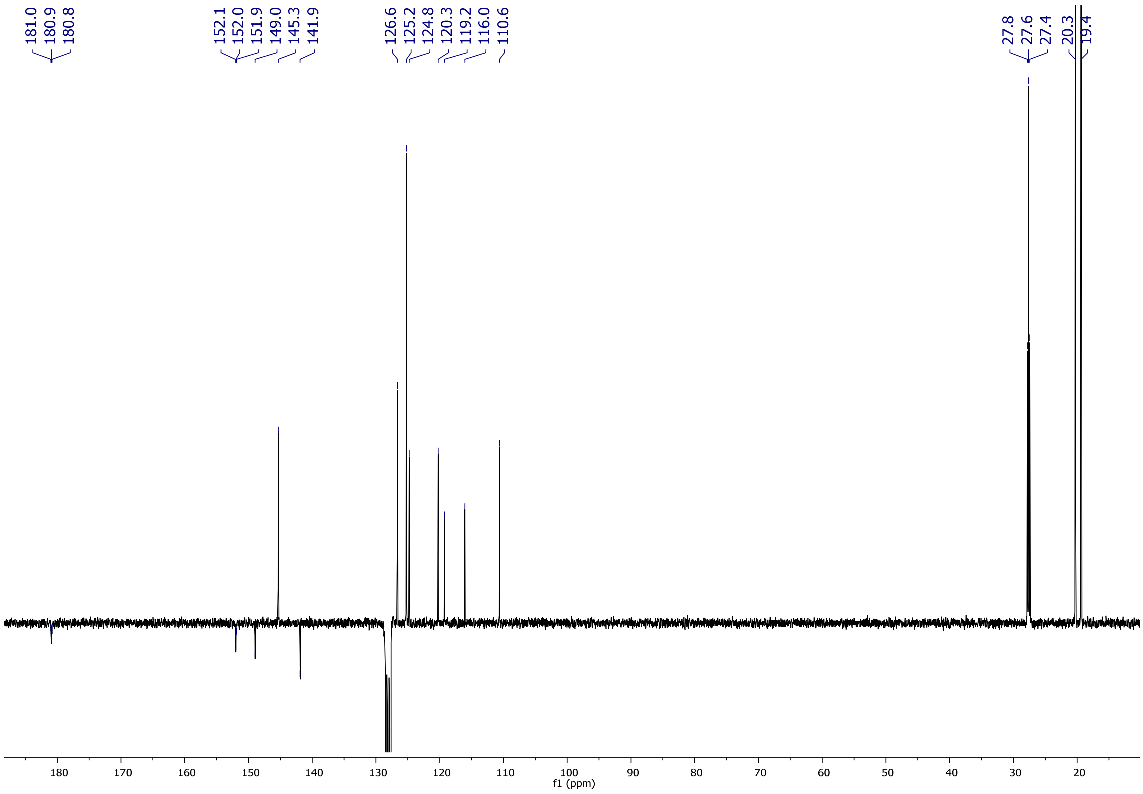

Figure S32. $^{13}C\{^{1}H\}$-APT NMR (75.5 MHz, C_6D_6, 298 K) of complex **8**.

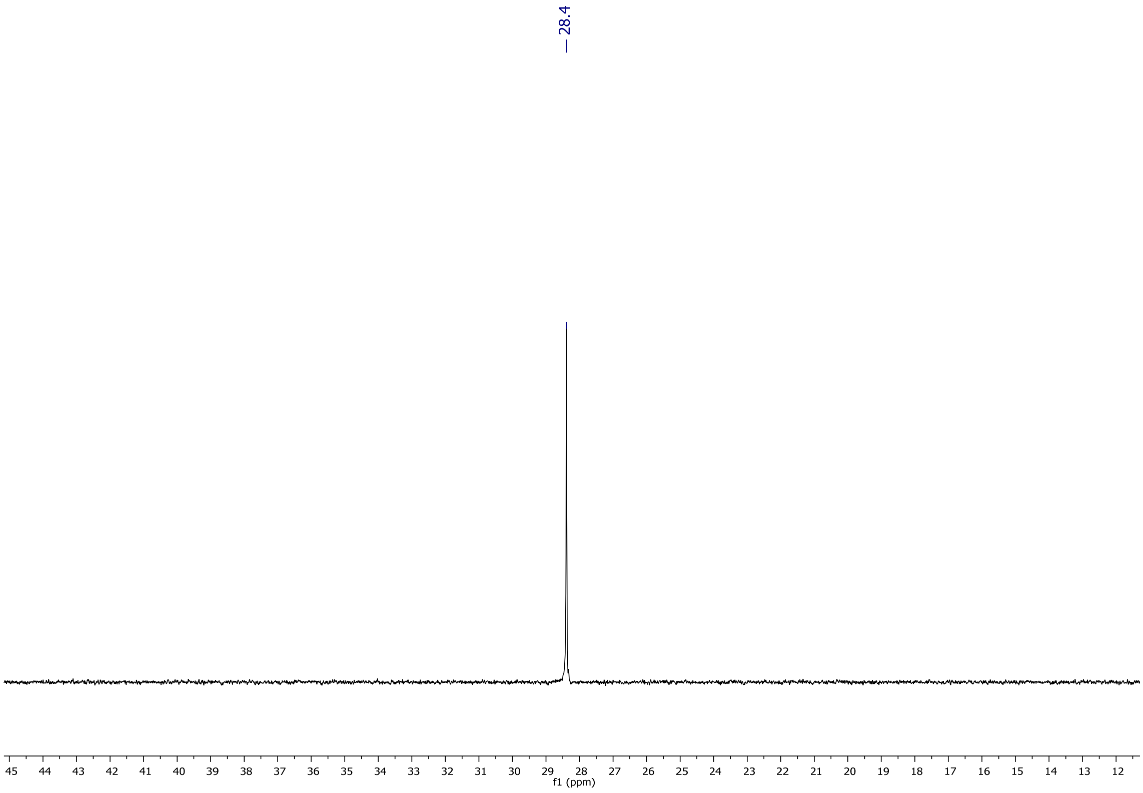

Figure S33. $^{31}P\{^{1}H\}$-NMR (121.5, MHz, C_6D_6, 298 K) of complex **8.**

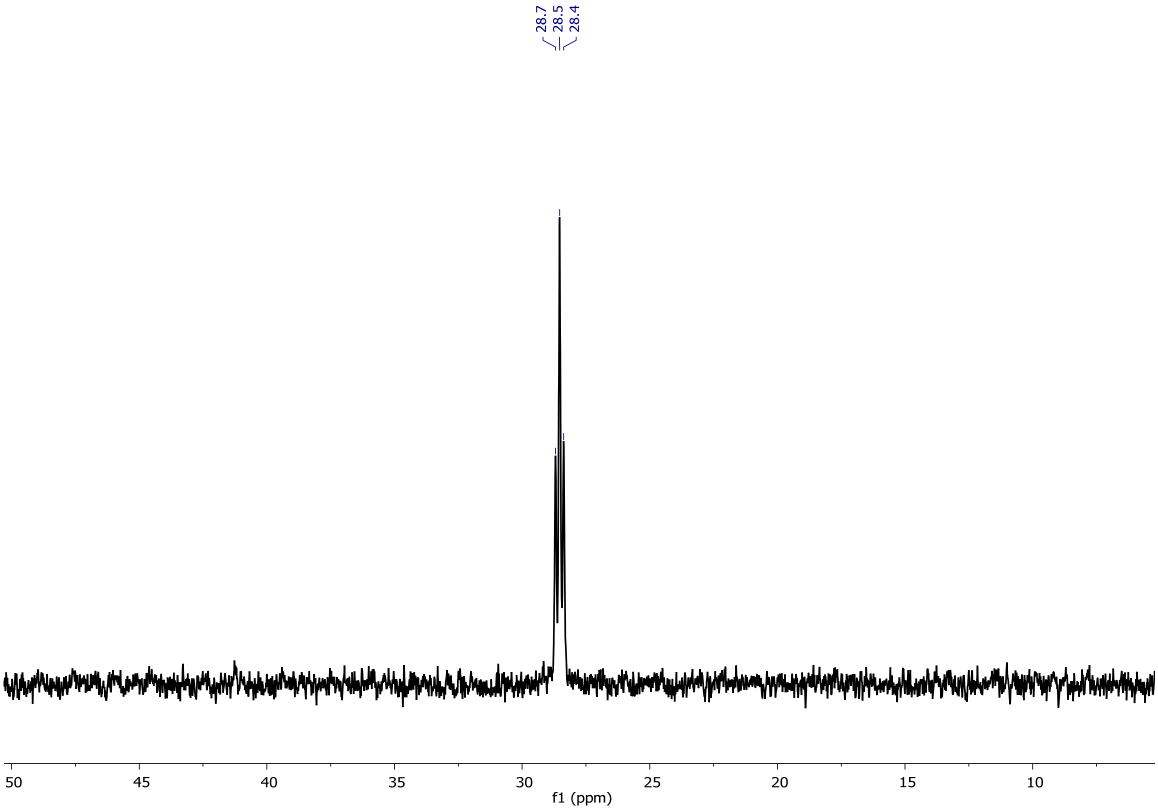

Figure S34. Off-resonance ^{31}P-NMR (121.5, MHz, C_6D_6, 298 K) of complex **8.**

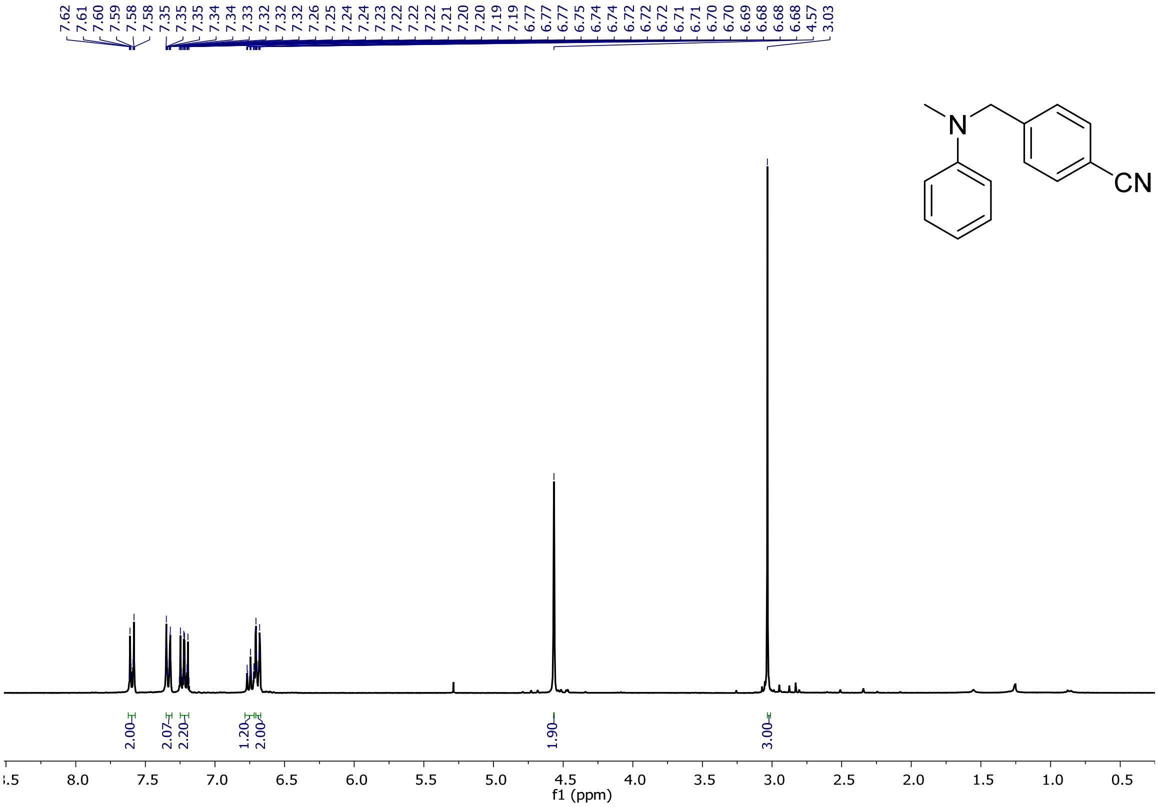

Figure S35. ^{1}H-NMR (300 MHz, C_6D_6, 298 K) of 4-((methyl(phenyl)amino)methyl)benzonitrile.

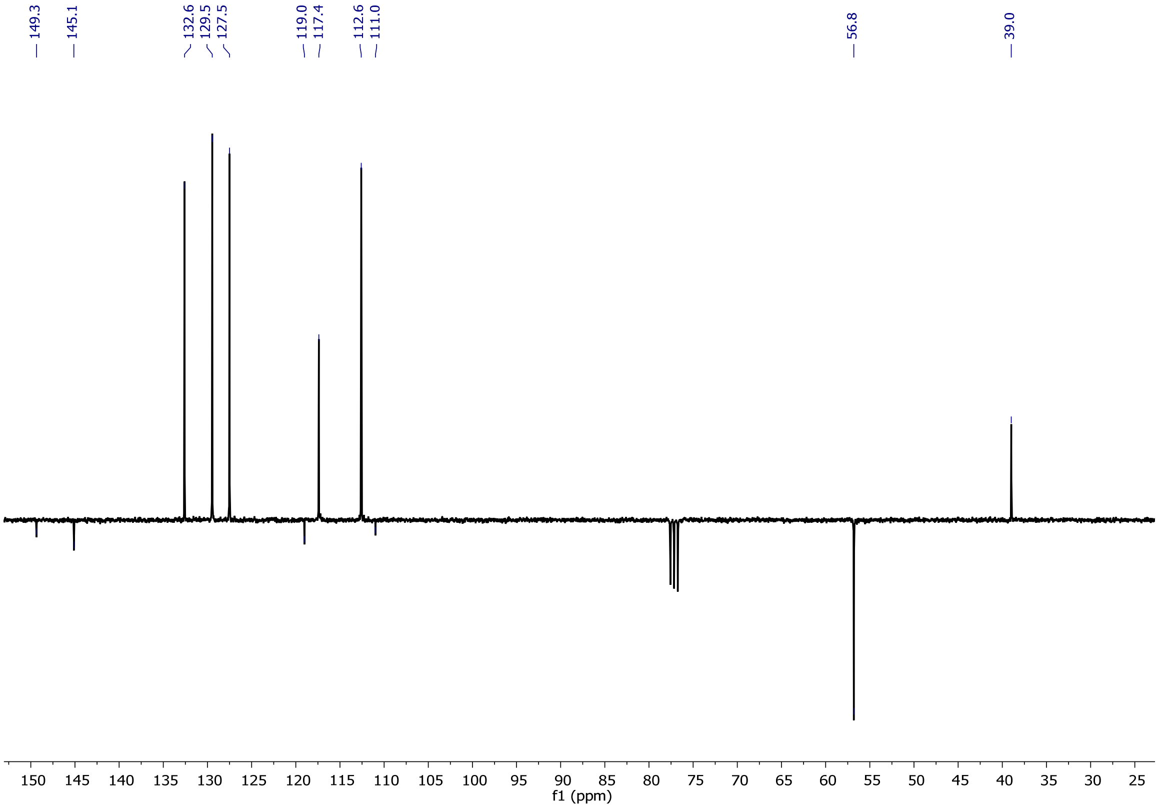

Figure S36. ^{13}C{^{1}H}-APT NMR (75.5 MHz, C_6D_6, 298 K) of 4-((methyl(phenyl)amino)methyl)benzonitrile.

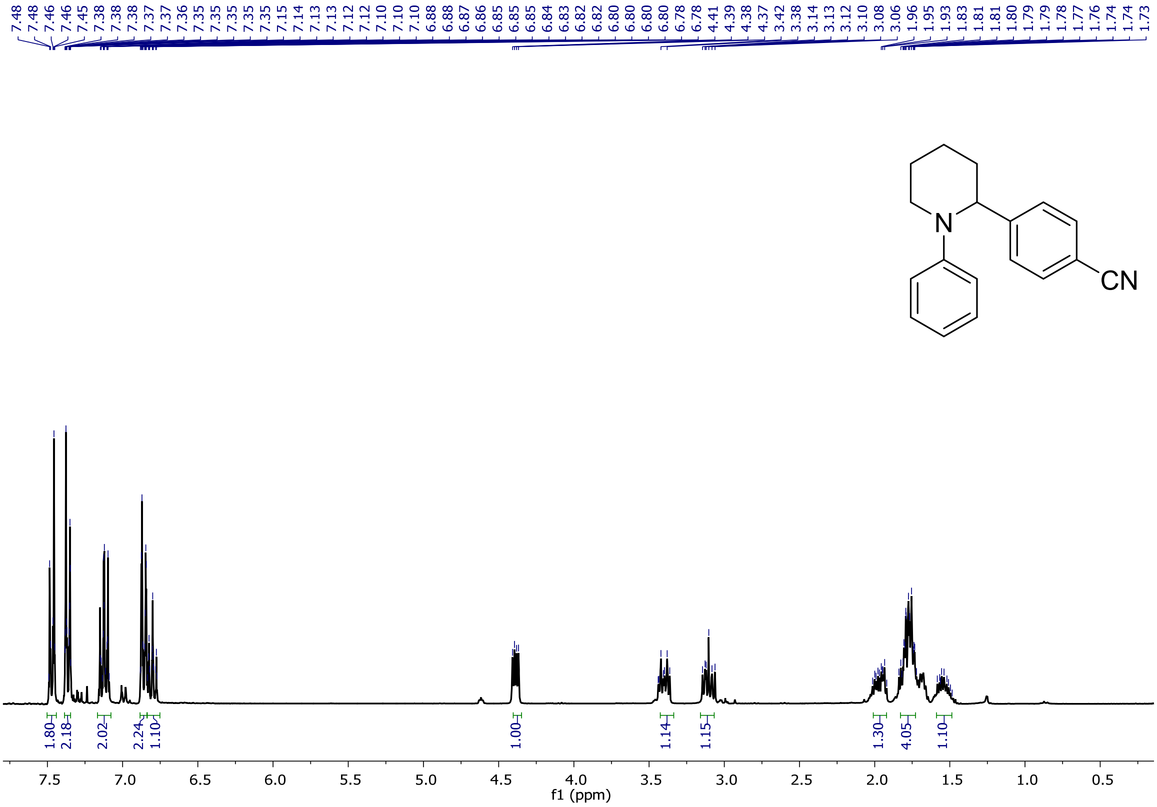

Figure S37. ^{1}H-NMR (300 MHz, C_6D_6, 298 K) of 4-(1-Phenylpiperidin-2-yl)benzonitrile.

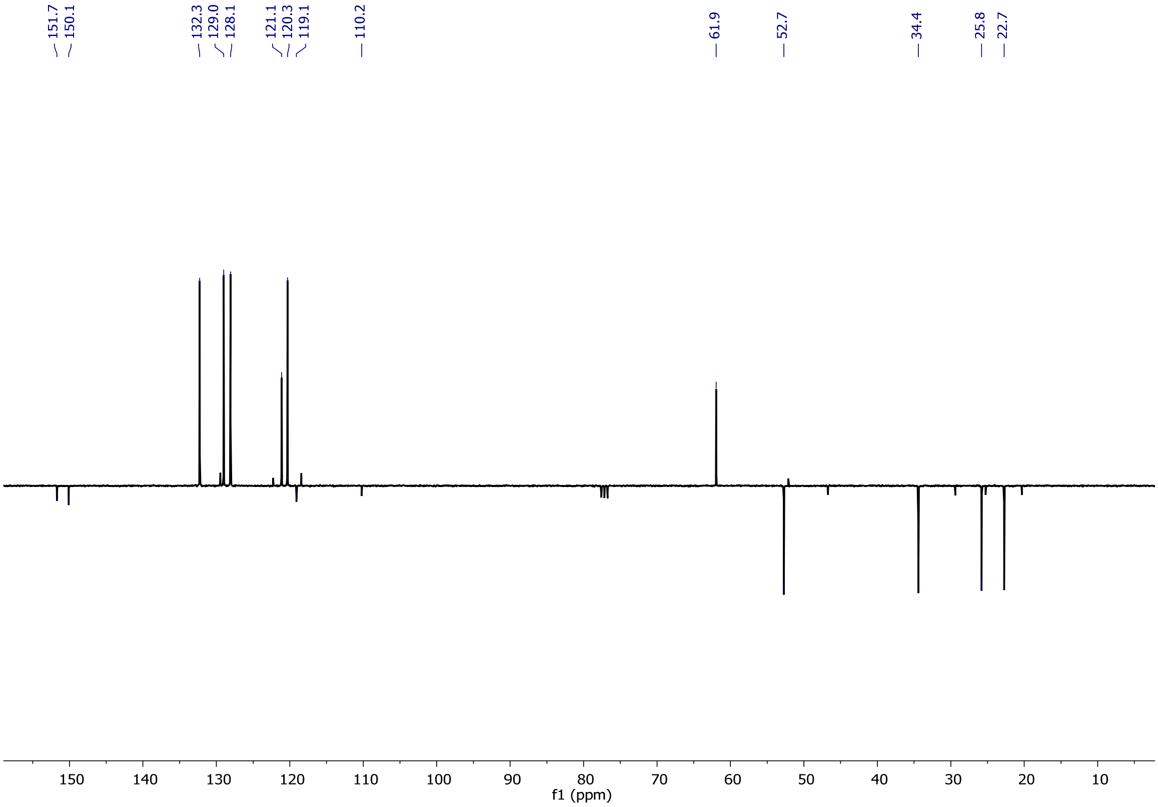

Figure S38. ^{13}C{^{1}H}-APT NMR (75.5 MHz, C_6D_6, 298 K) of 4-(1-Phenylpiperidin-2-yl)benzonitrile.

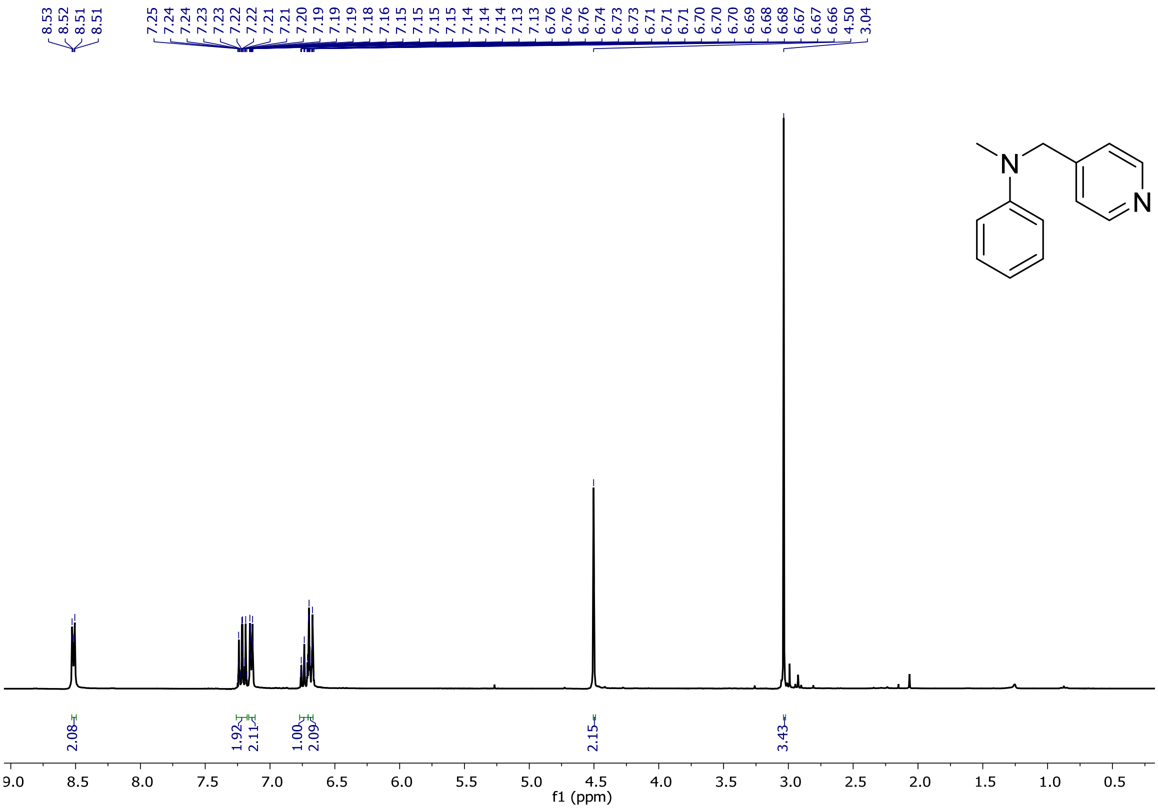

Figure S39. ¹H-NMR (300 MHz, C_6D_6, 298 K) of 4-((methyl(phenyl)amino)methyl)pyridine.

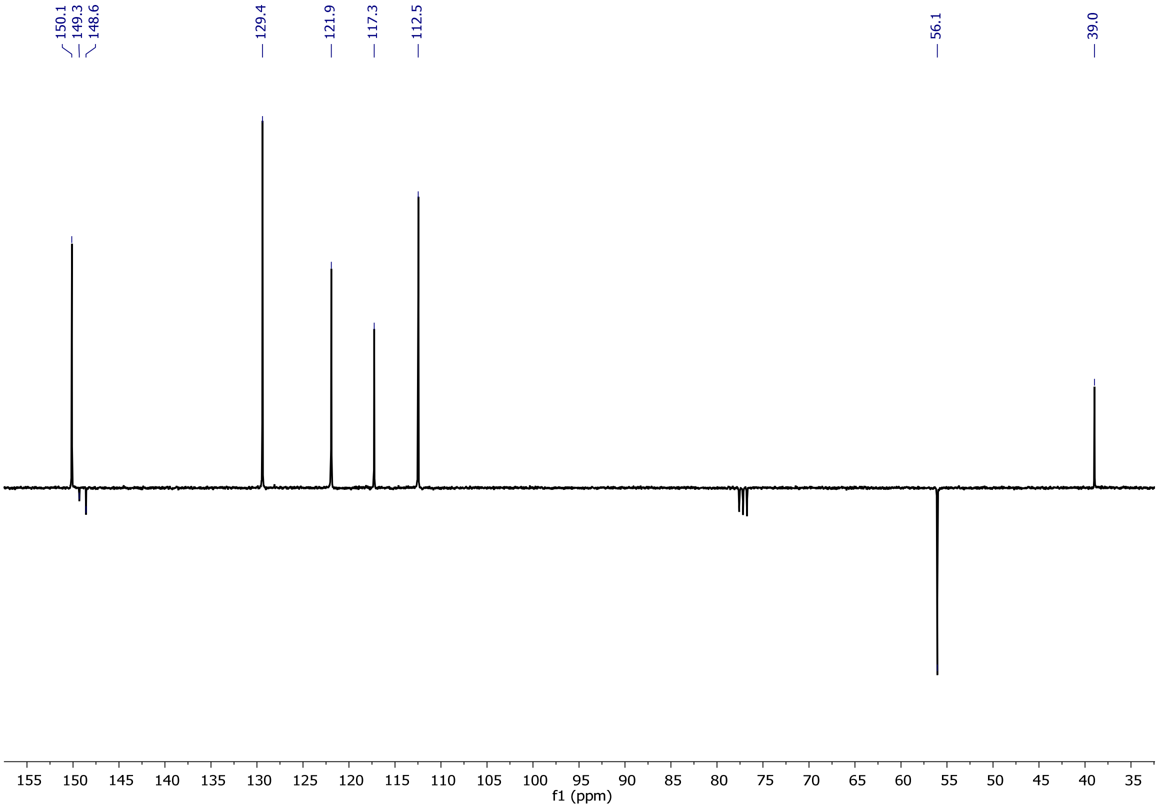

Figure S40. $^{13}C\{^{1}H\}$-APT NMR (75.5 MHz, C_6D_6, 298 K) of 4-((methyl(phenyl)amino)methyl)pyridine.

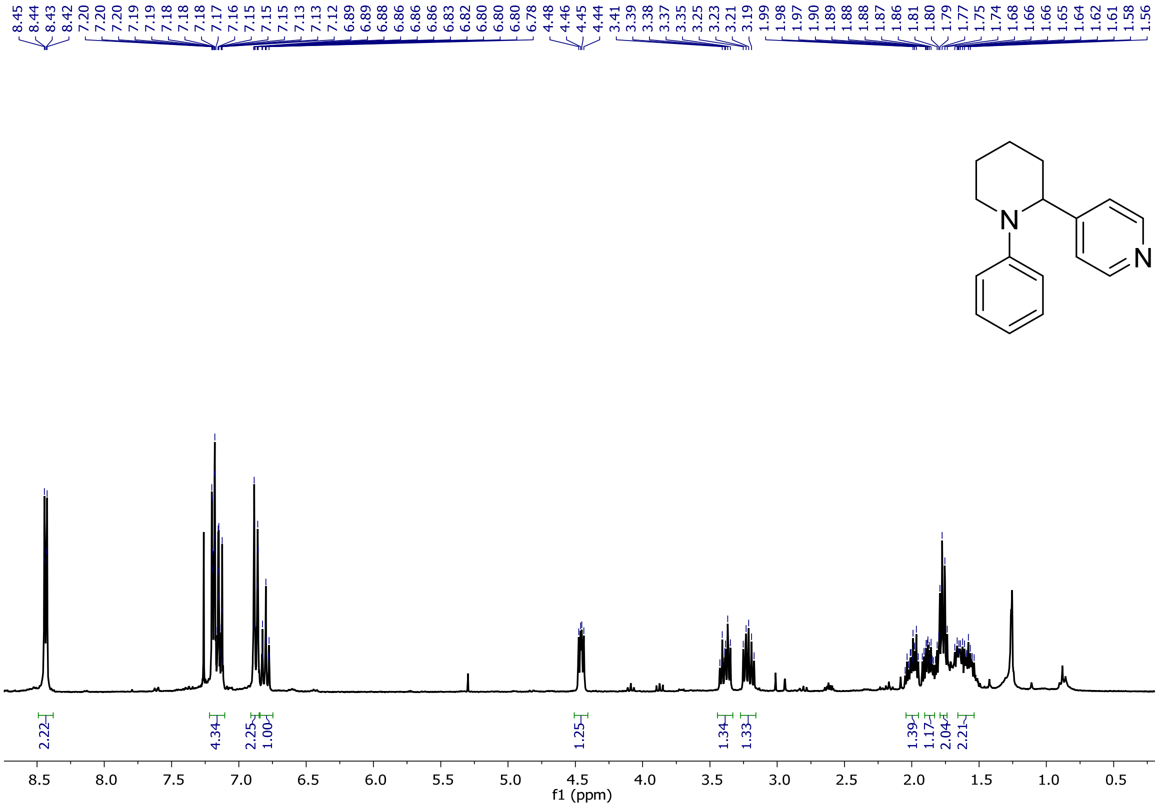

Figure S41. ^{1}H-NMR (300 MHz, C_6D_6, 298 K) of 4-(1-Phenylpiperidin-2-yl)pyridine.

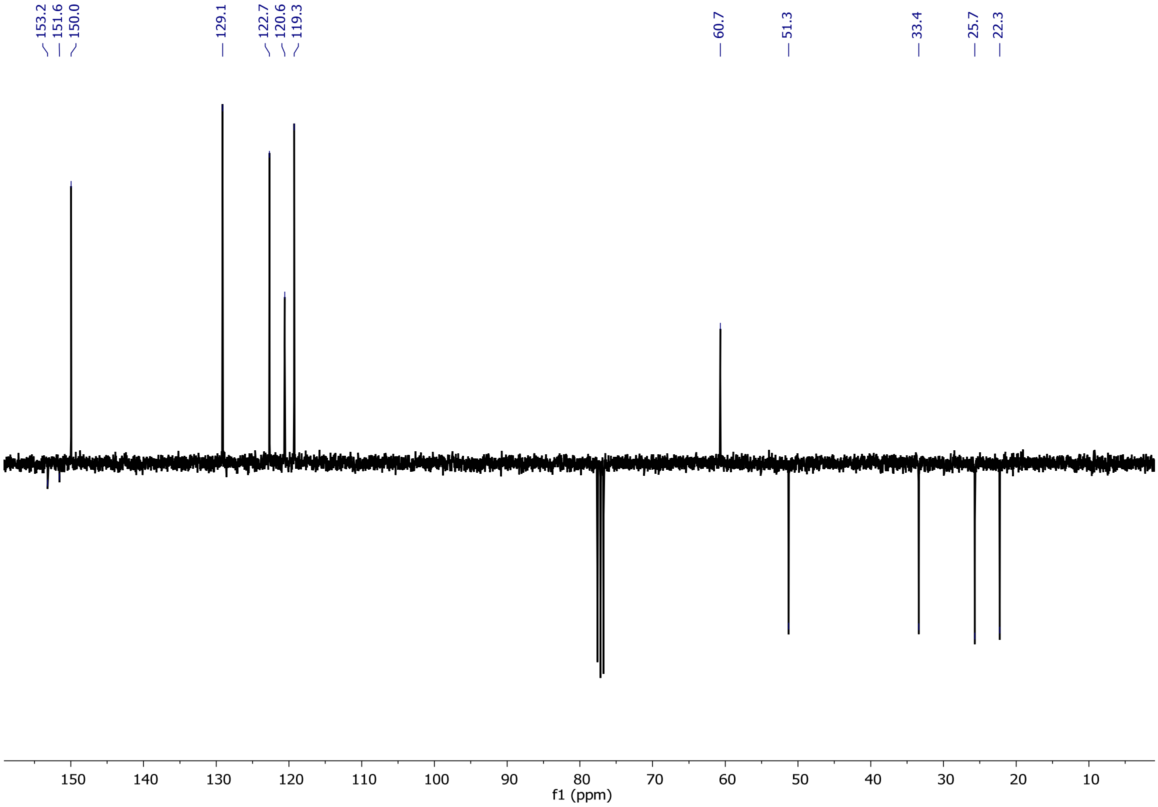

Figure S42. ^{13}C{^{1}H}-APT NMR (75.5 MHz, C_6D_6, 298 K) of 4-(1-Phenylpiperidin-2-yl)pyridine.

Structural Analysis of Complexes 2d, 3, 5, 7, and 8. X-ray data were collected on a D8 Venture Bruker diffractometer (Mo radiation, λ = 0.71073 Å). The crystals were mounted under oil in a MiTeGen mount and cooled to 100(2) K with an open-flow nitrogen gas (Oxford Cryosystems). Data were collected using φ and/or ω narrow scans. Diffracted intensities were integrated and corrected for absorption effects using SAINT[3] and SADABS[4] programs, included in APEX4 package. The structures were solved by direct methods and refined by full-matrix least squares on F^2 with SHELXL2019,[5] including isotropic and subsequently anisotropic displacement parameters. The hydrogen atoms were observed in the last Fourier Maps or calculated, and refined freely or using a restricted riding model.

Crystal data for **2d** (CCDC 2320385): $C_{39}H_{58}IrNOP_2$, Mw 811.00, red, irregular block, (0.250 x 0.170 x 0.150 mm^3), monoclinic, space group $P2_1/n$, *a*: 11.922(6) Å, *b*: 23.224(9) Å, *c*: 13.662(7) Å, *β*: 104.297(15)°, V = 3665(3) Å^3, Z = 4, Z' = 1, D_{calc}: 1.470 g cm^{-3}, F(000): 1656, T = 100(2) K, μ 3.760 mm^{-1}. 95948 measured reflections (2θ: 3-57°, ω and φ scans 0.5°), 9052 unique (R_{int} = 0.0439); min./max. transm. Factors 0.449/0.746. Final agreement factors were R^1 = 0.0220 (8412 observed reflections, I > 2σ(I)) and wR^2 = 0.0517; data/restraints/parameters 9052/3/415; GoF = 1.035. Largest peak and hole 1.740 (close to Ir atoms) and -0.554 e/ Å^3.

Crystal data for **3** (CCDC 2320383): $C_{36}H_{54}IrN_3P_2$, 1.4(CH_4O), Mw 827.82, yellow, irregular block (0.285 x 0.023 x 0.017 mm^3), monoclinic, space group $P2_1/n$, *a*: 14.5668(6) Å, *b*: 14.9771(7) Å, *c*: 17.8390(9) Å, *β*: 106.2897(17)°, V = 3735.7(3) Å^3, Z = 4, Z' = 1, D_{calc}: 1.472 g cm^{-3}, F(000): 1693, T = 100(2) K, μ 3.693 mm^{-1}. 141604 measured reflections (2θ: 3-57°, ω scans 0.3°), 9260 unique (R_{int} = 0.0440); min./max. transm. Factors 0.627/0.746. Final agreement factors were R^1 = 0.0282 (8371 observed reflections,

I > 2σ(I)) and wR^2 = 0.0741; data/restraints/parameters 9260/25/416; GoF = 1.075. Largest peak and hole 1.298 (close to Ir atoms) and -1.172 e/ Å^3.

Crystal data for **5** (CCDC 2320384): $C_{35}H_{54}IrNOP_2$, M_W 758.93, yellow, irregular block (0.150 x 0.100 x 0.070 mm^3), monoclinic, space group $P2_1/c$, *a*: 15.4093(5) Å, *b*: 13.8386(4) Å, *c*: 15.5255(5) Å, *β*: 100.6970(10)°, *V* = 3253.17(18) Å^3, *Z* = 4, *Z'* = 1, D_{calc}: 1.550 g cm^{-3}, F(000): 1544, T = 100(2) K, μ 4.231 mm^{-1}. 119983 measured reflections (2θ: 3-57°, ω scans 0.3°), 9933 unique (R_{int} = 0.0285); min./max. transm. Factors 0.651/0.746. Final agreement factors were R^1 = 0.0162 (9641 observed reflections, I > 2□(I)) and wR^2 = 0.0394; data/restraints/parameters 9933/0/376; GoF = 1.046. Largest peak and hole 1.767 (close to Ir atoms) and -0.871e/ Å^3.

Crystal data for **7** (CCDC 2320382): $C_{36}H_{57}IrN_2P_2$, M_W 771.97, yellow, irregular block, (0.141 x 0.119 x 0.067 mm^3), orthorhombic, space group $P2_12_12_1$, *a*: 10.7188(3) Å, *b*: 15.9592(5) Å, *c*: 19.7564(6) Å, *V* = 3379.60(18) Å^3, *Z* = 4, *Z'* = 1, D_{calc}: 1.517 g cm^{-3}, F(000): 1576, T = 100(2) K, μ 4.073 mm^{-1}. 62331 measured reflections (2θ: 3-57°, ω and φ scans 0.5°), 8383 unique (R_{int} = 0.0340); min./max. transm. Factors 0.665/0.862. Final agreement factors were R^1 = 0.0125 (8262 observed reflections, I > 2σ(I)) and wR^2 = 0.0276; Frack parameter -0.014(3); data/restraints/parameters 8383/0/388; GoF = 1.084. Largest peak and hole 0.389 (close to Ir atoms) and -0.468 e/ Å^3.

Crystal data for **8** (CCDC 2320381): $C_{33}H_{55}IrN_2P_2$, M_W 733.93, colourless, irregular block, (0. 0.217 x 0.195 x 0.161 mm^3), orthorhombic, space group $P2_12_12_1$, *a*: 13.0869(3) Å, *b*: 14.2483(4) Å, *c*: 17.8307(4) Å, *V* = 3324.82(14) Å^3, *Z* = 4, *Z'* = 1, D_{calc}: 1.466 g cm^{-3}, F(000): 1496, T = 100(2) K, μ 4.135 mm^{-1}. 59236 measured reflections (2θ: 3-57°, ω and φ scans 0.5°), 8141 unique (R_{int} = 0.0335); min./max. transm. Factors 0.613/0.746. Final agreement factors were R^1 = 0.0143 (8062 observed reflections, I > 2σ(I)) and wR^2 =

0.0352; Frack parameter 0.070(4); data/restraints/parameters 8141/26/360; GoF = 0.920. Largest peak and hole 0.539 (close to Ir atoms) and -1.468 e/ Å^3.

Computational Details. All calculations were performed at the DFT level using the B3LYP functional[6] supplemented with the Grimme's dispersion correction D3[7] as implemented in Gaussian09.[8] Ir atoms were described by means of an effective core potential SDD for the inner electron[9] and its associated double-ζ basis set for the outer ones, complemented with a set of f-polarization functions for iridium.[10] The 6-31G** basis set was used for the H, C, N, O and P atoms.[11] All minima were verified to have no negative frequencies. The geometries were fully optimized in THF (ε = 7.4257) solvent using the continuum SMD model.[12] We performed TD-DFT calculations at the same level of theory in THF calculating the lowest 50 singlet-singlet excitations at the ground state S_0. It should be noted that the singlet-triplet excitations are set to zero due to the neglect of spin-orbit coupling in the TDDFT calculations as implemented in G09. The UV/vis absorption spectra were obtained by using the GaussSum 3 software.[13] The phosphorescence emission compares well with the 0-0 transition calculated taking into account the zero point energies (zpe) of the geometries of both the optimized T1 and S_0 states in THF.

Energies of Optimized Structures of 2-7

Complex 2-S_0(THF)

Zero-point correction= 0.866374 (Hartree/Particle)
Thermal correction to Energy= 0.914359
Thermal correction to Enthalpy= 0.915304
Thermal correction to Gibbs Free Energy= 0.790229
Sum of electronic and zero-point Energies= -2436.475157
Sum of electronic and thermal Energies= -2436.427172
Sum of electronic and thermal Enthalpies= -2436.426227
Sum of electronic and thermal Free Energies= -2436.551302

Complex 2-T_1(THF)

Zero-point correction= 0.863425 (Hartree/Particle)
Thermal correction to Energy= 0.911474
Thermal correction to Enthalpy= 0.912419
Thermal correction to Gibbs Free Energy= 0.786918
Sum of electronic and zero-point Energies= -2436.405798
Sum of electronic and thermal Energies= -2436.357749
Sum of electronic and thermal Enthalpies= -2436.356804
Sum of electronic and thermal Free Energies= -2436.482305

Complex 3-S_0(THF)

Zero-point correction= 0.834375 (Hartree/Particle)
Thermal correction to Energy= 0.880008
Thermal correction to Enthalpy= 0.880952
Thermal correction to Gibbs Free Energy= 0.761478
Sum of electronic and zero-point Energies= -2355.315422
Sum of electronic and thermal Energies= -2355.269789
Sum of electronic and thermal Enthalpies= -2355.268845
Sum of electronic and thermal Free Energies= -2355.388319

Complex 3-T_1(THF)

Zero-point correction= 0.830208 (Hartree/Particle)
Thermal correction to Energy= 0.876529
Thermal correction to Enthalpy= 0.877473
Thermal correction to Gibbs Free Energy= 0.755244
Sum of electronic and zero-point Energies= -2355.229015
Sum of electronic and thermal Energies= -2355.182694
Sum of electronic and thermal Enthalpies= -2355.181750
Sum of electronic and thermal Free Energies= -2355.303979

Complex 4-S_0(THF)

Zero-point correction= 0.845237 (Hartree/Particle)
Thermal correction to Energy= 0.891167
Thermal correction to Enthalpy= 0.892111
Thermal correction to Gibbs Free Energy= 0.771239
Sum of electronic and zero-point Energies= -2339.249546
Sum of electronic and thermal Energies= -2339.203616
Sum of electronic and thermal Enthalpies= -2339.202671
Sum of electronic and thermal Free Energies= -2339.323544

Complex 4-T_1(THF)

Zero-point correction= 0.841299 (Hartree/Particle)
Thermal correction to Energy= 0.887712
Thermal correction to Enthalpy= 0.888656
Thermal correction to Gibbs Free Energy= 0.766089
Sum of electronic and zero-point Energies= -2339.173150
Sum of electronic and thermal Energies= -2339.126737
Sum of electronic and thermal Enthalpies= -2339.125793
Sum of electronic and thermal Free Energies= -2339.248360

Complex 5-S_0(THF)

Zero-point correction= 0.820079 (Hartree/Particle)
Thermal correction to Energy= 0.865299
Thermal correction to Enthalpy= 0.866243
Thermal correction to Gibbs Free Energy= 0.747052
Sum of electronic and zero-point Energies= -2282.912842
Sum of electronic and thermal Energies= -2282.867622
Sum of electronic and thermal Enthalpies= -2282.866678
Sum of electronic and thermal Free Energies= -2282.985869

Complex 5-T_1(THF)

Zero-point correction= 0.815331 (Hartree/Particle)
Thermal correction to Energy= 0.861496
Thermal correction to Enthalpy= 0.862440
Thermal correction to Gibbs Free Energy= 0.738892
Sum of electronic and zero-point Energies= -2282.830614
Sum of electronic and thermal Energies= -2282.784449
Sum of electronic and thermal Enthalpies= -2282.783505
Sum of electronic and thermal Free Energies= -2282.907053

Complex 6-S_0(THF)

Zero-point correction=	0.800556 (Hartree/Particle)
Thermal correction to Energy=	0.845318
Thermal correction to Enthalpy=	0.846262
Thermal correction to Gibbs Free Energy=	0.727484
Sum of electronic and zero-point Energies=	-2260.829297
Sum of electronic and thermal Energies=	-2260.784536
Sum of electronic and thermal Enthalpies=	-2260.783591
Sum of electronic and thermal Free Energies=	-2260.902370

Complex 7-S_0(THF)

Zero-point correction=	0.858426 (Hartree/Particle)
Thermal correction to Energy=	0.905647
Thermal correction to Enthalpy=	0.906591
Thermal correction to Gibbs Free Energy=	0.783388
Sum of electronic and zero-point Energies=	-2302.298480
Sum of electronic and thermal Energies=	-2302.251259
Sum of electronic and thermal Enthalpies=	-2302.250315
Sum of electronic and thermal Free Energies=	-2302.373518

Complex 7-T_1(THF)

Zero-point correction=	0.853256 (Hartree/Particle)
Thermal correction to Energy=	0.901311
Thermal correction to Enthalpy=	0.902256
Thermal correction to Gibbs Free Energy=	0.774666
Sum of electronic and zero-point Energies=	-2302.211419
Sum of electronic and thermal Energies=	-2302.163363
Sum of electronic and thermal Enthalpies=	-2302.162419
Sum of electronic and thermal Free Energies=	-2302.290009

UV-vis Spectra of Complexes 2-7 (Observed and Calculated)

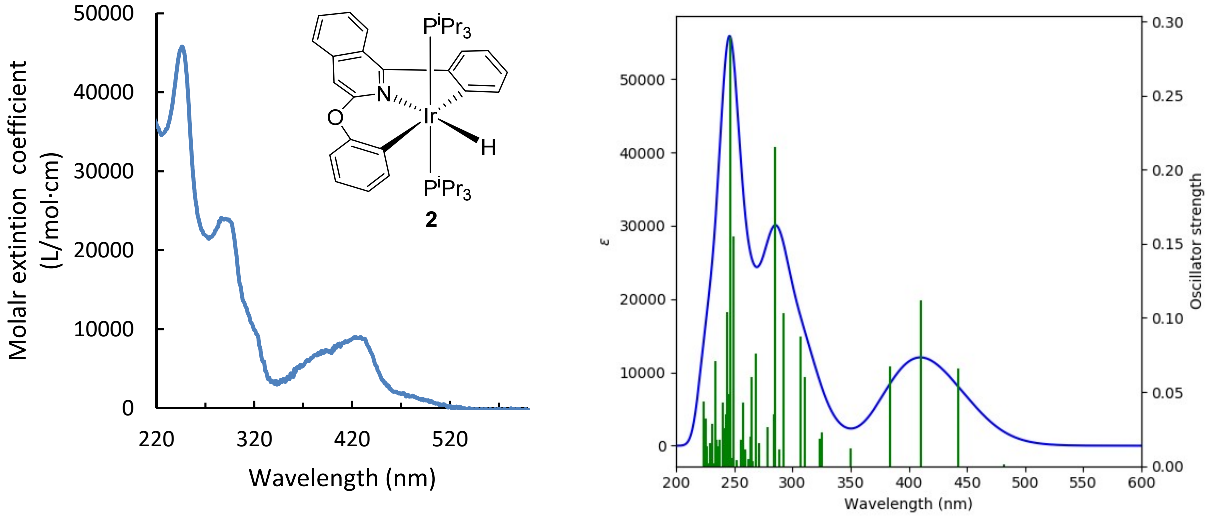

Figure S43. Observed UV-vis spectrum of complex **2** in 2-MeTHF (1.0 x 10^{-5} M) and calculated (B3LYP-D3//SDD(f)/6-31G**) in THF.

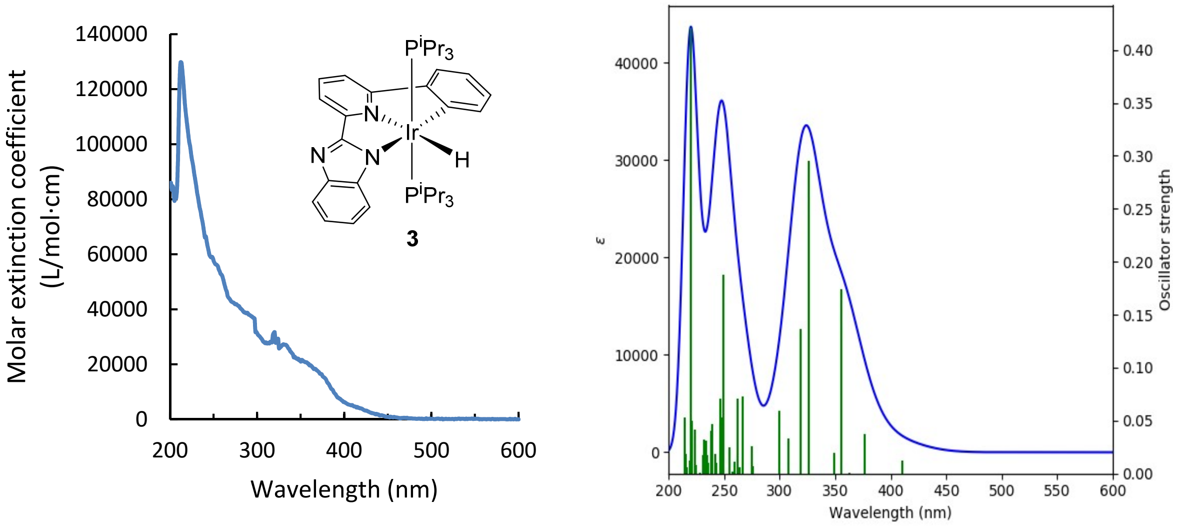

Figure S44. Observed UV-vis spectrum of complex **3** in 2-MeTHF (1.0 x 10^{-5} M) and calculated (B3LYP-D3//SDD(f)/6-31G**) in THF.

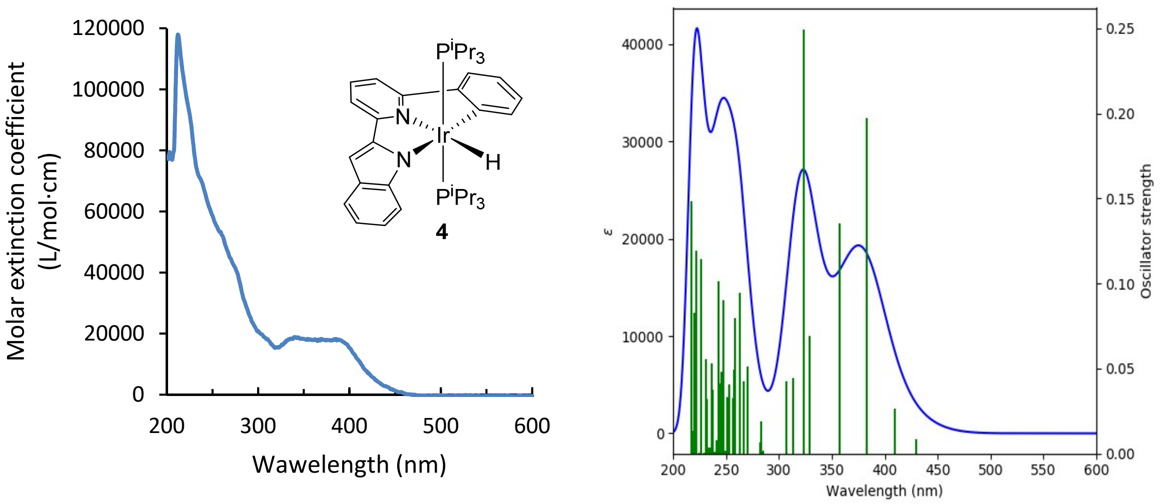

Figure S45. Observed UV-vis spectrum of complex **4** in 2-MeTHF (1.0 x 10^{-5} M) and calculated (B3LYP-D3//SDD(f)/6-31G**) in THF.

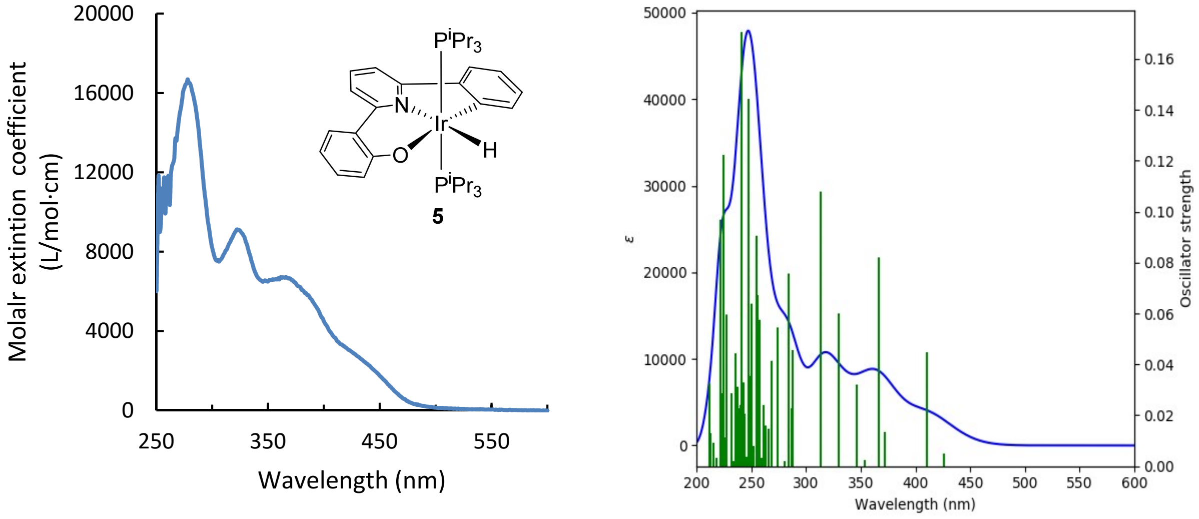

Figure S46. Observed UV-vis spectrum of complex **5** in 2-MeTHF (1.0 x 10^{-4} M) and calculated (B3LYP-D3//SDD(f)/6-31G**) in THF.

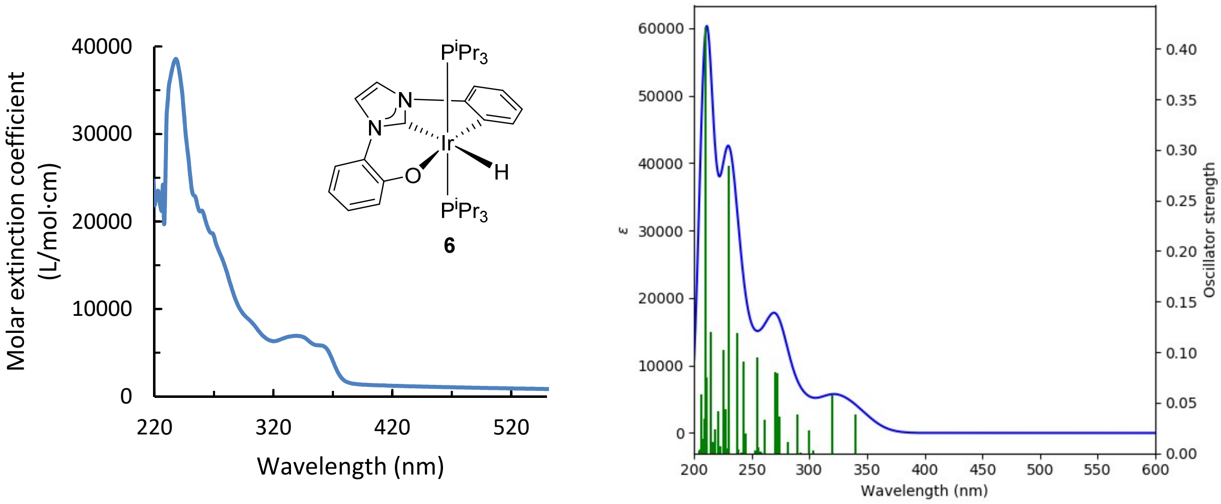

Figure S47. Observed UV-vis spectrum of complex **6** in 2-MeTHF (1.0 x 10^{-5} M) and calculated (B3LYP-D3//SDD(f)/6-31G**) in THF.

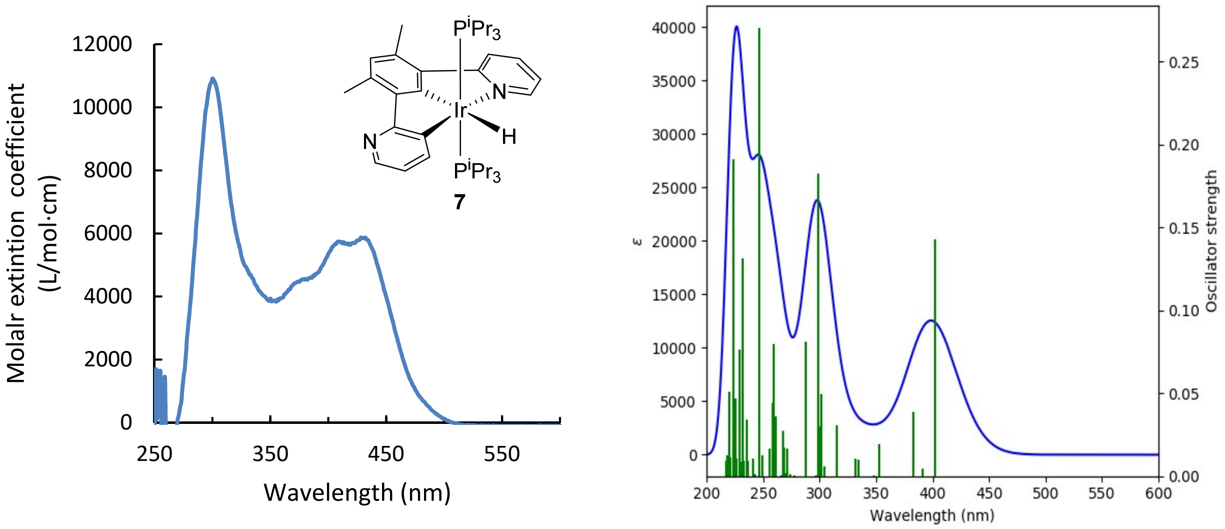

Figure S48. Observed UV-vis spectrum of complex **7** in 2-MeTHF (1.0 x 10^{-4} M) and calculated (B3LYP-D3//SDD(f)/6-31G**) in THF.

Analysis of Computed UV/Vis Data of Complexes 2-7

Selected transitions for the calculated UV spectra, oscillator strengths, and molecular orbital contributions to the transitions are given in Tables S1 – S6.

Table S1. Selected transitions for the calculated UV spectrum of complex 2 in THF

λ (nm)	Osc. Strength	Symmetry	Major contributions	Minor contributions
581	0	Triplet-A	H-1->LUMO (52%), H-3->LUMO (19%), H-2->LUMO (19%)	H-4->LUMO (7%), HOMO->L+1 (3%)
509	0	Triplet-A	HOMO->LUMO (91%)	H-1->LUMO (3%)
481	0.0011	Singlet-A	HOMO->LUMO (96%)	H-1->LUMO (2%)
443	0.0659	Singlet-A	H-1->LUMO (74%), H-2->LUMO (23%)	-
411	0.1121	Singlet-A	H-2->LUMO (54%), H-3->LUMO (25%), H-1->LUMO (18%)	-
384	0.0673	Singlet-A	H-3->LUMO (71%), H-2->LUMO (21%)	H-1->LUMO (4%)
307	0.0873	Singlet-A	H-1->L+1 (67%), H-6->LUMO (21%)	H-2->L+1 (8%)
293	0.103	Singlet-A	H-2->L+1 (78%)	HOMO->-L+2 (6%), H-11->LUMO (4%), H-6->LUMO (2%), H-1->L+1 (3%)
298	0.0564	Singlet-A	H-5->LUMO (93%)	-
285	0.2153	Singlet-A	H-3->L+1 (58%), HOMO->L+2 (22%)	H-1->L+2 (7%), H-11->LUMO (4%), H-6->LUMO (3%), H-2->L+1 (2%)
284	0.0348	Singlet-A	H-7->LUMO (76%), H-1->L+2 (13%)	H-2->L+1 (2%)
247	0.2895	Singlet-A	H-5->L+1 (25%), H-2->L+4 (12%), H-1->L+4 (10%), H-11->LUMO (10%)	H-4->L+1 (9%), HOMO->L+5 (8%), H-3->L+3 (3%), H-3->L+4 (3%), H-1->L+7 (3%), H-2->L+3 (2%)

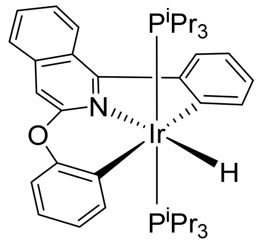

2

Table S2. Selected transitions for the calculated UV spectrum of complex 3 in THF

λ (nm)	Osc. Strength	Symmetry	Major contributions	Minor contributions
464	0	Triplet-A	HOMO->LUMO (58%), HOMO->L+1 (27%)	H-4->LUMO (3%)
433	0	Triplet-A	H-1->LUMO (52%), HOMO->L+1 (25%), HOMO->LUMO (11%)	H-3->L+1 (2%)
411	0.0122	Singlet-A	HOMO->LUMO (88%)	HOMO->L+1 (9%)
377	0.0377	Singlet-A	HOMO->L+1 (80%)	H-3->LUMO (2%), H-1->LUMO (5%), H-1->L+1 (3%), HOMO->LUMO (7%)
326	0.2957	Singlet-A	H-3->LUMO (68%), H-3->L+1 (20%)	H-2->LUMO (2%), H-1->LUMO (2%), H-1->L+1 (3%)
300	0.0592	Singlet-A	H-4->L+1 (90%)	H-1->L+1 (3%)
274	0.0253	Singlet-A	H-5->LUMO (91%)	H-4->L+1 (2%)
267	0.0726	Singlet-A	H-5->L+1 (57%), HOMO->L+3 (31%)	

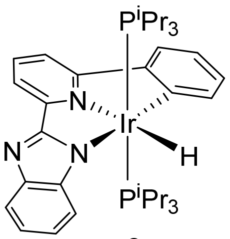

3

Table S3. Selected transitions for the calculated UV spectrum of complex 4 in THF

λ (nm)	Osc. Strength	Symmetry	Major contributions	Minor contributions
535	0	Triplet-A	HOMO->LUMO (44%), HOMO->L+1 (40%)	H-1->LUMO (2%), H-1->L+1 (6%)
442	0	Triplet-A	H-1->LUMO (55%), HOMO->LUMO (25%)	HOMO->L+1 (8%), H-1->L+1 (3%), H-3->L+1 (2%)
430	0.0088	Singlet-A	HOMO->LUMO (90%)	H-1->LUMO (7%)
409	0.0267	Singlet-A	HOMO->L+1 (93%)	H-1->LUMO (4%)
383	0.1974	Singlet-A	H-1->LUMO (85%)	HOMO->LUMO (8%), HOMO->L+1 (3%)
357	0.1351	Singlet-A	H-1->L+1 (84%)	H-4->LUMO (2%), H-3->LUMO (7%), H-3->L+1 (5%)
323	0.2495	Singlet-A	H-3->LUMO (37%), H-3->L+1 (49%)	H-1->L+1 (9%)
313	0.0446	Singlet-A	H-4->LUMO (89%)	H-4->L+1 (3%), H-1->L+1 (2%)
270	0.0513	Singlet-A	H-5->LUMO (85%)	H-5->L+1 (4%), HOMO->L+3 (2%)

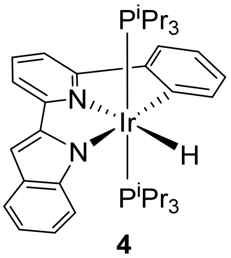

4

Table S4. Selected transitions for the calculated UV spectrum of complex 5 in THF

λ (nm)	Osc. Strength	Symmetry	Major contributions	Minor contributions
491	0	Triplet-A	HOMO->LUMO (15%), HOMO->L+1 (67%)	H-3->LUMO (2%), H-1->L+1 (6%)
426	0.0048	Singlet-A	HOMO->LUMO (97%)	-
410	0.0447	Singlet-A	HOMO->L+1 (95%)	H-1->LUMO (3%)
366	0.0819	Singlet-A	H-1->LUMO (67%), H-2->LUMO (19%),	H-3->L+1 (3%), H-2->L+1 (5%)
330	0.0601	Singlet-A	H-3->LUMO (93%)	-
313	0.1077	Singlet-A	H-3->L+1 (84%)	H-4->LUMO (5%), H-1->LUMO (4%)
284	0.0756	Singlet-A	H-4->LUMO (19%), HOMO->L+3 (16%), HOMO->L+5 (33%)	H-4->L+1 (4%), H-2->L+5 (3%), H-1->L+5 (4%), HOMO->L+2 (2%), HOMO->L+6 (3%), HOMO->L+7 (6%)
274	0.0545	Singlet-A	H-4->LUMO (14%), H-4->L+1 (70%)	H-3->L+1 (2%), HOMO->L+3 (7%)

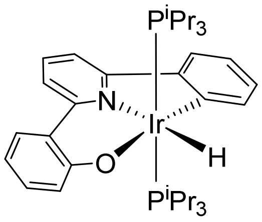

5

Table S5. Selected transitions for the calculated UV spectrum of complex 6 in THF

λ (nm)	Osc. Strength	Symmetry	Major contributions	Minor contributions
401	0	Triplet-A	HOMO->LUMO (65%), HOMO->L+1 (25%)	HOMO->L+5 (3%)
340	0.0382	Singlet-A	HOMO->LUMO (95%)	HOMO->L+1 (3%)
320	0.0577	Singlet-A	HOMO->L+1 (95%)	-
290	0.0381	Singlet-A	H-2->LUMO (80%)	H-1->LUMO (3%), H-1->L+4 (7%)
272	0.0797	Singlet-A	H-3->LUMO (33%), H-2->L+1 (26%), H-1->L+4 (18%)	HOMO->L+3 (6%), HOMO->L+5 (6%)
270	0.0802	Singlet-A	H-2->L+1 (62%), H-3->LUMO (10%), H-1->L+4 (11%)	H-2->LUMO (5%), H-1->L+1 (3%), HOMO->L+3 (3%)
230	0.2844	Singlet-A	H-4->LUMO (53%), H-6->LUMO (10%)	H-5->L+1 (3%), H-4->L+1 (2%), H-3->L+3 (4%), H-2->L+3 (6%), H-2->L+5 (4%), HOMO->L+7 (7%), HOMO->L+8 (2%)

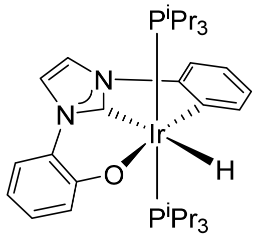

6

Table S6. Selected transitions for the calculated UV spectrum of complex 7 in THF

λ (nm)	Osc. Strength	Symmetry	Major contributions	Minor contributions
472	0	Triplet-A	H-1->LUMO (11%), HOMO->LUMO (65%)	H-4->LUMO (4%), H-4->L+1 (2%), H-1->L+1 (2%), HOMO->L+1 (5%), HOMO->L+2 (4%)
402	0.1429	Singlet-A	H-1->LUMO (13%), HOMO->LUMO (85%)	-
383	0.0384	Singlet-A	H-2->LUMO (11%), H-1->LUMO (73%), HOMO->LUMO (11%)	-
352	0.0191	Singlet-A	HOMO->L+1 (92%)	H-1->L+1 (5%)
301	0.0494	Singlet-A	H-3->L+1 (24%), H-1->L+2 (61%)	H-6->LUMO (4%), H-4->LUMO (2%)
299	0.1822	Singlet-A	H-4->LUMO (66%)	H-1->L+2 (11%), H-2->L+2 (8%), H-1->L+1 (3%)
288	0.0808	Singlet-A	H-6->LUMO (83%)	H-4->LUMO (5%), H-1->L+2 (5%)
270	0.0017	Singlet-A	H-5->L+1 (48%), H-5->L+2 (26%)	H-5->LUMO (9%), H-3->L+2 (7%), H-2->L+4 (4%)

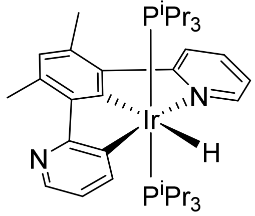

7

Theoretical Analysis of Molecular Orbitals of Complexes 2-7

Energies and population analysis (%) of molecular orbitals are given in Tables S7–S12 whereas Figures S7–S12 collects the frontier molecular orbitals.

Table S7. Composition (%) of the molecular orbitals of complex 2

MO	eV	Iridium	P^iPr_3 (1)	P^iPr_3 (2)	H	L (Ph)	L (Isoqui)	L (PhO)
L+9	1.39	13	4	5	0	32	39	6
L+8	1.28	49	11	11	0	9	9	11
L+7	0.95	42	15	15	4	9	5	12
L+6	0.86	23	4	5	0	9	9	50
L+5	0.61	3	2	1	0	24	6	64
L+4	0.38	7	2	2	0	50	1	39
L+3	0.22	88	3	4	0	3	1	1
L+2	-0.05	3	1	1	0	35	58	3
L+1	-0.47	1	0	1	0	4	85	10
LUMO	-1.67	2	1	1	0	20	76	0
HOMO	-4.92	37	3	4	0	11	4	40
H-1	-5.11	20	4	3	0	27	27	18
H-2	-5.36	46	1	2	0	15	18	18
H-3	-5.48	47	9	10	0	12	21	1
H-4	-5.97	27	3	3	0	21	3	43
H-5	-6.06	12	4	3	0	23	3	55
H-6	-6.29	17	3	3	0	41	10	25
H-7	-6.68	6	3	3	0	38	40	9
H-8	-6.85	17	27	28	0	5	6	17
H-9	-6.99	28	13	10	1	28	3	17

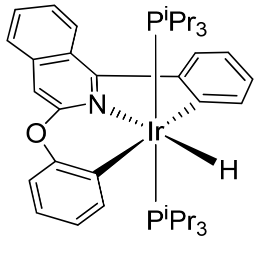

2

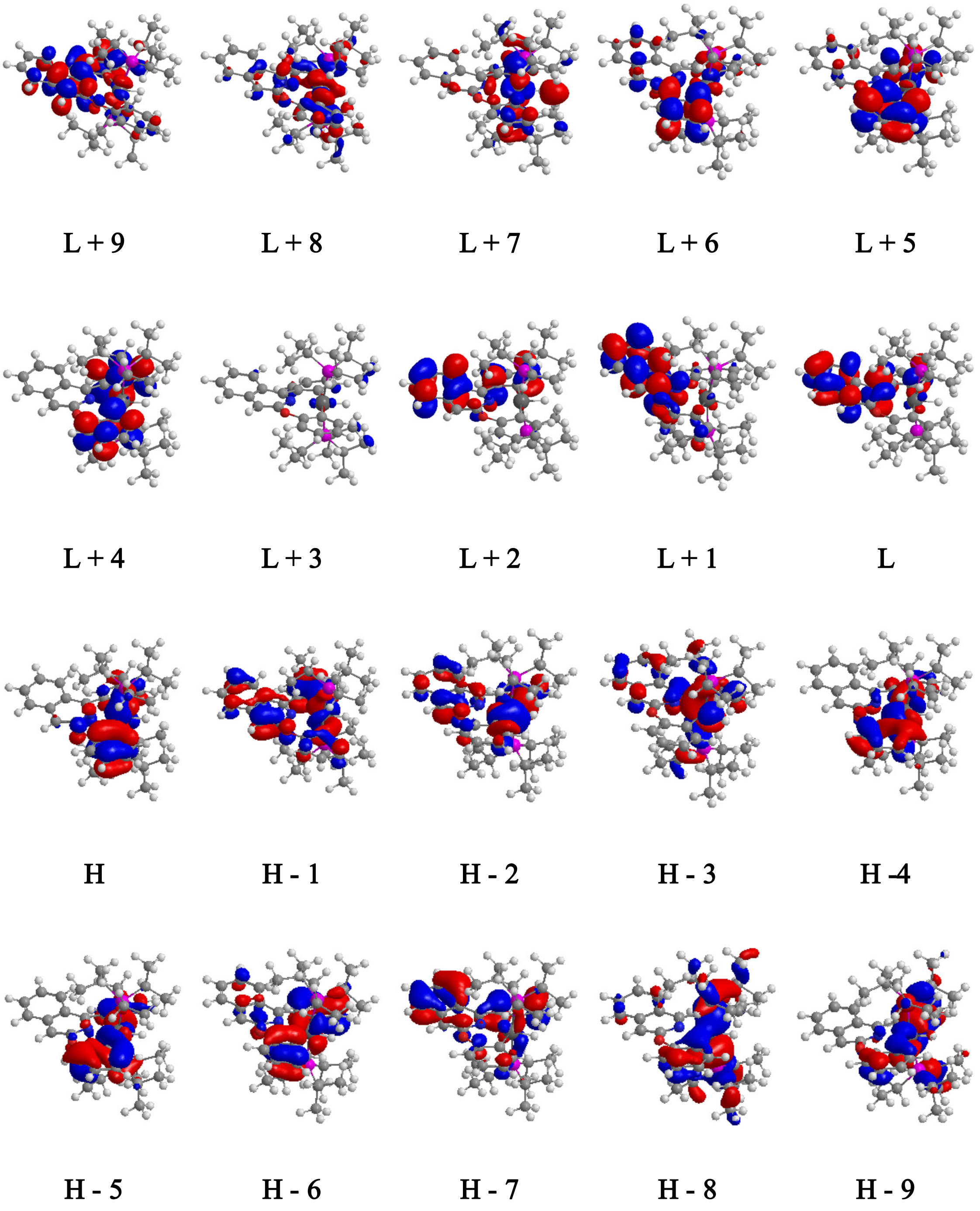

Figure S49. Molecular orbitals of complex **2** (isovalue 0.003 au).

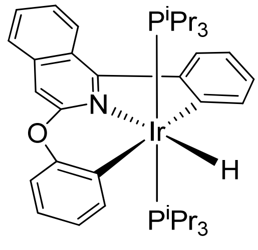

2

Table S8. Composition (%) of the molecular orbitals of complex 3

MO	eV	Iridium	P^{i}Pr$_3$ (1)	P^{i}Pr$_3$ (2)	H	L (Ph)	L (Py)	L (NBzim)
L+9	1.61	93	4	7	2	0	0	-3
L+8	1.1	1	1	1	0	1	1	95
L+7	0.98	54	15	15	2	5	6	4
L+6	0.96	10	2	1	0	35	24	27
L+5	0.73	38	23	23	1	9	0	5
L+4	0.53	5	1	1	0	62	12	19
L+3	0.31	4	1	1	0	41	14	40
L+2	0.21	88	5	6	0	1	0	0
L+1	-1.13	1	1	1	0	33	58	7
LUMO	-1.24	3	1	1	0	4	63	27
HOMO	-5.01	30	2	2	0	18	7	41
H-1	-5.3	17	7	7	0	6	11	51
H-2	-5.55	76	2	1	0	10	2	9
H-3	-5.58	18	7	8	0	19	9	39
H-4	-5.86	23	3	3	0	19	11	41
H-5	-6.32	7	3	3	0	57	15	15
H-6	-6.77	31	22	22	0	12	2	11
H-7	-6.98	5	1	0	0	11	1	82
H-8	-7.08	21	19	19	0	33	2	6
H-9	-7.11	18	10	9	32	2	29	1

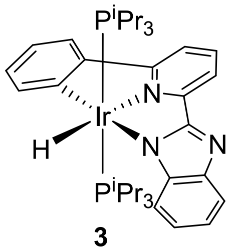

3

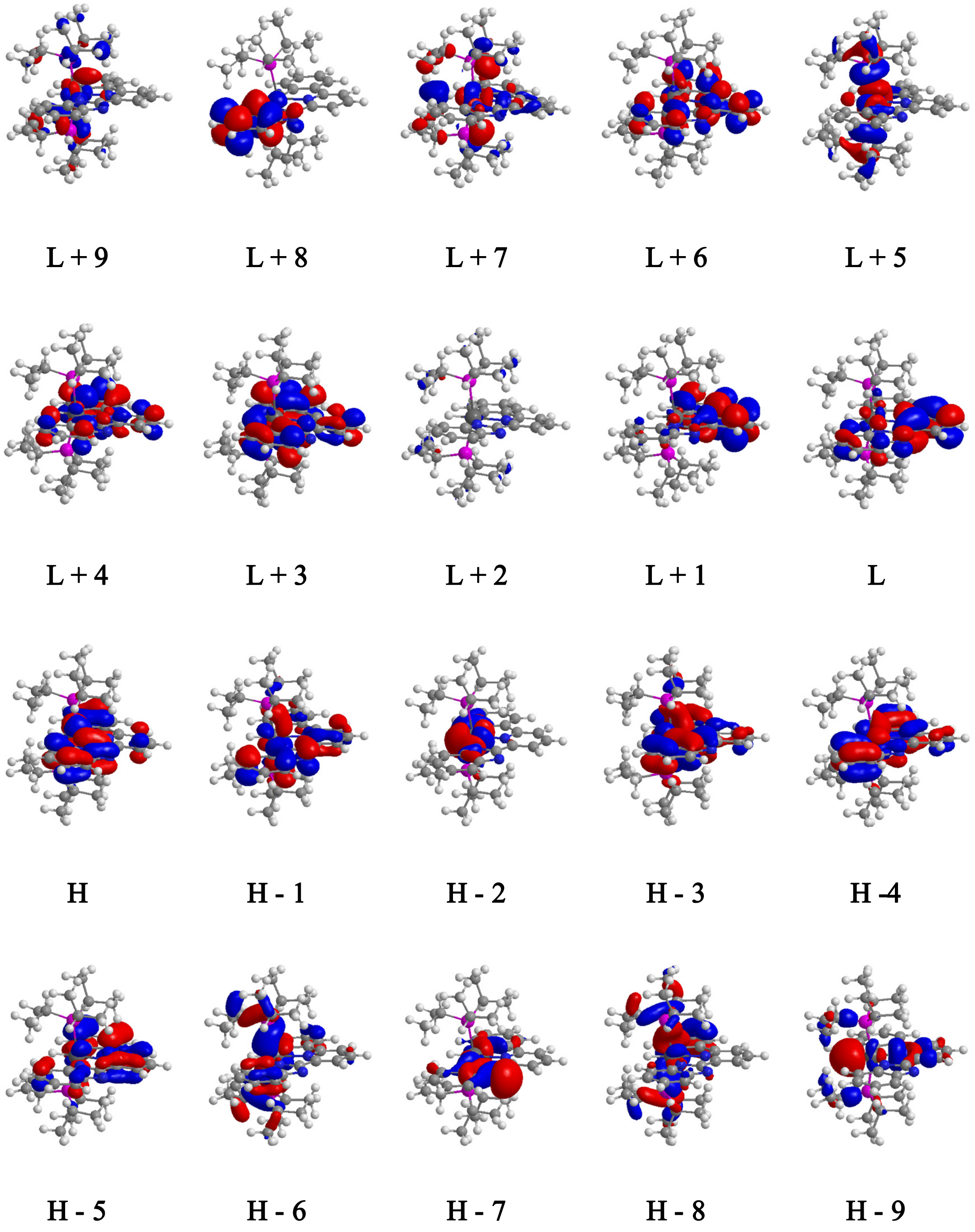

Figure S50. Molecular orbitals of complex **3** (isovalue 0.003 au).

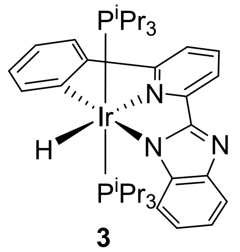

3

Table S9. Composition (%) of the molecular orbitals of complex 4

MO	eV	Iridium	P^iPr_3 (1)	P^iPr_3 (2)	H	L (Ph)	L (Py)	L (Ind)
L+9	1.66	92	5	5	2	-1	0	-1
L+8	1.28	2	1	1	0	1	0	95
L+7	1.07	7	0	0	0	35	26	31
L+6	1.02	61	15	15	1	2	4	1
L+5	0.82	38	23	23	2	9	0	5
L+4	0.61	4	1	1	0	54	17	24
L+3	0.39	4	1	1	0	53	11	31
L+2	0.22	89	6	6	0	0	0	0
L+1	-1.04	1	1	1	0	20	56	22
LUMO	-1.1	3	2	2	0	17	64	13
HOMO	-4.64	17	3	3	0	3	1	73
H-1	-5	13	2	2	0	14	19	51
H-2	-5.45	77	1	2	0	10	1	9
H-3	-5.47	24	11	11	0	25	7	23
H-4	-5.71	32	5	5	0	17	12	30
H-5	-6.25	7	3	3	0	57	17	13
H-6	-6.68	28	19	19	0	16	3	17
H-7	-6.99	16	20	20	0	22	2	21
H-8	-7.02	17	9	9	32	1	30	1
H-9	-7.21	23	2	5	0	43	1	26

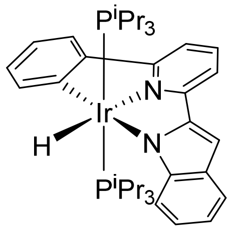

4

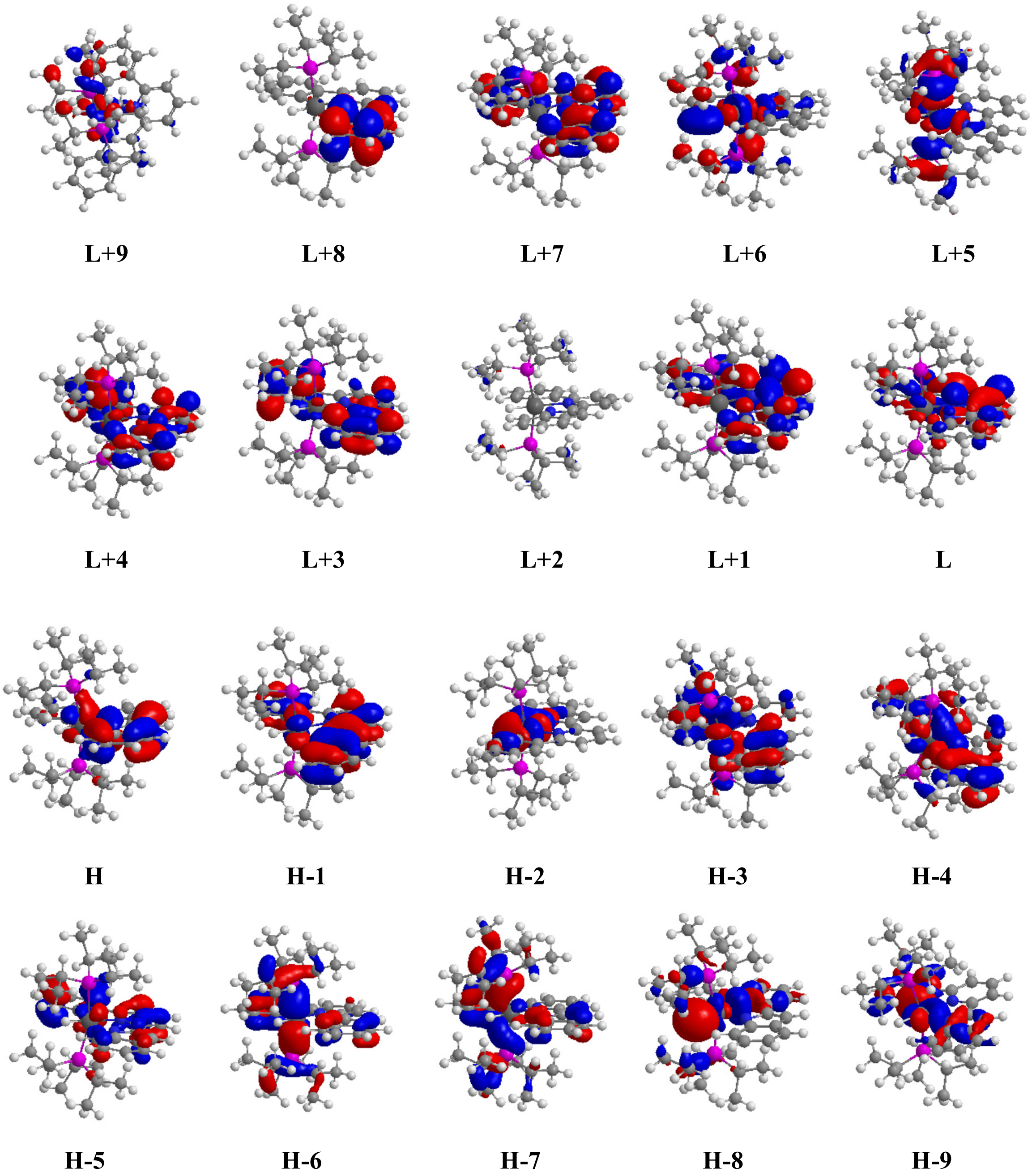

Figure S51. Molecular orbitals of complex **4** (isovalue 0.003 au).

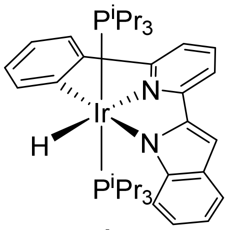

4

Table S10. Composition (%) of the molecular orbitals of complex 5

MO	eV	Iridium	P^{i}Pr$_3$ (1)	P^{i}Pr$_3$ (2)	H	L (Ph)	L (py)	L (PhO)
L+9	1.57	99	0	5	0	-1	0	0
L+8	1.44	9	3	2	0	25	27	34
L+7	1.13	38	8	8	0	17	9	20
L+6	0.92	44	12	10	2	7	6	19
L+5	0.81	26	17	15	1	17	5	19
L+4	0.6	3	1	2	0	44	6	44
L+3	0.38	7	1	1	0	48	15	29
L+2	0.21	91	4	4	0	1	0	1
L+1	-0.96	1	1	0	0	4	61	33
LUMO	-1.07	2	1	1	0	31	62	1
HOMO	-4.66	21	3	3	0	4	5	63
H-1	-5.13	31	7	7	0	38	12	6
H-2	-5.26	61	1	1	0	7	2	27
H-3	-5.56	45	11	11	0	7	15	11
H-4	-5.98	6	2	1	0	34	20	36
H-5	-6.43	5	6	5	0	31	7	46
H-6	-6.68	29	14	13	0	24	3	17
H-7	-6.81	26	6	10	4	12	8	34
H-8	-7.01	22	19	13	7	27	9	2
H-9	-7.09	17	9	15	17	19	17	5

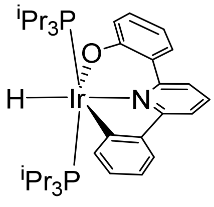

5

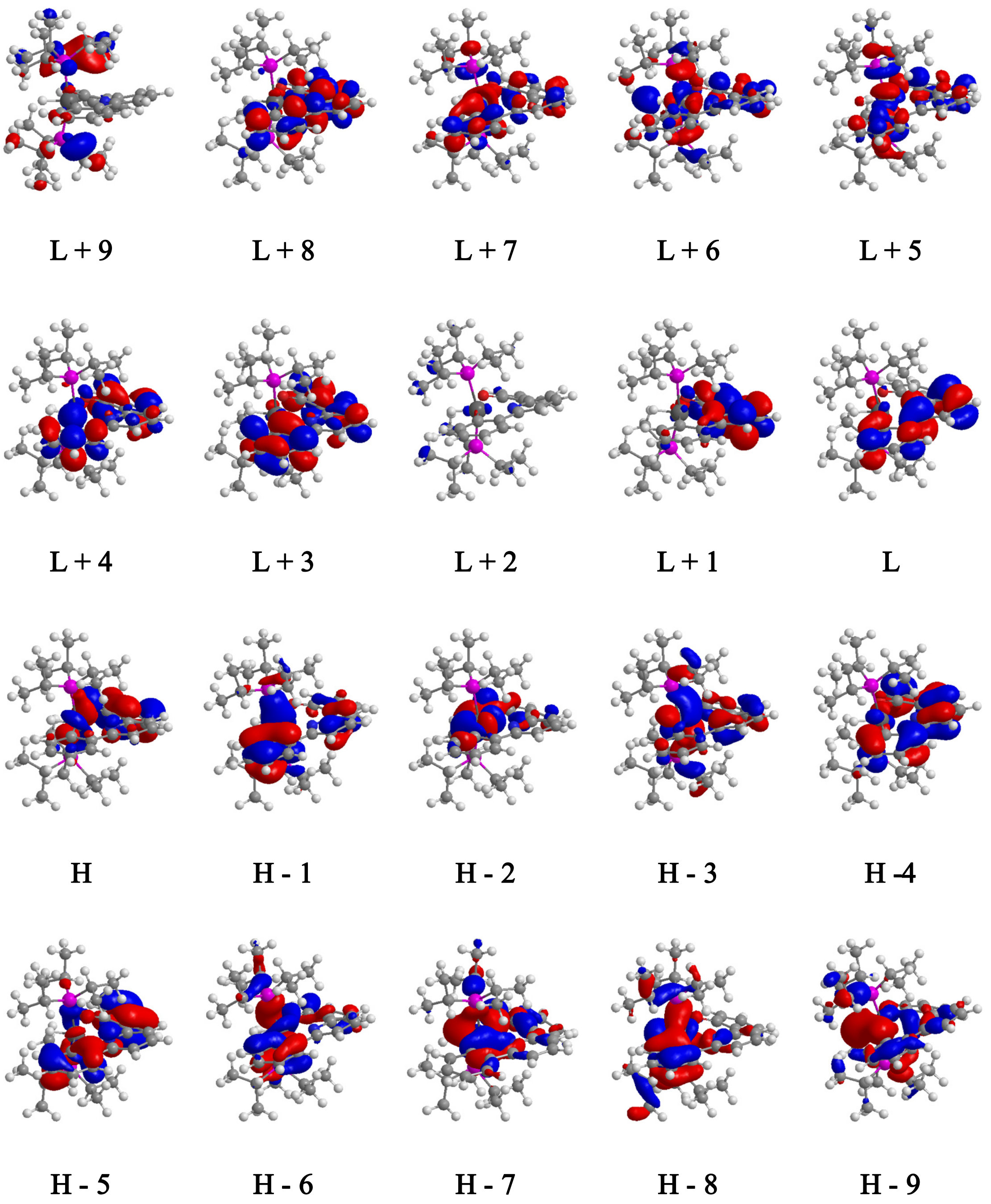

Figure S52. Molecular orbitals of complex **5** (isovalue 0.003 au).

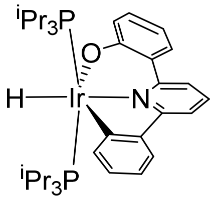

5

Table S11. Composition (%) of the molecular orbitals of complex 6.

MO	eV	Iridium	P^{i}Pr$_3$ (1)	P^{i}Pr$_3$ (2)	H	L (Ph)	L (Im)	L (PhO)
L+9	1.64	68	4	4	0	3	9	11
L+8	1.57	85	1	0	0	1	10	2
L+7	1.55	23	1	1	0	12	47	15
L+6	1.27	89	5	4	0	0	1	1
L+5	0.82	3	1	1	0	3	5	87
L+4	0.67	39	21	21	1	8	0	9
L+3	0.47	6	1	1	0	88	1	2
L+2	0.20	90	4	5	0	1	0	0
L+1	-0.05	0	1	0	0	25	35	38
LUMO	-0.21	5	1	1	0	37	28	28
HOMO	-4.56	17	4	4	0	2	9	64
H-1	-5.23	61	2	1	1	9	3	24
H-2	-5.23	32	6	6	0	41	7	7
H-3	-5.47	23	5	5	0	18	31	19
H-4	-6.09	5	2	1	0	32	10	50
H-5	-6.41	15	13	14	3	16	13	26
H-6	-6.43	20	8	7	33	2	25	3
H-7	-6.79	18	10	10	0	32	9	20
H-8	-6.99	30	21	21	0	24	3	1
H-9	-7.10	29	2	3	1	17	2	44

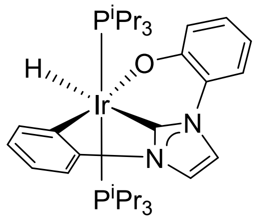

6

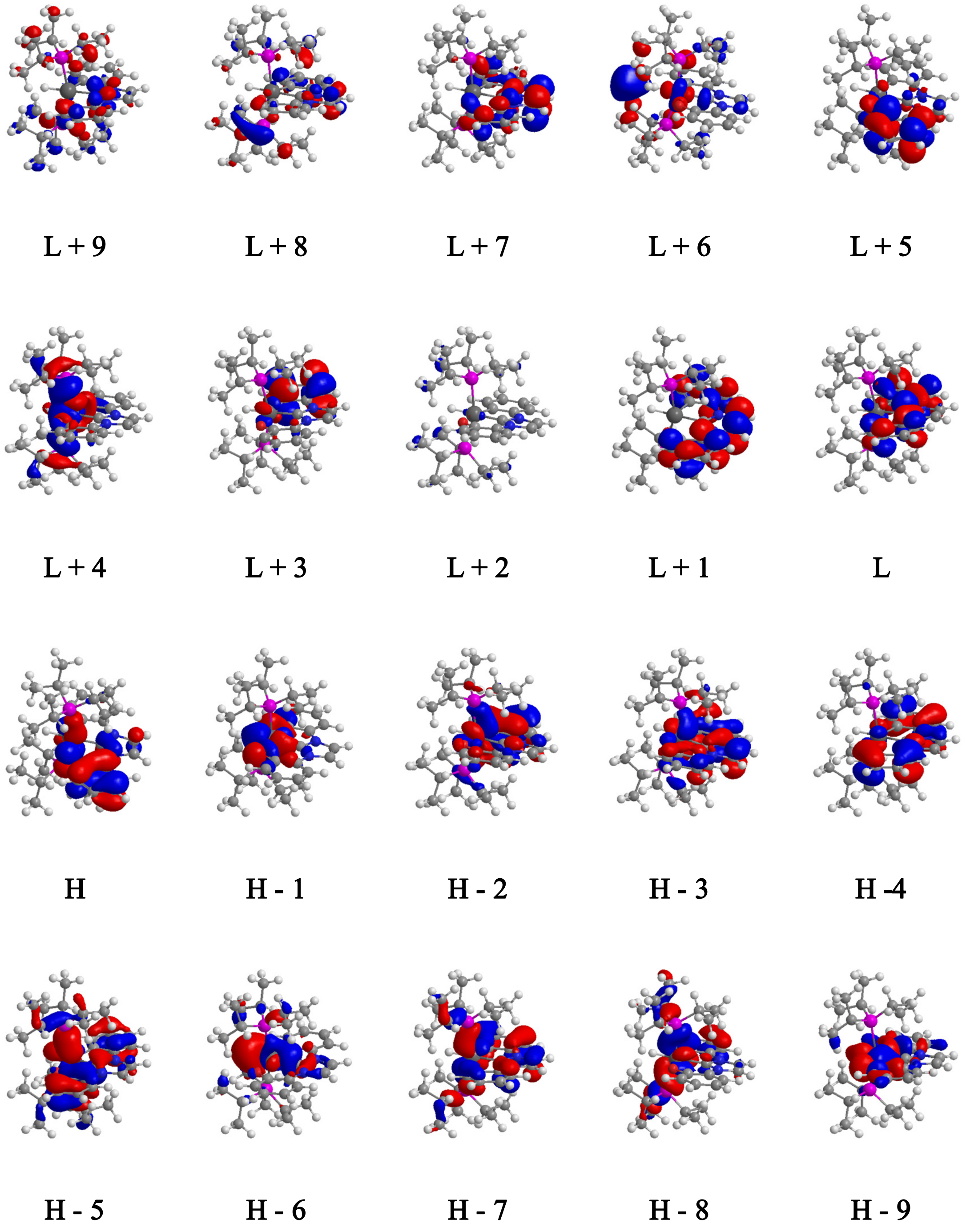

Figure S53. Molecular orbitals of complex **6** (isovalue 0.003 au).

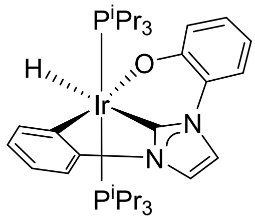

6

Table S12. Composition (%) of the molecular orbitals of complex 7

MO	eV	Iridium	P^{i}Pr$_3$ (1)	P^{i}Pr$_3$ (2)	H	L (Py-C)	L (Ph)	L (Py-N)
L+9	1.64	13	2	6	0	28	37	14
L+8	1.45	105	0	-1	0	0	2	-5
L+7	1.35	93	1	4	0	0	1	1
L+6	1.14	6	1	1	0	5	75	12
L+5	0.86	38	23	24	2	8	0	5
L+4	0.44	5	2	1	0	84	6	1
L+3	0.18	90	4	5	0	1	0	0
L+2	-0.19	3	1	1	0	45	20	30
L+1	-0.65	2	1	1	0	11	15	69
LUMO	-1.14	3	1	1	0	5	28	62
HOMO	-4.82	26	4	4	0	20	42	4
H-1	-5.01	31	6	6	0	26	25	6
H-2	-5.19	79	1	2	0	9	3	6
H-3	-5.52	15	5	4	31	3	41	1
H-4	-5.84	18	4	4	0	14	45	16
H-5	-6	7	1	0	1	83	5	3
H-6	-6.11	26	13	13	0	14	19	14
H-7	-6.8	32	24	27	0	11	3	3
H-8	-7.09	6	3	4	0	46	35	6
H-9	-7.23	15	12	12	0	51	8	2

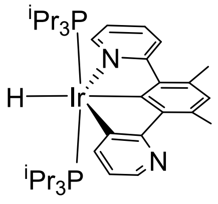

7

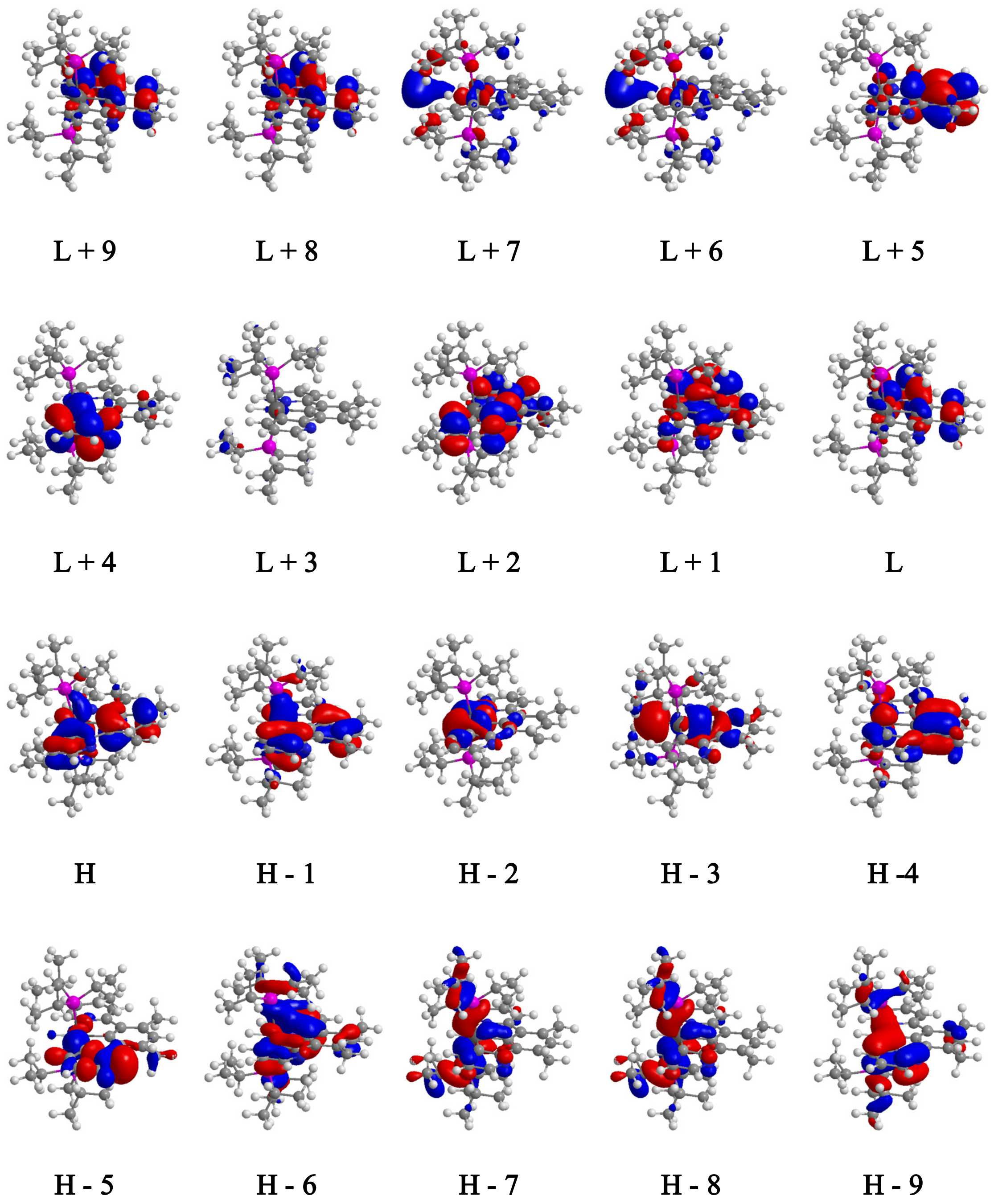

Figure S54. Molecular orbitals of **complex 7** (isovalue 0.003 au).

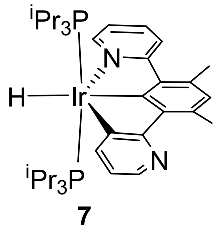

7

Cyclic Voltammograms

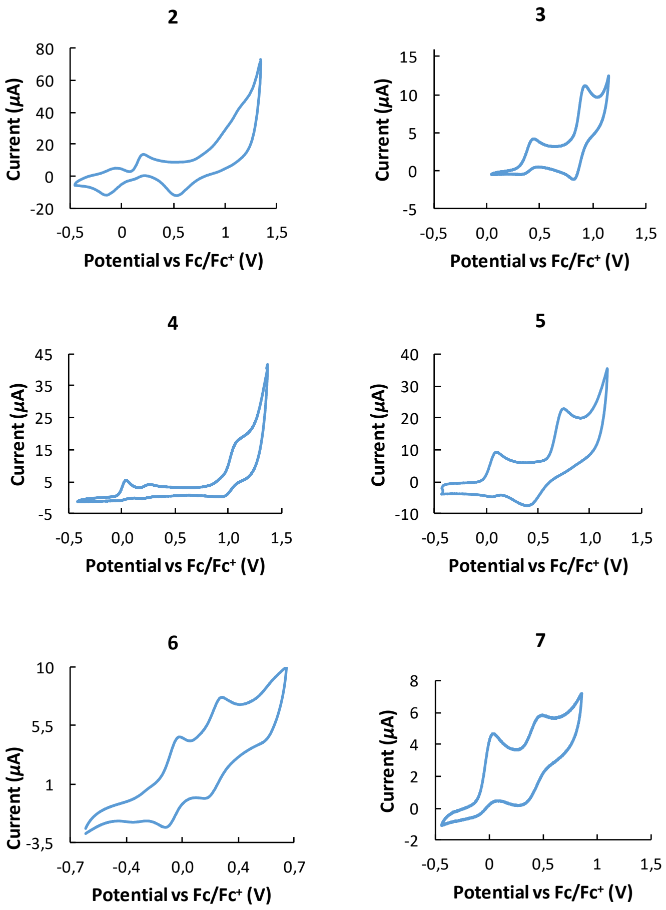

Figure S55. Cyclic voltammograms of complexes **2**–**7** in CH_2Cl_2 (10^{-3} M) with Bu_4NPF_6 as supporting electrolyte (0.1 M). The potentials are referenced to the ferrocenium/ferrocene (Fc^+/Fc) couple.

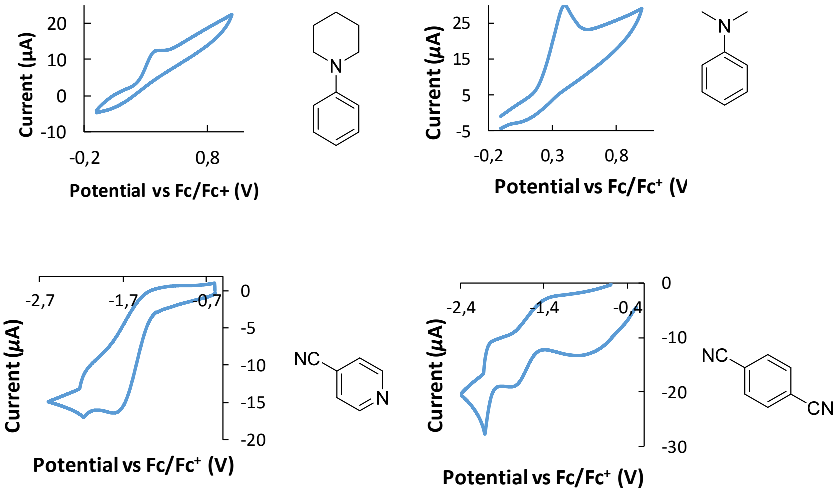

Figure S56. Cyclic voltammograms of organic reacts in CH_2Cl_2 (10^{-3} M) with Bu_4NPF_6 as supporting electrolyte (0.1 M). The potentials are referenced to the ferrocene/ferrocenium (Fc/Fc^+) couple.

Normalized Emission and Excitation Spectra of 2-5 and 7

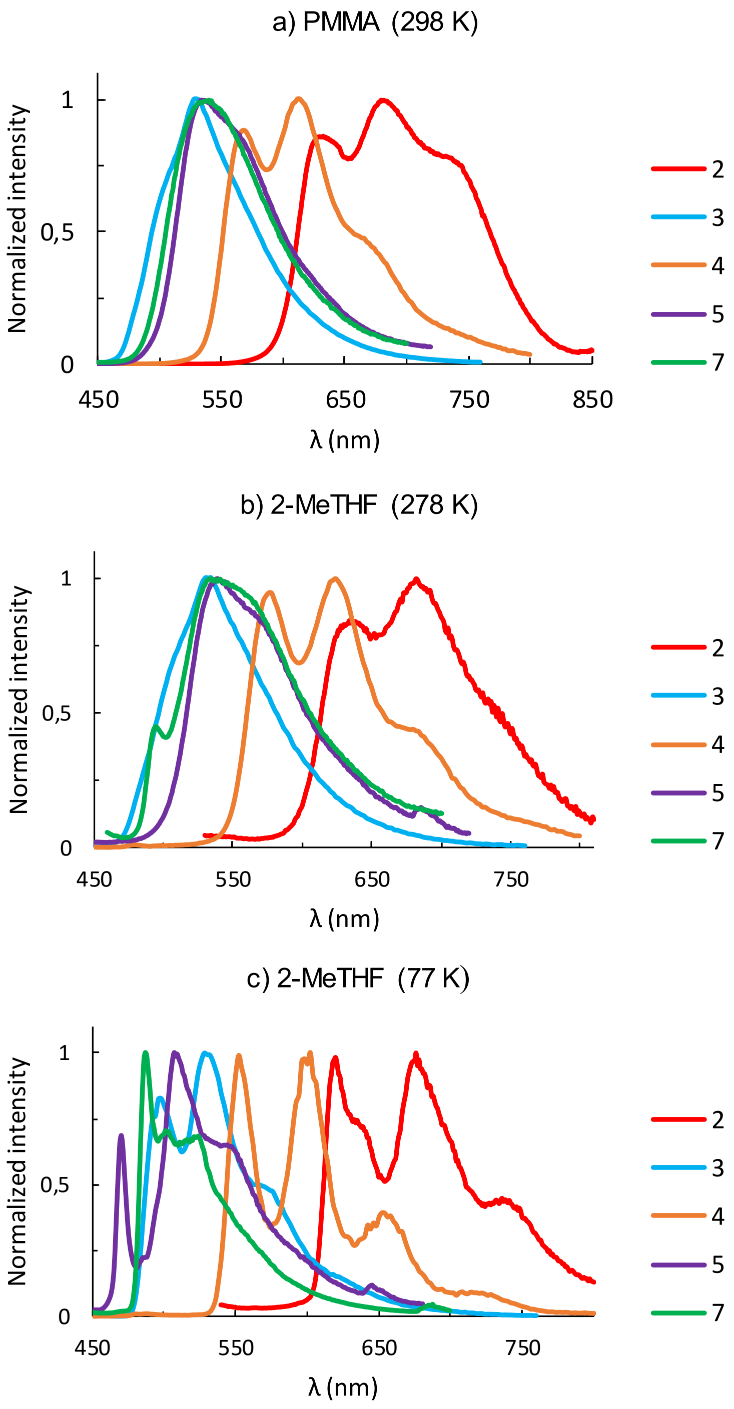

Figure S57. Normalized emission spectra of **2-5** and **7** in (a) 5 wt% PMMA films at 298 K, (b) 2-MeTHF at 298 K, and (c) 2-MeTHF at 77 K.

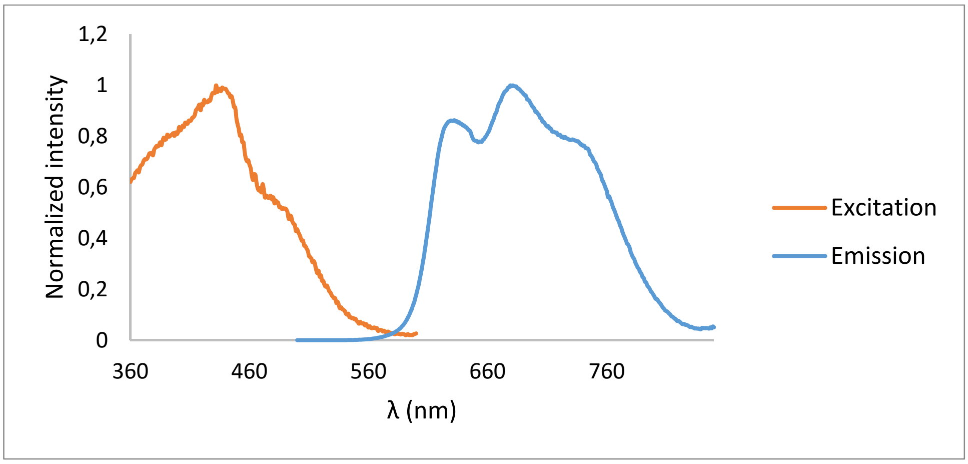

Figure S58. Normalized emission (blue line; λ_{exc} = 450 nm) and excitation (orange line; λ_{em} = 640 nm) spectrum of complex **2** in PMMA film (5 wt %) at 298 K.

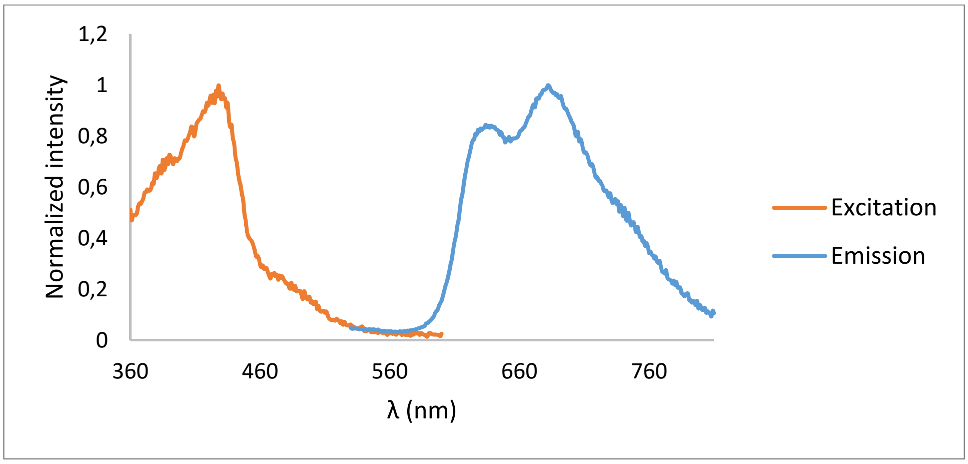

Figure S59. Normalized emission (blue line; λ_{exc} = 450 nm) and excitation (orange line; λ_{em} = 640 nm) spectrum for a 1 x 10^{-5} M solution of complex **2** in 2-MeTHF at 298 K.

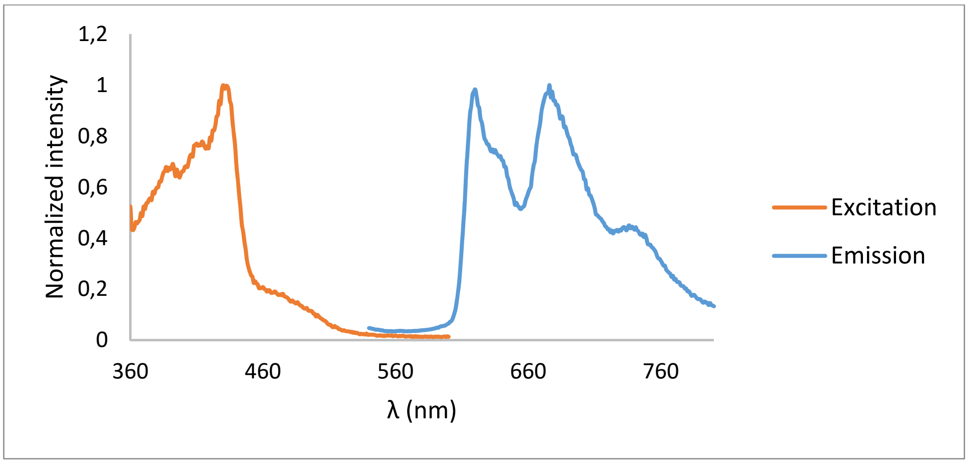

Figure S60. Normalized emission (blue line; λ_{exc} = 450 nm) and excitation (orange line; λ_{em} = 640 nm) spectrum for a 1 x 10^{-5} M solution of complex **2** in 2-MeTHF at 77 K.

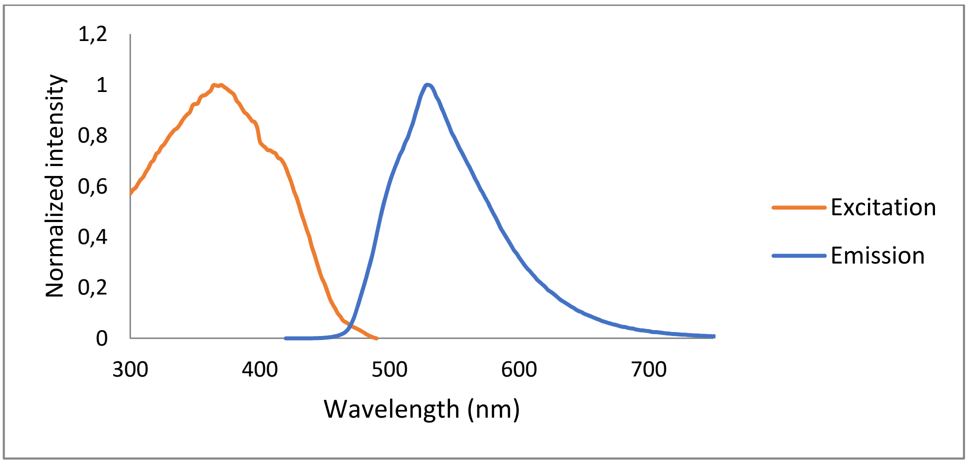

Figure S61. Normalized emission (blue line; λ_{exc} = 400 nm) and excitation (orange line; λ_{em} = 530 nm) spectrum of complex **3** in PMMA film (5 wt %) at 298 K.

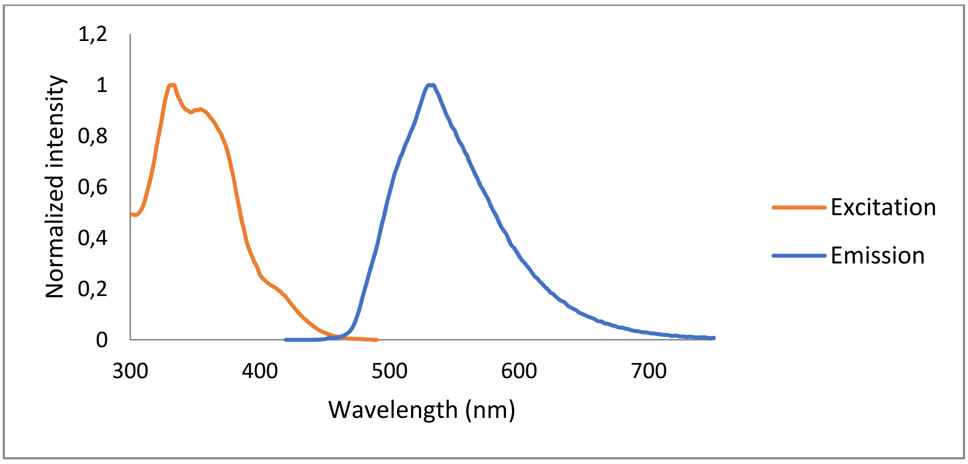

Figure S62. Normalized emission (blue line; λ_{exc} = 400 nm) and excitation (orange line; λ_{em} = 530 nm) spectrum for a 1 x 10^{-5} M solution of complex **3** in 2-MeTHF at 298 K.

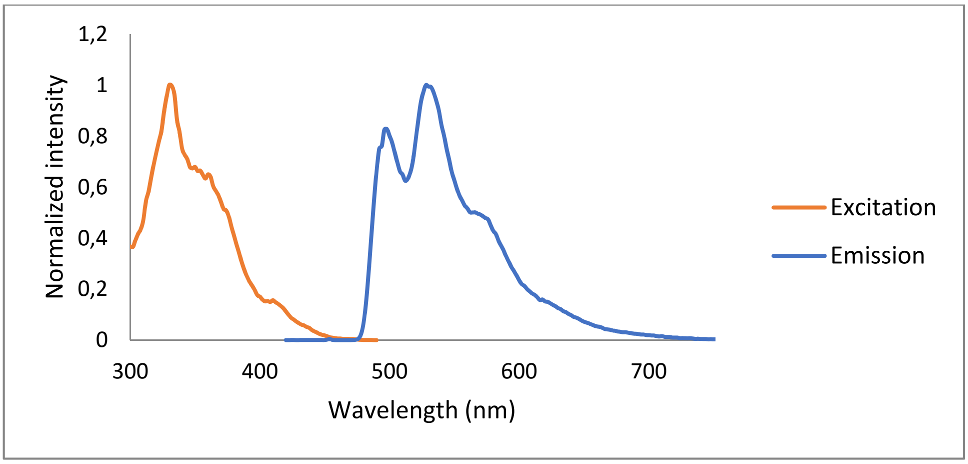

Figure S63. Normalized emission (blue line; λ_{exc} = 400 nm) and excitation (orange line; λ_{em} = 530 nm) spectrum for a 1 x 10^{-5} M solution of complex **3** in 2-MeTHF at 77 K.

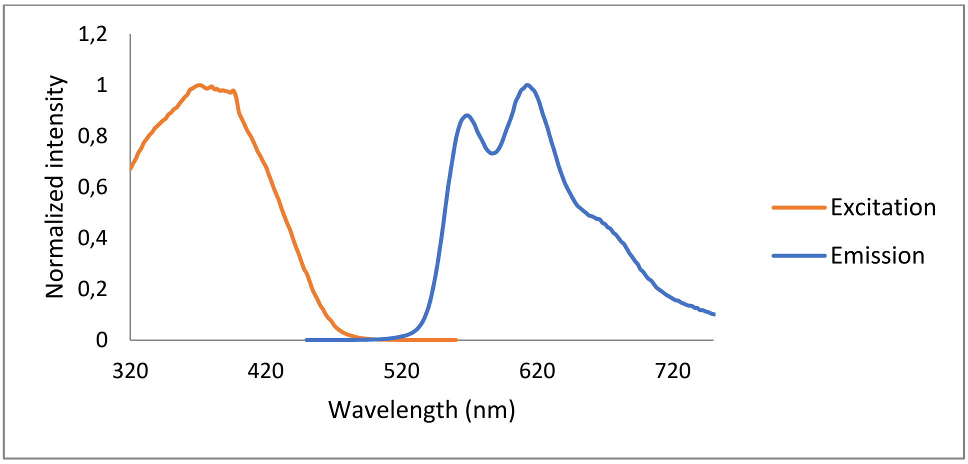

Figure S64. Normalized emission (blue line; λ_{exc} = 420 nm) and excitation (orange line; λ_{em} = 600 nm) spectrum of complex **4** in PMMA film (5 wt %) at 298 K.

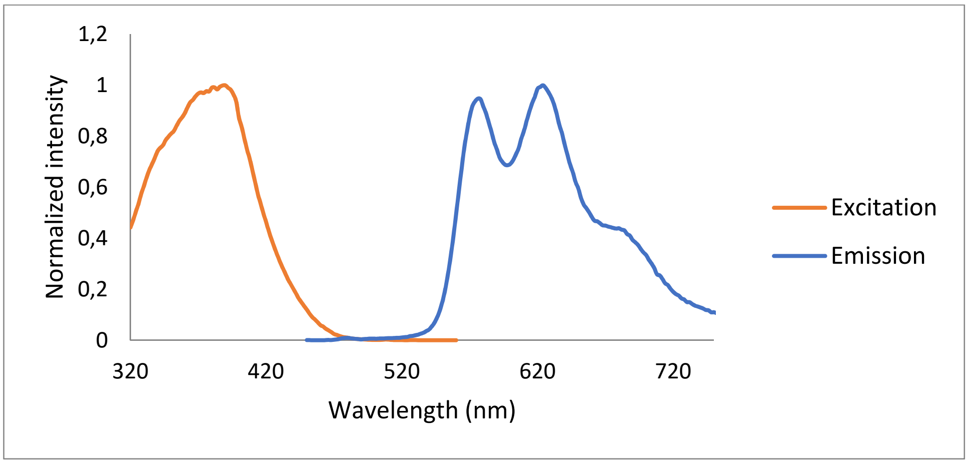

Figure S65. Normalized emission (blue line; λ_{exc} = 420 nm) and excitation (orange line; λ_{em} = 600 nm) spectrum for a 1 x 10^{-5} M solution of complex **4** in 2-MeTHF at 298 K.

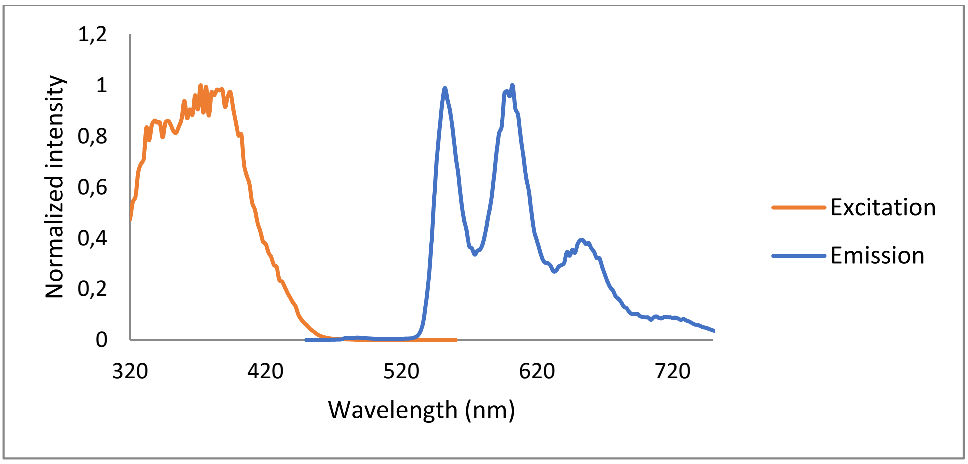

Figure S66. Normalized emission (blue line; λ_{exc} = 420 nm) and excitation (orange line; λ_{em} = 600 nm) spectrum for a 1 x 10^{-5} M solution of complex **4** in 2-MeTHF at 77 K.

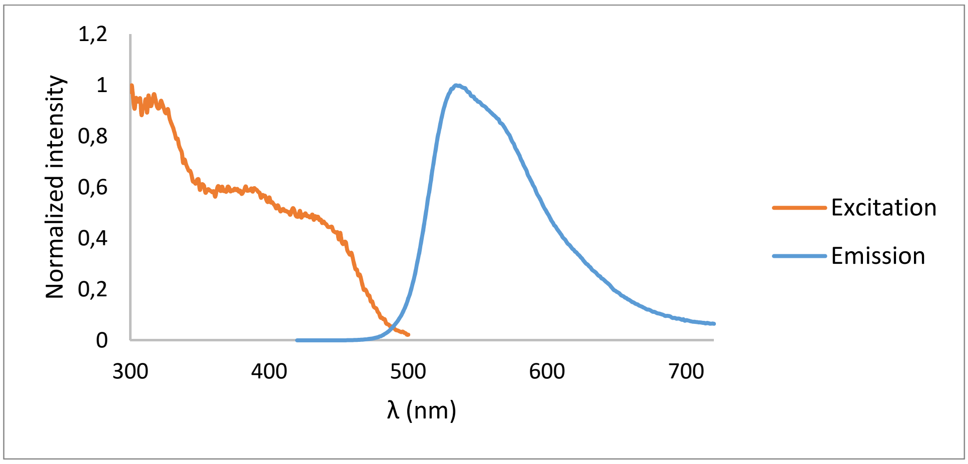

Figure S67. Normalized emission (blue line; λ_{exc} = 380 nm) and excitation (orange line; λ_{em} = 540 nm) spectrum of complex **5** in PMMA film (5 wt %) at 298 K.

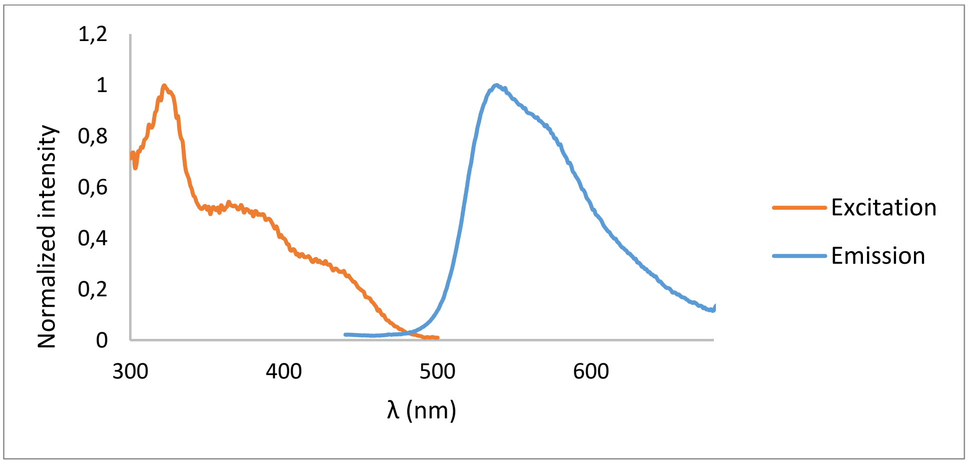

Figure S68. Normalized emission (blue line; λ_{exc} = 380 nm) and excitation (orange line; λ_{em} = 540 nm) spectrum for a 1 x 10^{-5} M solution of complex **5** in 2-MeTHF at 298 K.

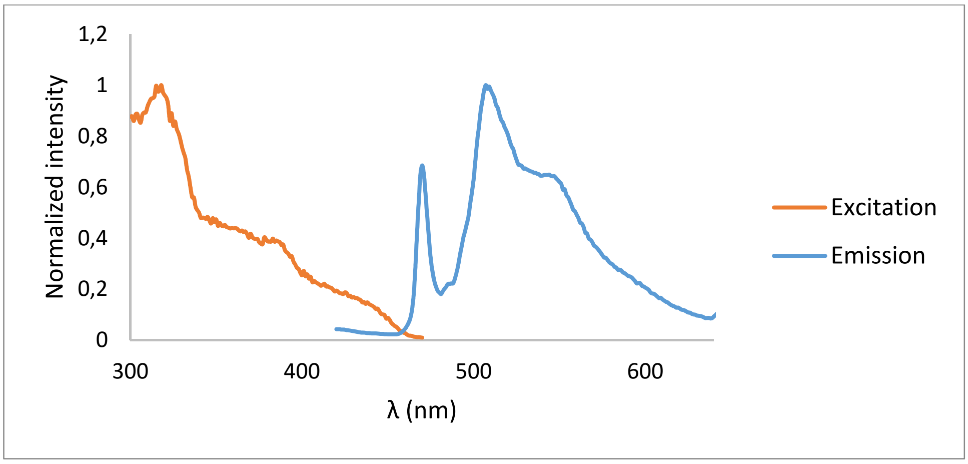

Figure S69. Normalized emission (blue line; λ_{exc} = 360 nm) and excitation (orange line; λ_{em} = 510 nm) spectrum for a 1 x 10^{-5} M solution of complex **5** in 2-MeTHF at 77 K.

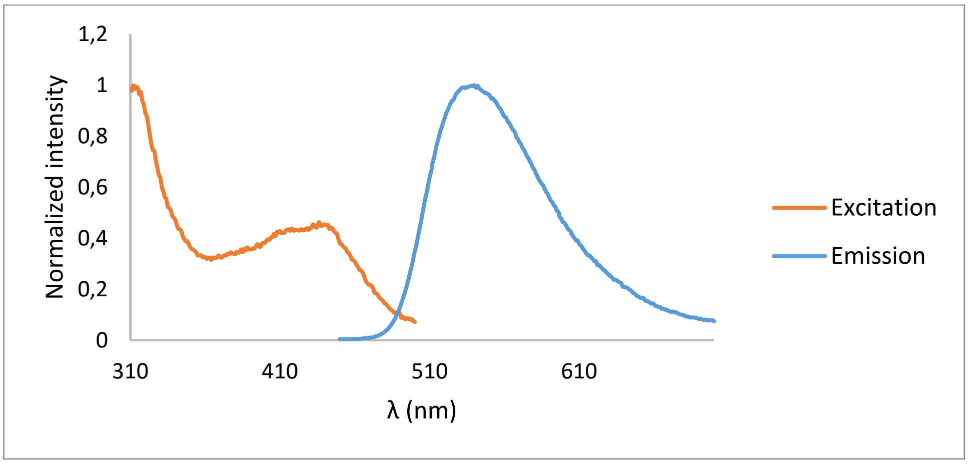

Figure S70. Normalized emission (blue line; λ_{exc} = 400 nm) and excitation (orange line; λ_{em} = 540 nm) spectrum of complex **7** in PMMA film (5 wt %) at 298 K.

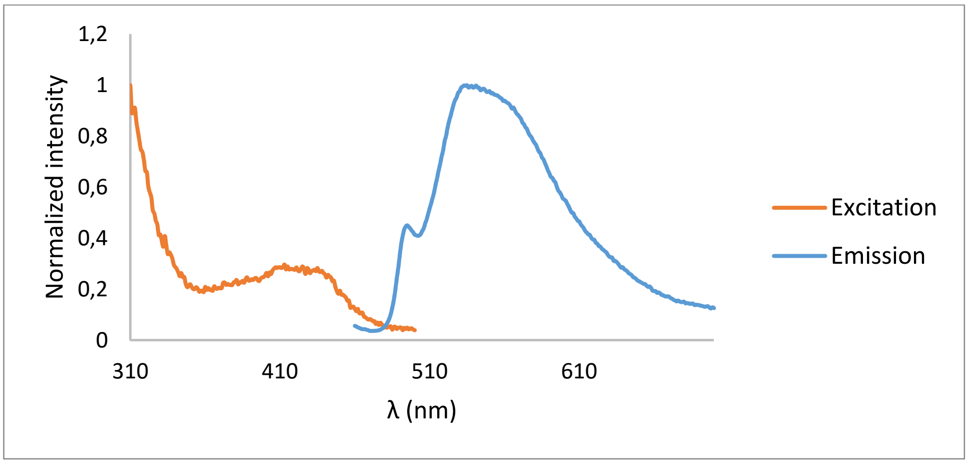

Figure S71. Normalized emission (blue line; λ_{exc} = 400 nm) and excitation (orange line; λ_{em} = 540 nm) spectrum for a 1 x 10^{-5} M solution of complex **7** in 2-MeTHF at 298 K.

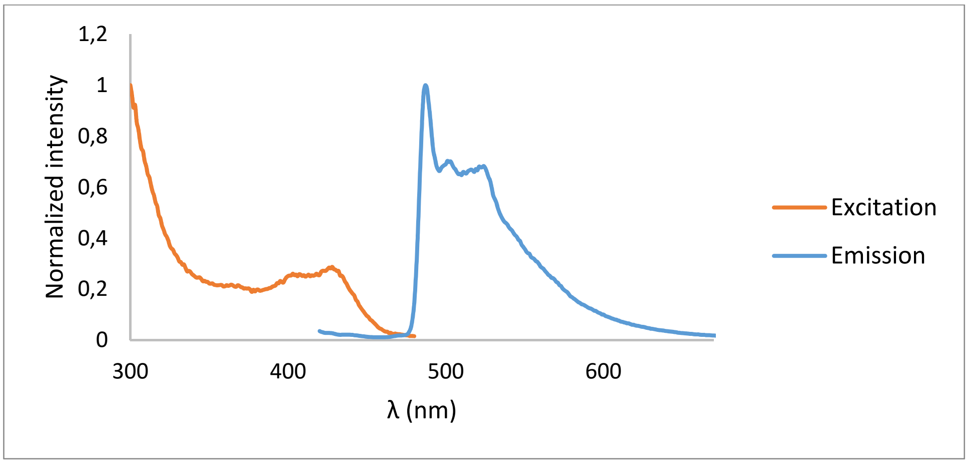

Figure S72. Normalized emission (blue line; λ_{exc} = 380 nm) and excitation (orange line; λ_{em} = 520 nm) spectrum for a 1 x 10^{-5} M solution of complex **7** in 2-MeTHF at 77 K.

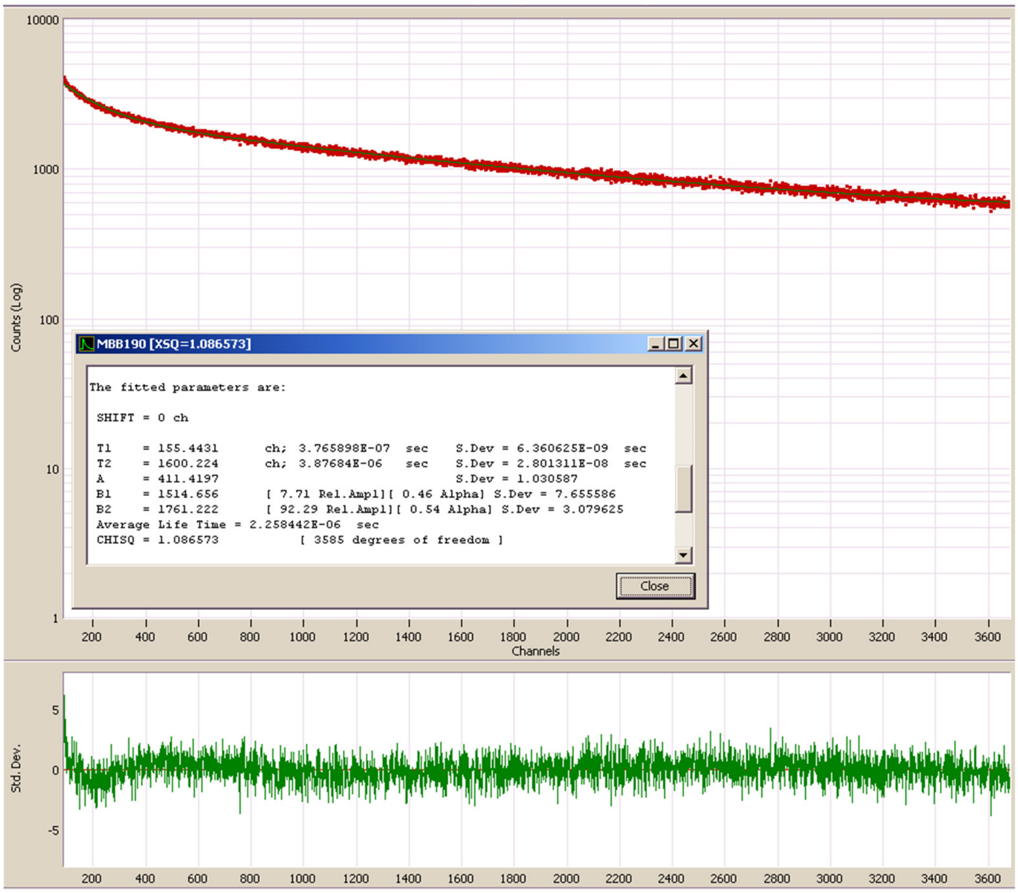

Figure S73. Raw (experimental) time-resolved photoluminescence decay of **2** in PMMA film (5 wt%) at 298 K (λ_{exc} = 390 nm, λ_{em} = 640 nm), fitting parameters, and confidence limits.

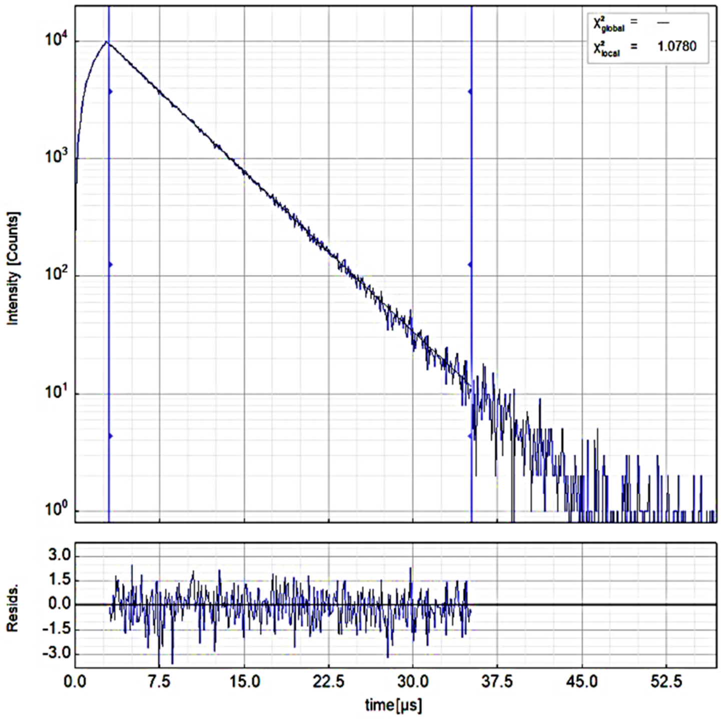

Parameter	Value	Δ
A_1[kCnts/Chnl]	9.329	±0.022
τ_1[ns]	4 776.5	±8.9
I_1[kCnts]	556.99	±0.44
$Bkgr_{Dec}$[kCnts]	0.0003	±0.0012
τ_{AvInt}[ns]	4 776.5	±8.9

Figure S74. Left: Raw (experimental) time-resolved photoluminescence decay of **2** in 2-MeTHF at 298 K (λ_{exc} = 452 nm, λ_{em} = 627 nm). Right: Fitting parameters and confidence limits.

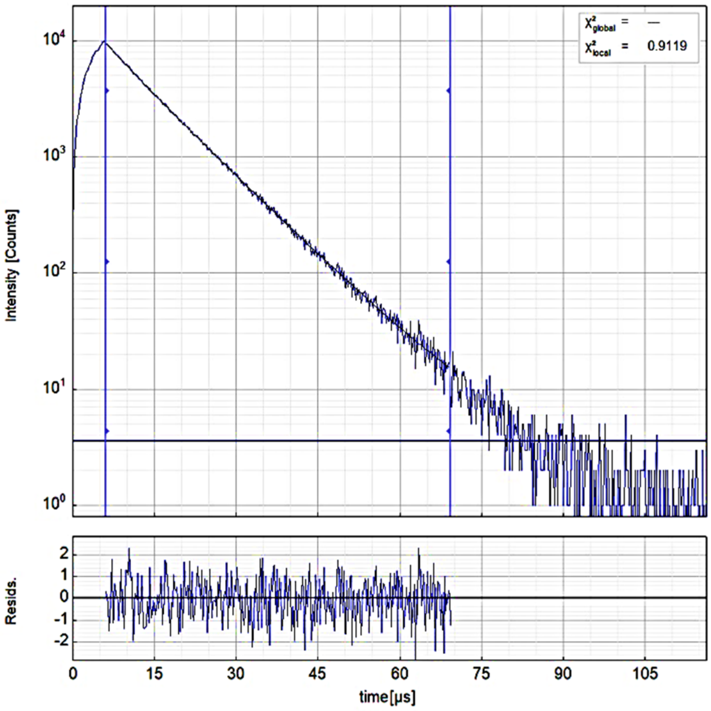

Parameter	Value	Δ
A_1[kCnts/Chnl]	7.84	±0.57
τ_1[ns]	9 710	±160
I_1[kCnts]	476	±27
A_2[kCnts/Chnl]	1.71	±0.59
τ_2[ns]	5 320	±640
I_2[kCnts]	57	±28
$Bkgr_{Dec}$[kCnts]	0.0036	±0.0015
τ_{AvInt}[ns]	9 238	±45

Figure S75. Left: Raw (experimental) time-resolved photoluminescence decay of **2** in 2-MeTHF at 77 K (λ_{exc} = 452 nm, λ_{em} = 617 nm). Right: Fitting parameters and confidence limits.

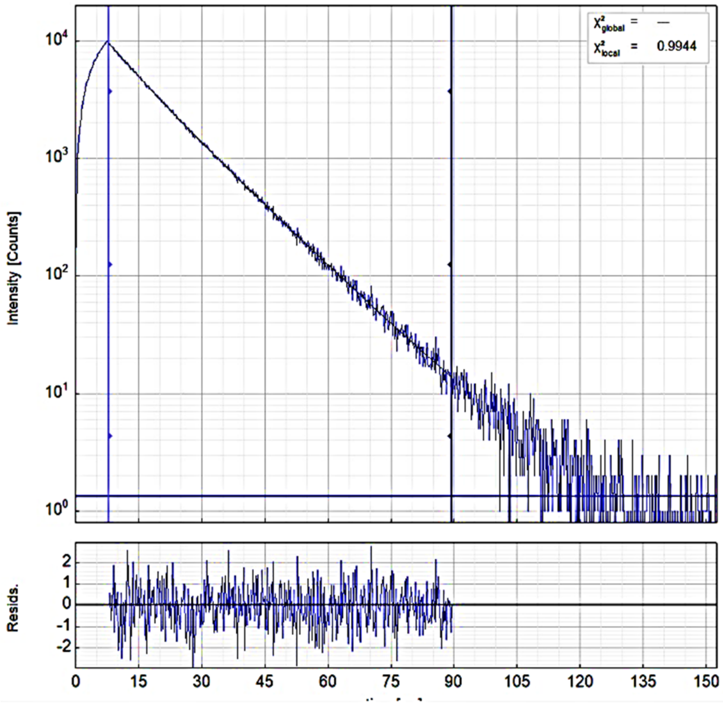

Parameter	Value	Δ
A_1[kCnts/Chnl]	6.67	±0.68
τ_1[ns]	12 970	±310
I_1[kCnts]	541	±41
A_2[kCnts/Chnl]	2.81	±0.66
τ_2[ns]	7 500	±790
I_2[kCnts]	132	±42
$Bkgr_{Dec}$[kCnts]	0.0014	±0.0015
τ_{AvInt}[ns]	11 893	±33

Figure S76. Left: Raw (experimental) time-resolved photoluminescence decay of **3** in PMMA film (5 wt%) at 298 K (λ_{exc} = 378 nm, λ_{em} = 529 nm). Right: Fitting parameters and confidence limits.

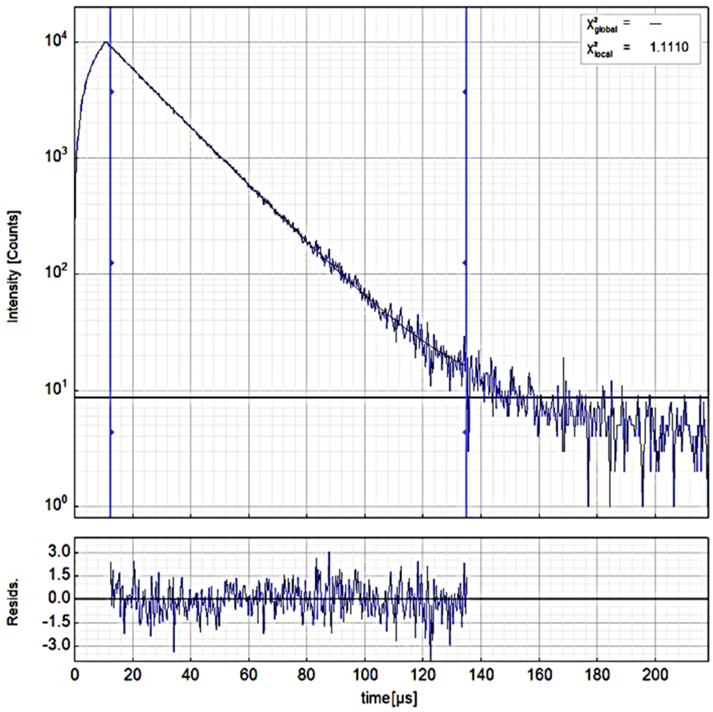

Parameter	Value	Δ
A_1[kCnts/Chnl]	9.055	±0.014
τ_1[ns]	17 282	±19
I_1[kCnts]	488.98	±0.77
$Bkgr_{Dec}$[kCnts]	0.0086	±0.0008
τ_{AvInt}[ns]	17 282	±19

Figure S77. Left: Raw (experimental) time-resolved photoluminescence decay of **3** in 2-MeTHF at 298 K (λ_{exc} = 378 nm, λ_{em} = 534 nm). Right: Fitting parameters and confidence limits.

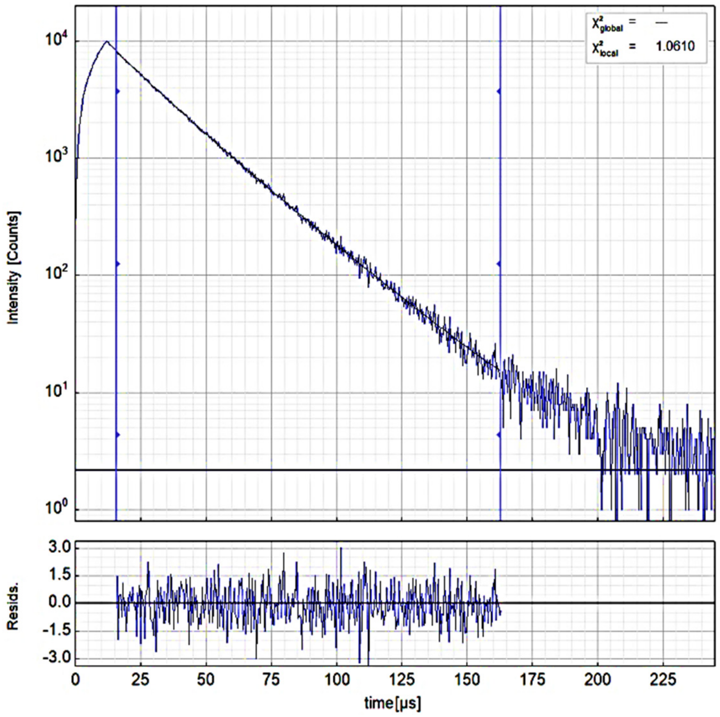

Parameter	Value	Δ
A_1[kCnts/Chnl]	5.4	±2.3
τ_1[ns]	24 400	±3 000
I_1[kCnts]	410	±140
A_2[kCnts/Chnl]	2.8	±2.3
τ_2[ns]	15 400	±2 800
I_2[kCnts]	140	±150
$Bkgr_{Dec}$[kCnts]	0.0022	±0.0032
τ_{AvInt}[ns]	22 170	±240

Figure S78. Left: Raw (experimental) time-resolved photoluminescence decay of **3** in 2-MeTHF at 77 K (λ_{exc} = 378 nm, λ_{em} = 529 nm). Right: Fitting parameters and confidence limits.

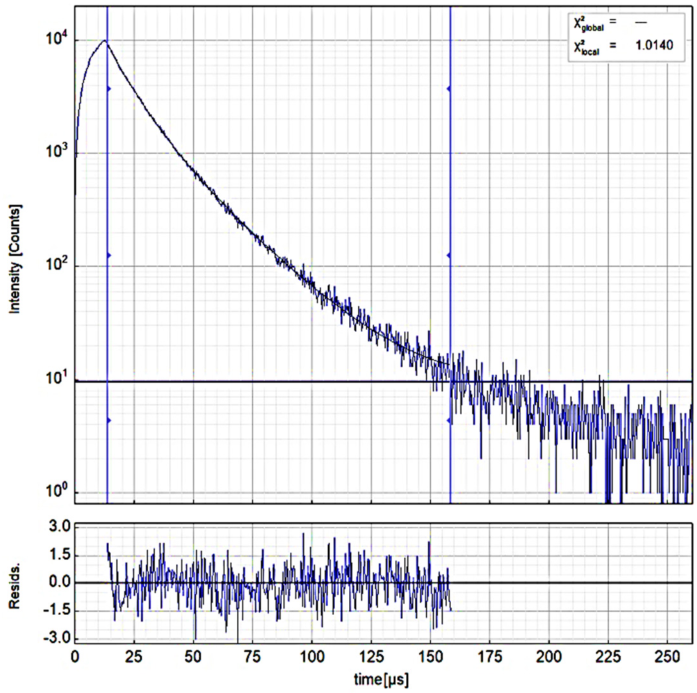

Parameter	Value	Δ
A_1[kCnts/Chnl]	2.95	±0.16
τ_1[ns]	21 900	±500
I_1[kCnts]	201.9	±7.6
A_2[kCnts/Chnl]	6.18	±0.18
τ_2[ns]	9 270	±280
I_2[kCnts]	179.0	±7.2
$Bkgr_{Dec}$[kCnts]	0.0095	±0.0011
τ_{AvInt}[ns]	15 970	±110

Figure S79. Left: Raw (experimental) time-resolved photoluminescence decay of **4** in PMMA film (5 wt%) at 298 K (λ_{exc} = 405 nm, λ_{em} = 565 nm). Right: Fitting parameters and confidence limits.

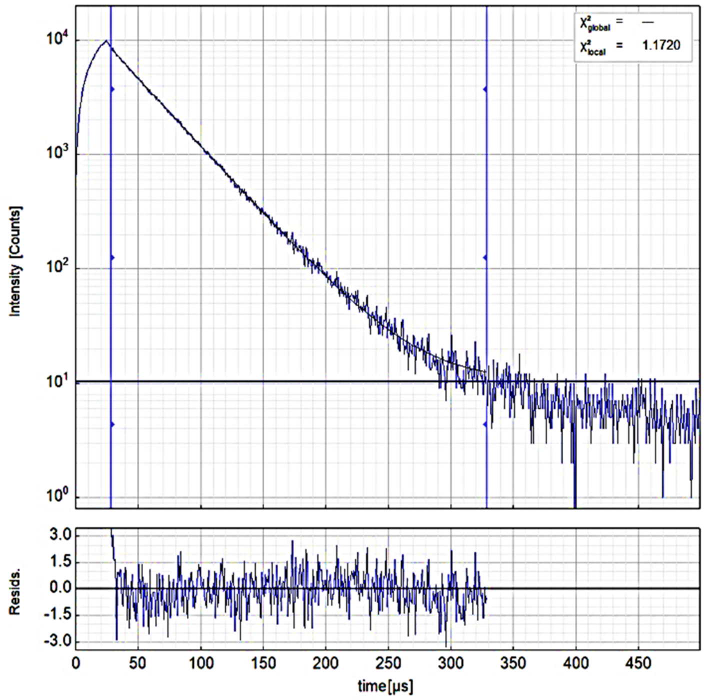

Parameter	Value	Δ
A_1[kCnts/Chnl]	8.4313	±0.0089
τ_1[ns]	36 477	±43
I_1[kCnts]	480.54	±0.55
$Bkgr_{Dec}$[kCnts]	0.0103	±0.0005
τ_{AvInt}[ns]	36 477	±43

Figure S80. Left: Raw (experimental) time-resolved photoluminescence decay of **4** in 2-MeTHF at 298 K (λ_{exc} = 405 nm, λ_{em} = 575 nm). Right: Fitting parameters and confidence limits.

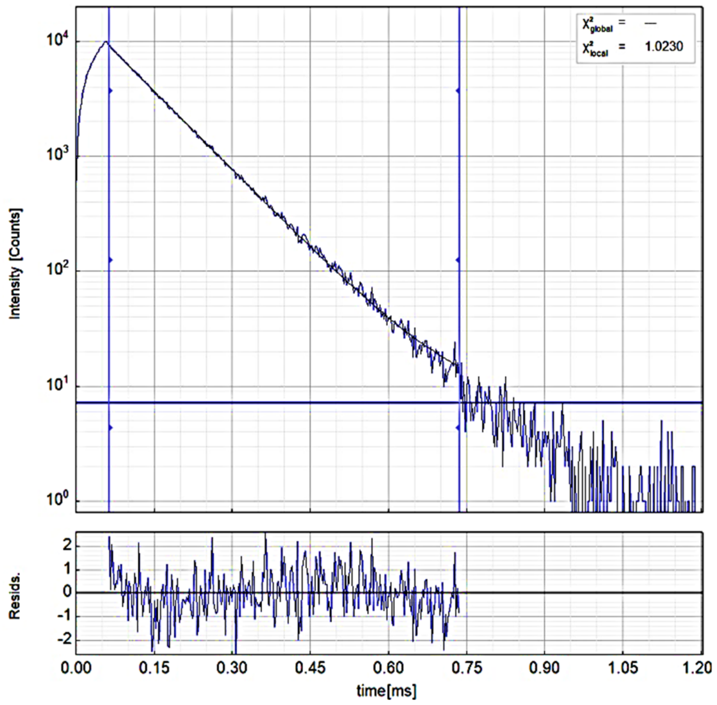

Parameter	Value	Δ
A_1[kCnts/Chnl]	9.089	±0.016
τ_1[ns]	95 030	±200
I_1[kCnts]	337.36	±0.47
$Bkgr_{Dec}$[kCnts]	0.0072	±0.0006
τ_{AvInt}[ns]	95 030	±200

Figure S81. Left: Raw (experimental) time-resolved photoluminescence decay of **4** in 2-MeTHF at 77 K (λ_{exc} = 405 nm, λ_{em} = 555 nm). Right: Fitting parameters and confidence limits.

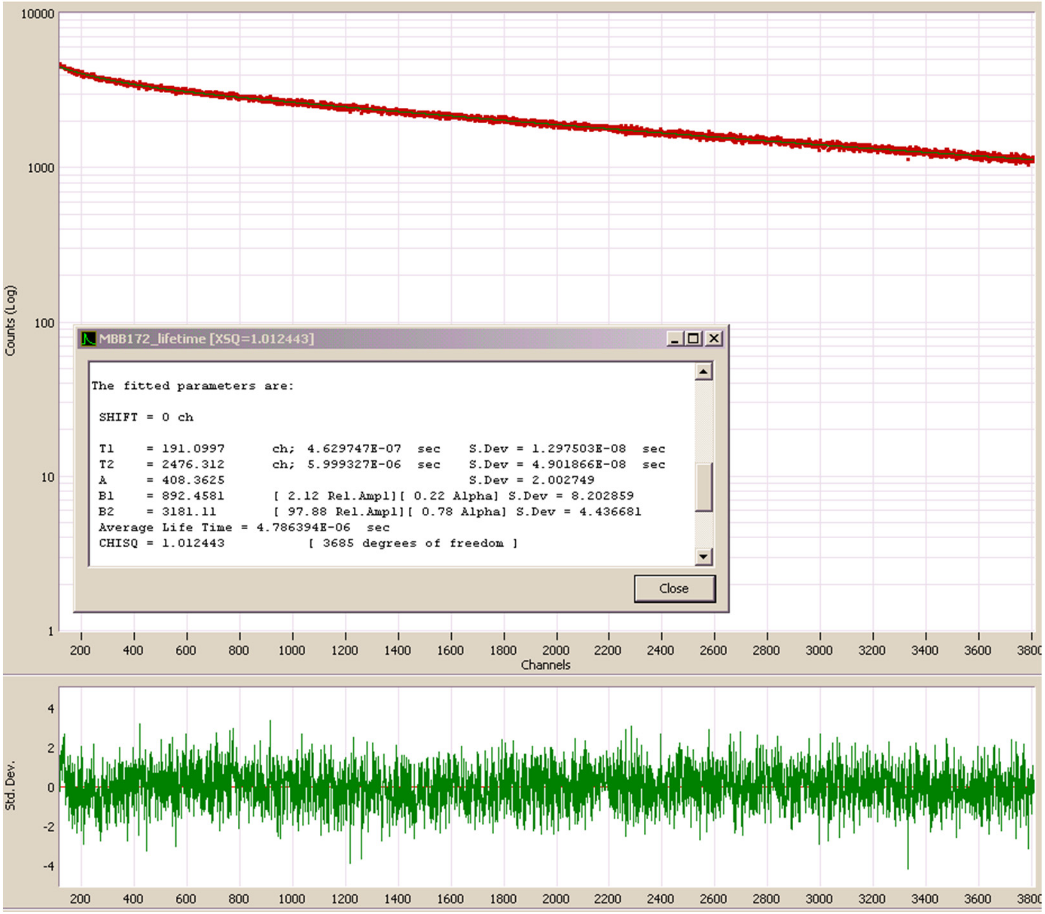

Figure S82. Raw (experimental) time-resolved photoluminescence decay of **5** in PMMA film (5 wt%) at 298 K (λ_{exc} = 390 nm, λ_{em} = 540 nm), fitting parameters and confidence limits.

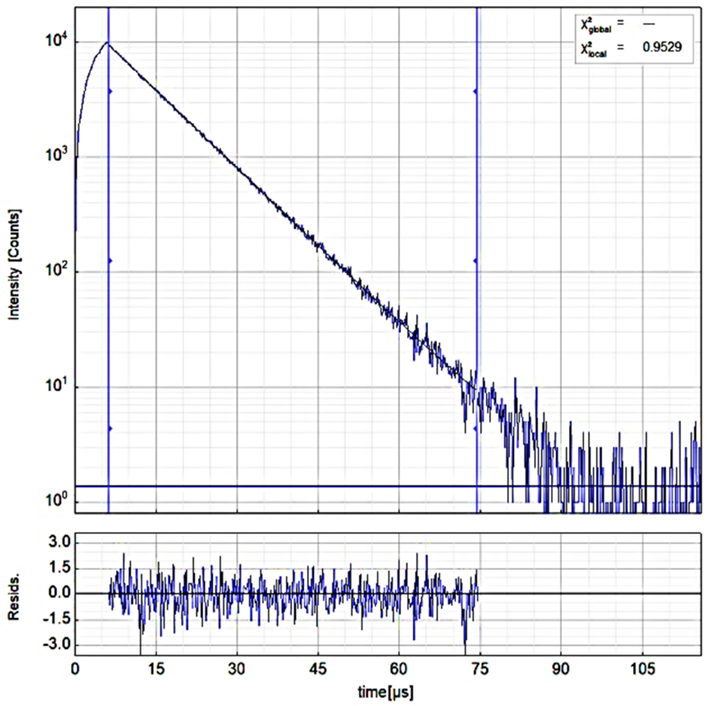

Parameter	Value	Δ
A_1[kCnts/Chnl]	9.450	±0.025
τ_1[ns]	9 626	±15
I_1[kCnts]	568.48	±0.85
$Bkgr_{Dec}$[kCnts]	0.0014	±0.0003
τ_{AvInt}[ns]	9 626	±15

Figure S83. Left: Raw (experimental) time-resolved photoluminescence decay of **5** in 2-MeTHF at 298 K (λ_{exc} = 378 nm, λ_{em} = 534 nm). Right: Fitting parameters and confidence limits.

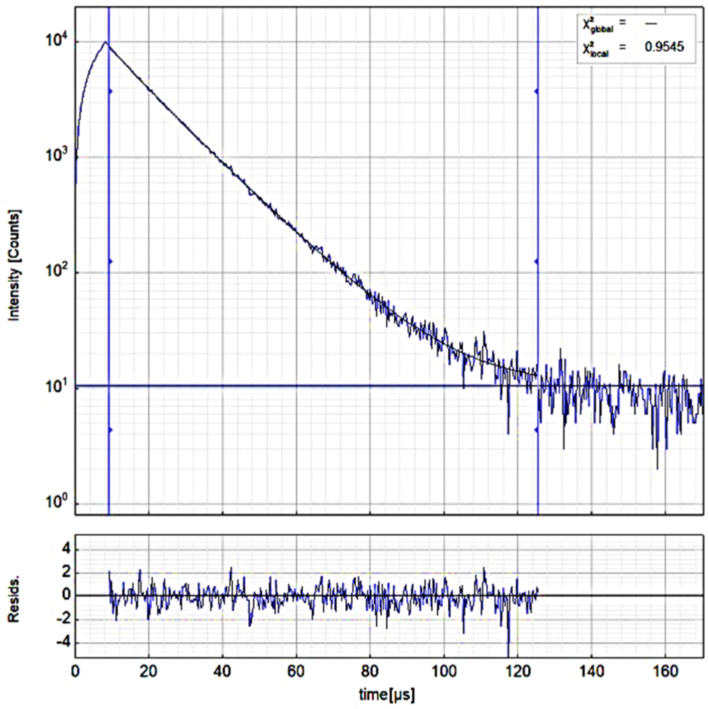

Parameter	Value	Δ
A_1[kCnts/Chnl]	6.3	±1.7
τ_1[ns]	14 800	±740
I_1[kCnts]	290	±59
A_2[kCnts/Chnl]	2.8	±1.7
τ_2[ns]	9 400	±2 000
I_2[kCnts]	81	±59
$Bkgr_{Dec}$[kCnts]	0.0105	±0.0012
τ_{AvInt}[ns]	13 615	±63

Figure S84. Left: Raw (experimental) time-resolved photoluminescence decay of **5** in 2-MeTHF at 77 K (λ_{exc} = 378 nm, λ_{em} = 514 nm). Right: Fitting parameters and confidence limits.

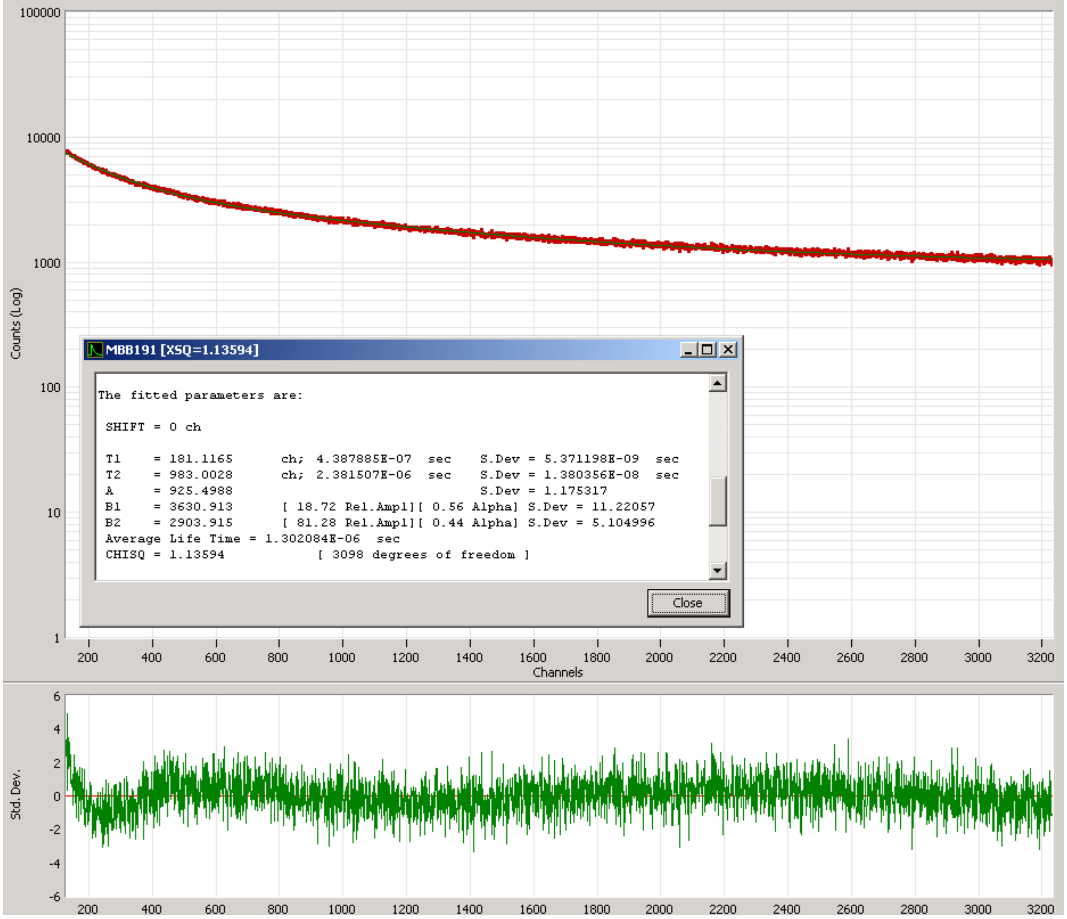

Figure S85. Raw (experimental) time-resolved photoluminescence decay of **7** in PMMA film (5 wt%) at 298 K (λ_{exc} = 390 nm, λ_{em} = 540 nm), fitting parameters and confidence limits.

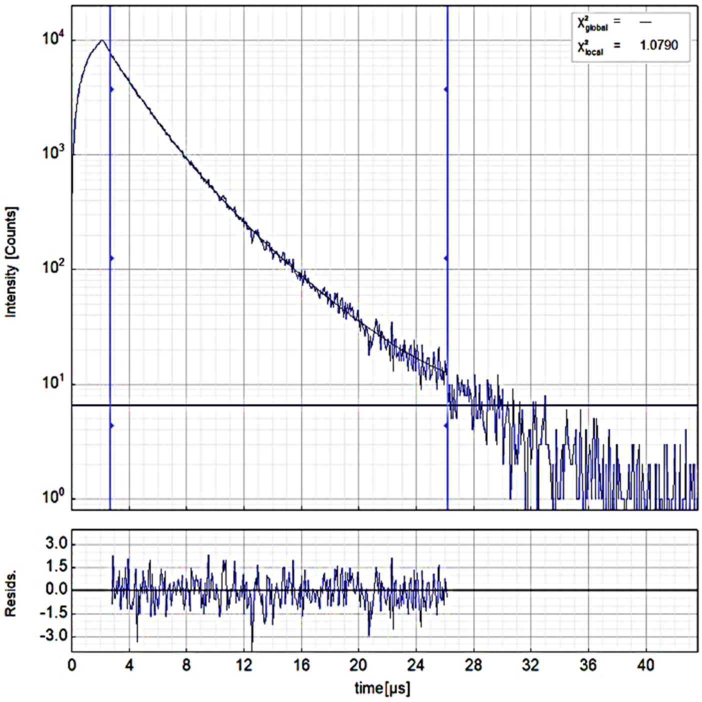

Parameter	Value	Δ
A_1[kCnts/Chnl]	2.06	±0.50
τ_1[ns]	4 010	±340
I_1[kCnts]	103	±19
A_2[kCnts/Chnl]	5.25	±0.46
τ_2[ns]	1 950	±110
I_2[kCnts]	128	±19
$Bkgr_{Dec}$[kCnts]	0.0065	±0.0016
τ_{AvInt}[ns]	2 866	±29

Figure S86. Left: Raw (experimental) time-resolved photoluminescence decay of **7** in 2-MeTHF at 298 K (λ_{exc} = 405 nm, λ_{em} = 530 nm). Right: Fitting parameters and confidence limits.

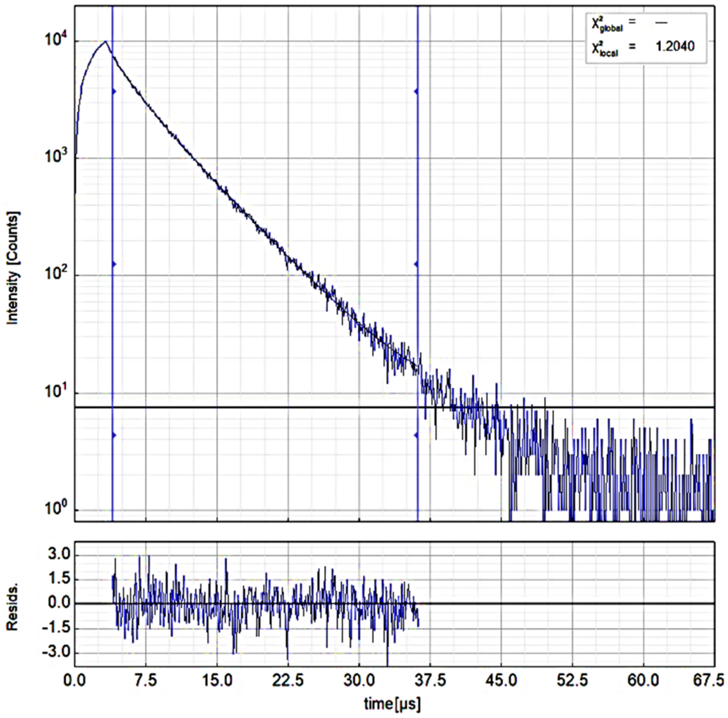

Parameter	Value	Δ
A_1[kCnts/Chnl]	5.08	±0.20
τ_1[ns]	5 128	±59
I_1[kCnts]	325.3	±7.7
A_2[kCnts/Chnl]	2.39	±0.16
τ_2[ns]	1 990	±120
I_2[kCnts]	59.2	±7.9
$Bkgr_{Dec}$[kCnts]	0.0075	±0.0010
τ_{AvInt}[ns]	4 644	±14

Figure S87. Left: Raw (experimental) time-resolved photoluminescence decay of **7** in 2-MeTHF at 77 K (λ_{exc} = 405 nm, λ_{em} = 485 nm). Right: Fitting parameters and confidence limits.

References

(1) McNally, A.; Prier, C. K.; MacMillan, D. W. C. Discovery of an *a*-Amino C–H Arylation Reaction Using the Strategy of Accelerated Serendipity. *Science* **2011**, *334*, 1114−1117.

(2) Gui, Y.Y.; Liao, L.L.; Sun, L.; Zhang, Z.; Ye, J.H.; Shen, G.; Lu, Z. P.; Zhouab, W.J.; Yu, D.G. Coupling of C(sp^3)–H bonds with C(sp^2)–O electrophiles: mild, general and selective. *Chem. Commun.* **2017**, *53*, 1192−1195.

(3) SAINT+, version 6.01: Area-Detector Integration Software, Bruker AXS, Madison,WI, 2001.

(4) Blessing, R. H. *Acta Crystallogr.* **1995**, *A51*, 33. SADABS: Area-detector absorption correction; Bruker- AXS, Madison, WI, 1996.

(5) SHELXL-2016/6. Sheldrick, G. M. *Acta Cryst.* **2008**, *A64*, 112−122.

(6) (a) Lee, C.; Yang, W.; Parr, R. G. Development of the Colle-Salvetti correlationenergy formula into a functional of the electron density. *Phys. Rev. B* **1988**, *37*, 785–789. (b) Becke, A. D. Density-functional exchange-energy approximation with correct asymptotic behavior. *J. Chem. Phys*. **1993**, *98*, 5648–5652. (c) Stephens, P. J.; Devlin, F. J.; Chabalowski, C. F.; Frisch, M. J. Ab Initio Calculation of Vibrational Absorption and Circular Dichroism Spectra Using Density Functional Force Fields. *J. Phys. Chem*. **1994**, *98*, 11623–11627.

(7) Grimme, S.; Antony, J.; Ehrlich, S.; Krieg, H. A consistent and accurate ab initio parametrization of density functional dispersion correction (DFT-D) for the 94 elements H-Pu. *J. Chem. Phys*. **2010**, 132, 154104.

(7) Gaussian 09, Revision D.01, Frisch, M. J.; Trucks, G. W.; Schlegel H. B.; Scuseria, G. E.; Robb, M. A.; Cheeseman, J. R.; Scalmani, G.; Barone, V.; Mennucci, B.; Petersson, G. A.; Nakatsuji, H.; Caricato, M.; Li, X.; Hratchian, H. P.; Izmaylov, A. F.; Bloino, J.; Zheng, G.; Sonnenberg, J. L.; Hada, M.; Ehara, M.; Toyota, K.; Fukuda, R.; Hasegawa, J.; Ishida, M.; Nakajima, T.; Honda, Y.; Kitao, O.; Nakai, H.; Vreven, T.; Montgomery, J. A.; Peralta, Jr., J. E.; Ogliaro, F.; Bearpark, M.; Heyd, J. J.; Brothers, E.; Kudin, K. N.; Staroverov, V. N.; Keith, T.; Kobayashi, R.; Normand, J.; Raghavachari, K.; Rendell, A.; Burant, J. C.; Iyengar, S. S.; Tomasi, J.; Cossi, M.; Rega, N.; S43 Millam, J. M.; Klene, M.; Knox, J. E.; Cross, J. B.; Bakken, V.; Adamo, C.; Jaramillo, J.; Gomperts, R.; Stratmann, R. E.; Yazyev, O.; Austin, A. J.; Cammi, R.; Pomelli, C.; Ochterski, J. W.; Martin, R. L.; Morokuma, K.; Zakrzewski, V. G.; Voth, G. A.; Salvador, P.; Dannenberg, J. J.; Dapprich, S.; Daniels, A. D.; Farkas, O.; Foresman, J. B.; Ortiz, J. V.; Cioslowski, J.; Fox, D. J. Gaussian, Inc., Wallingford CT, 2013.

(8) Andrea, D.; Häußermann, U. M.; Dolg, M.; Stoll, H.; Preuss, H. Energyadjusted ab initio pseudopotentials for the second and third row transition elements. *Theor. Chim. Acta* **1990**, *77*, 123–141.

(9) Ehlers, A. W.; Bohme, M.; Dapprich, S.; Gobbi, A.; Hollwarth, A.; Jonas, V.; Kohler, K. F.; Stegmann, R.; Veldkamp, A.; Frenking, G. A set of f-polarization functions for pseudo-potential basis sets of the transition metals SC-Cu, Y-Ag and La-Au. *Chem. Phys. Lett*. **1993**, *208*, 111–114.

(10) (a) Hehre, W. J.; Ditchfield, R.; Pople, J. A. Self-Consistent Molecular Orbital Methods. XII. Further Extensions of Gaussian-Type Basis Sets for Use in Molecular Orbital Studies of Organic Molecules. *J. Chem. Phys*. **1972**, *56*, 2257–2261. (b) Francl, M. M.; Pietro, W. J.; Hehre, W. J.; Binkley, J. S.; Gordon, M. S.; DeFrees, D. J.; Pople, J. A.

Self-consistent molecular orbital methods. XXIII. A polarization-type basis set for second-row elements. *J. Chem. Phys*. **1982**, *77*, 3654–3665.

(11) Marenich, A. V.; Cramer, C. J.; Truhlar, D. G. Universal Solvation Model Based on Solute Electron Density and on a Continuum Model of the Solvent Defined by the Bulk Dielectric Constant and Atomic Surface Tensions. *J. Phys. Chem. B* **2009**, *113*, 6378–6396.

(12) O'Boyle, N. M.; Tenderholt, A. L.; Langner, K. M. cclib: A Library for Package-Independent Computational Chemistry Algorithms. *J. Comput. Chem*. **2008**, *29*, 839−845.

(13) ***NBO 7.0.*** E. D. Glendening, J, K. Badenhoop, A. E. Reed, J. E. Carpenter, J. A. Bohmann, C. M. Morales, P. Karafiloglou, C. R. Landis, and F. Weinhold, Theoretical Chemistry Institute, University of Wisconsin, Madison (2018).
